# Common Amino Acid Subsequences in a Universal Proteome—Relevance for Food Science

**DOI:** 10.3390/ijms160920748

**Published:** 2015-09-01

**Authors:** Piotr Minkiewicz, Małgorzata Darewicz, Anna Iwaniak, Jolanta Sokołowska, Piotr Starowicz, Justyna Bucholska, Monika Hrynkiewicz

**Affiliations:** Department of Food Biochemistry, University of Warmia and Mazury in Olsztyn, Plac Cieszyński 1, Olsztyn-Kortowo 10-726, Poland; E-Mails: darewicz@uwm.edu.pl (M.D.); ami@uwm.edu.pl (A.I.); jolanta_sokolowska133@wp.pl (J.S.); pointer86@wp.pl (P.S.); justyna.bucholska@uwm.edu.pl (J.B.); monika.protasiewicz@uwm.edu.pl (M.H.)

**Keywords:** allergens, biologically active peptides, biomarkers, epitopes, databases

## Abstract

A common subsequence is a fragment of the amino acid chain that occurs in more than one protein. Common subsequences may be an object of interest for food scientists as biologically active peptides, epitopes, and/or protein markers that are used in comparative proteomics. An individual bioactive fragment, in particular the shortest fragment containing two or three amino acid residues, may occur in many protein sequences. An individual linear epitope may also be present in multiple sequences of precursor proteins. Although recent recommendations for prediction of allergenicity and cross-reactivity include not only sequence identity, but also similarities in secondary and tertiary structures surrounding the common fragment, local sequence identity may be used to screen protein sequence databases for potential allergens *in silico*. The main weakness of the screening process is that it overlooks allergens and cross-reactivity cases without identical fragments corresponding to linear epitopes. A single peptide may also serve as a marker of a group of allergens that belong to the same family and, possibly, reveal cross-reactivity. This review article discusses the benefits for food scientists that follow from the common subsequences concept.

## 1. Introduction

Food science is being rapidly integrated with other areas, such as chemistry, biology, medicine or pharmacology. The ideas, methods and concepts originating in the above fields are applied to solve food-related problems. The concept of common subsequences creates new opportunities for analyzing problems in selected areas of interest related to food science.

A universal proteome [1] is defined as the set of all existing proteins. It is also referred to as a proteomosphere [2] or the protein universe [3].

A common subsequence is a fragment of the amino acid chain that occurs in more than one protein. Such a fragment may be regarded as a motif, *i.e.*, a reproducible pattern in a protein sequence that is attributed to a specific biological function [4]. Common subsequences may constitute continuous motifs. The role of short, continuous motifs in the biological functions of proteins and immunology constitutes the domain of peptidology [5]. Such short subsequences may play important roles as fragments of entire proteins (e.g., as epitopes responsible for interactions between proteins and antibodies) or after release through proteolytic enzymes (e.g., as hormones). In the latter case, short fragments may constitute “cryptides”, peptides that are encrypted in protein sequences, are inactive inside the protein chain and are activated after enzymatic release. This definition of bioactive peptides was introduced by Schlimme and Meisel [6] for products of food protein hydrolysis.

The shortest peptides containing two or three amino acid residues are especially interesting for food scientists because they pass from the gastrointestinal tract to the blood [7,8]. Affinity for small intestine isoforms of the oligopeptide transporter (target ID: CHEMBL4605) is emphasized in the ChEMBL database [9,10] as a standard property of dipeptides and tripeptides.

Compounds that are used as drugs or potential drugs are characterized by low molecular weight [11,12]. The criteria for the selection of potential drugs, referred to as the “Rule of five”, include molecular weight, number of H-bond donors, number of H-bond acceptors and hydrophobicity measured as the logarithm of the octanol/water partition coefficient [11]. The above parameters should not exceed the following values: molecular weight—500 Da, number of H-bond donors—5, number of H-bond acceptors—10, logarithm of the octanol/water partition coefficient (CLogP)—5 [11]. The shortest peptides that fulfill the above criteria are annotated in chemical databases, such as PubChem [13,14], ChemSpider [15,16] or ChEMBL, as potential objects of interest in pharmacology. Those criteria may also be applied to select potentially bioactive food components.

Every protein may be a source of biologically active dipeptides and tripeptides. The shortest sequences are most consistent with the hypothesis formulated by Karelin *et al.* [17], which states that all existing proteins are precursors of peptides whose biological activity is revealed after release.

Common subsequences are also used in comparative proteomics on the assumption that homologous proteins (which possess a common ancestor) can release similar sets of peptides during proteolysis. Comparative proteomics supports the search for non-sequenced proteins based on the presence of fragments representative of the protein family. Peptides are identified by mass spectrometry to detect a protein family that contains the same or similar fragments [2,18]. Peptides identified in such experiments should be characterized by the greatest possible length.

This review article discusses various aspects of common short fragments in proteins using the example of bioactive peptides, epitopes and protein biomarkers. The presented examples include proteins and peptides originating from organisms that are major food resources (e.g., wheat, cattle, chickens, fish) or microorganisms utilized in the food industry (yeasts).

## 2. Biologically Active Peptides

Biologically active peptides are involved in the regulation of many processes in living organisms. They may be produced in the body by synthesis or hydrolysis of precursors (endogenous peptides) or supplied with food (exogenous peptides). Peptides of the latter category constitute valuable components of functional foods, *i.e.*, foods with defined biological activity. Functional foods may support conventional treatments of selected diseases. Hypertension is the best known example of diseases that can be effectively mitigated by diet.

The most recent review article describing the state of the art in proteomics and peptidomics research relating to both categories of bioactive peptides was published by Dallas *et al.* [19]. Peptides and proteins can be identified with the use of standard proteomics or peptidomics techniques involving mass spectrometry [19,20]. Two questions have to be answered when a peptide or a protein is identified in an organism, cell, tissue or food product: which biologically active fragments are present in the analyzed protein or peptide and what are the possible precursors of the analyzed peptide? To answer the first question, data has to be interpreted in a manner similar to the top-down approach in proteomics [20]. This protocol begins with a search for the short fragments of protein sequence. To answer the second question, the peptide sequence should be used as a query, and the database of protein or peptide sequences should be searched for longer sequences containing the analyzed fragment.

**Figure 1:**
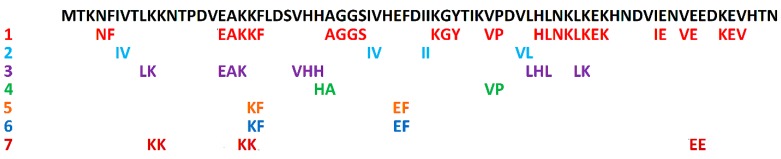
Location of biologically active fragments in the sequence of yeast (*Saccharomyces cerevisiae*, strain ATCC 204508/S288c) protease B inhibitor 2 (Accession No P0CT04 in the UniProt Knowledgebase [21,22]). (1) angiotensin I-converting enzyme (ACE) inhibitors; (2) glucose uptake stimulators; (3) antioxidant fragments; (4) dipeptidyl peptidase IV inhibitors; (5) calmodulin-dependent phosphodiesterase 1 inhibitors; (6) renin inhibitors; (7) fragments with other activities (see Table 1). Bioactive fragments were found with the use of the BIOPEP search engine [23,24] where the protein sequence was the query. The search was performed in May 2014.

The exemplary results of “top-down mimicking” search are presented in Figure 1 and Table 1. The sequence of protein from yeast (*Saccharomyces cerevisiae*), a microorganism that is broadly applied in food technology, was used as a query.

**Table 1:** Reference data for biologically active fragments of yeast (*Saccharomyces cerevisiae*, strain ATCC 204508/S288c) protease B inhibitor 2, indicated in Figure 1.

ID ^a^	Sequence ^b^	Activity	Primary Resource ^c^	Reference
3379	AKK	ACE inhibitor	Muscle of fish of the genus Sardina ^d^	[25]
3532	GY	ACE inhibitor	Synthetic	[26]
7587	VP	ACE inhibitor	Synthetic	[26]
7600	AG	ACE inhibitor	Synthetic	[26]
7602	HL	ACE inhibitor	Synthetic	[27]
7604	KG	ACE inhibitor	Synthetic	[26]
7607	GS	ACE inhibitor	Synthetic	[26]
7616	GG	ACE inhibitor	Synthetic	[27]
7623	EA	ACE inhibitor	Synthetic	[26]
7654	NKL	ACE inhibitor	Wakame (*Undaria pinnatifida*) ^d^	[28]
7683	NF	ACE inhibitor	Garlic (*Allium sativum*) ^d^	[28]
7692	KF	ACE inhibitor	Garlic (*Allium sativum*) ^d^	[28]
7693	KL	ACE inhibitor	Wakame (*Undaria pinnatifida*) ^d^	[28]
7698	NK	ACE inhibitor	Wakame (*Undaria pinnatifida*) ^d^	[28]
7827	IE	ACE inhibitor	Bovine (*Bos taurus*) milk ^d^	[29]
7828	EV	ACE inhibitor	Bovine (*Bos taurus*) milk ^d^	[29]
7829	VE	ACE inhibitor	Bovine (*Bos taurus*) milk ^d^	[29]
7832	LN	ACE inhibitor	Bovine (*Bos taurus*) milk ^d^	[29]
7840	EK	ACE inhibitor	Bovine (*Bos taurus*) milk ^d^	[29]
7841	KE	ACE inhibitor	Bovine (*Bos taurus*) milk ^d^	[29]
8320	VL	Glucose uptake stimulating	Bovine (*Bos taurus*) whey ^d^	[30]
8322	IV	Glucose uptake stimulating	Bovine (*Bos taurus*) whey ^d^	[30]
8325	II	Glucose uptake stimulating	Bovine (*Bos taurus*) whey ^d^	[30]
8329	EE	Vasoactive substance release stimulating	Soybean (*Glycine max*) ^d^	[31]
3305	LH	Antioxidant	Soybean (*Glycine max*) ^d^	[32]
3317	HL	Antioxidant	Soybean (*Glycine max*) ^d^	[32]
3319	HH	Antioxidant	Soybean (*Glycine max*) ^d^	[32]
7794	VHH	Antioxidant	Chicken (*Gallus gallus*) egg ^d^	[33]
7995	LHL	Antioxidant	Synthetic	[34]
8130	EAK	Antioxidant	Bonito (*Katsuwonus pelamis*) ^d^	[35]
8217	LK	Antioxidant	Chicken (*Gallus gallus*) egg ^d^	[36]
3751	KK	Bacterial permease ligand	Synthetic	[37]
3181	VP	Dipeptidyl peptidase IV inhibitor	Rat (*Rattus norvegicus*)	[38]
3184	HA	Dipeptidyl peptidase IV inhibitor	Rat (*Rattus norvegicus*)	[38]
8249	KF	CaMPDE inhibitor	Pea (*Pisum sativum*) ^d^	[39]
8250	EF	CaMPDE inhibitor	Pea (*Pisum sativum*) ^d^	[39]
8248	KF	Renin inhibitor	Pea (*Pisum sativum*) ^d^	[39]
8251	EF	Renin inhibitor	Pea (*Pisum sativum*) ^d^	[39]

^a^ ID number in the BIOPEP database; ^b^ Sequence given in a single-letter code; ^c^ Source from which the peptide was isolated for the first time; ^d^ Organism used as a food resource. Abbreviations used in Table 1: ACE—angiotensin I-converting enzyme; CaMPDE—calmodulin-dependent phosphodiesterase 1.

Among the bioactive peptides shown in Table 1, angiotensin I-converting enzyme (EC 3.4.15.1) inhibitors are most abundant. In the BRENDA database [40,41] the recommended name of the enzyme is peptidyl-dipeptidase A. The enzyme participates in the release of angiotensin II, a peptide that causes vasoconstriction. ACE inhibitors may thus lower blood pressure *in vivo* [42]. They are the most extensively studied class of bioactive peptides from food [42,43,44]. Renin (EC 3.4.23.15) inhibitors are also involved in blood pressure regulation. Renin releases the peptide angiotensin I from its precursor, angiotensinogen. Angiotensin I is inactive, but it is a substrate for conversion to the vasoconstrictor angiotensin II. Renin inhibitors pose an alternative to ACE inhibitors. They attract the interest of researchers as drugs [45] as well as bioactive components of functional foods that prevent hypertension [46,47]. Some peptides, including KF (Table 1), are capable of inhibiting the angiotensin-converting enzyme as well as renin. Peptides with sequences VP and HA are inhibitors of dipeptidyl peptidase IV (EC 3.4.14.5). This enzyme participates in the hydrolysis of the insulinotropic hormone, glucagon-like peptide 1. Due to this function, enzyme inhibitors can be used in the treatment of type II diabetes. Inhibitors of dipeptidyl peptidase IV may be used as anti-diabetic drugs [48] and components of functional foods designed for the treatment of diabetes [49]. Peptides with sequences KF and EF inhibit calmodulin-dependent phosphodiesterase 1 (EC 3.1.4.17). In the BRENDA database, the recommended name of the enzyme is 3′,5′-cyclic-nucleotide phosphodiesterase. The enzyme is involved in the metabolism of cyclic adenosine 3,5-monophosphate (cAMP) and regulation of cellular processes mediated by this compound. Inhibitors of 3′,5′-cyclic-nucleotide phosphodiesterase constitute potential treatment for cancer [50], inflammatory [51,52], autoimmune [51], and neurological diseases [52]. Antioxidant peptides from food, in particular short-chain peptides, are considered helpful in the prevention of oxidative damage [53]. Food components that stimulate glucose uptake (including peptides) are recommended for athletes [54].

Several examples of “top-down mimicking” database searches are shown in Table 2. Simulated proteolysis *in silico* of proteins from the human digestive tract [55] is included. All other examples presented in Table 2 are related to food. The BIOPEP database [23,24] was used in all cases, and query peptide or protein sequences were longer than the target sequences. The target sequences were short peptides (usually dipeptides and tripeptides) summarized in the database. Peptide sequences used as queries were obtained by mass spectrometry.

The examples presented in Table 2 account for only *in silico* and *in silico* together with experimental research. The second option involves mass spectrometry, followed by database screening. In addition to the examples shown in Table 2, short sequences (dipeptides and tripeptides) were also matched exactly in the database. In a food experiment conducted by Barba de la Rosa *et al.* [56], bioactive dipeptides and tripeptides were identified in hydrolysates of amaranthus proteins. In food science, *in silico* research also involved proteolysis simulations that seemed to be the weak point of experiments. The results of *in silico* and *in vitro* studies were compared to demonstrate differences between predicted and experimentally obtained patterns of proteolysis. The observed differences included both the absence of the predicted peptides [57] and the presence of peptides that were not expected to be released by enzymes with known specificity [58]. A successful prediction of an antimicrobial peptide released from casein by proteolysis has been recently described by Guinane *et al.* [59]. The noted differences could be explained by the fact that the specificity of proteolytic enzymes may be affected by reaction conditions, changes in protein structure and possible interactions with other compounds in the reaction environment.

An example of “bottom-up mimicking” (query sequence shorter than the target) search results is presented in Table 3.

**Table 2:** Examples of protocols involving the search for shorter fragments in sequences of proteins or peptides relevant for food and/or nutrition sciences.

Database Search Application	Reference
Location of short, bioactive fragments in sequences of peptides released during hydrolysis of bovine and trout meat proteins in the porcine digestive tract. Peptides used as query sequences were identified by mass spectrometry.	[60]
Location of bioactive fragments in sequences of rapeseed proteins. Protein sequences from UniProt were used as queries.	[61]
Location of bioactive fragments in sequences of bovine meat proteins. Protein sequences from UniProt were used as queries.	[62]
Location of short, bioactive fragments in sequences of peptides released during hydrolysis of fish sarcoplasmic proteins. Peptides used as query sequences were identified by mass spectrometry.	[63]
Location of bioactive fragments in sequences of cereal proteins. Protein sequences from UniProt were used as queries.	[64]
The BIOPEP database was used to determine the profiles of potential biological activity of salmon proteins. Some of the predicted peptides were identified in protein hydrolysates by liquid chromatography and mass spectrometry.	[58]
Location of bioactive fragments in sequences of proteins from the human digestive tract, followed by proteolysis simulation by digestive proteolytic enzymes. Protein sequences from UniProt were used as queries.	[55]
Location of bioactive fragments in sequences of amaranthus proteins. Protein sequences from UniProt were used as queries.	[65]

**Table 3:** Proteins containing fragment PANLPWGSSNV with an ACE inhibitory activity [66] (ID 49468 in the PepBank database [67,68]). The UniProt Knowledgebase [21,22] was screened with the BLAST program [69,70] with the use of the above sequence as a query and screening parameters described by Minkiewicz *et al.* [71]. The search was performed in May 2014.

No	Protein Name	Entry Name in UniProtKB	Organism ^a^
1.	Uncharacterized protein	TR:W4ZV89_WHEAT	*Triticum aestivum* (4565)
2.	Glyceraldehyde-3-phosphate dehydrogenase	SP:G3P3_YEAST	*Saccharomyces cerevisiae* (strain ATCC 204508/S288c) (559292)
3.	Glyceraldehyde-3-phosphate dehydrogenase	TR:A6ZUK2_YEAS7	*Saccharomyces cerevisiae* (strain YJM789) (307796)
4.	Glyceraldehyde-3-phosphate dehydrogenase	TR:B3LI45_YEAS1	*Saccharomyces cerevisiae* (strain RM11-1a) (285006)
5.	Glyceraldehyde-3-phosphate dehydrogenase	TR:B5VJD4_YEAS6	*Saccharomyces cerevisiae* (strain AWRI1631) (545124)
6.	Glyceraldehyde-3-phosphate dehydrogenase	TR:C8Z985_YEAS8	*Saccharomyces cerevisiae* (strain Lalvin EC1118 / Prise de mousse) (643680)
7.	Glyceraldehyde-3-phosphate dehydrogenase	TR:E7KD02_YEASA	*Saccharomyces cerevisiae* (strain AWRI796) (764097)
8.	Glyceraldehyde-3-phosphate dehydrogenase	TR:E7KP33_YEASL	*Saccharomyces cerevisiae* (strain Lalvin QA23) (764098)
9.	Glyceraldehyde-3-phosphate dehydrogenase	TR:E7LUX3_YEASV	*Saccharomyces cerevisiae* (strain VIN 13) (764099)
10.	Glyceraldehyde-3-phosphate dehydrogenase	TR:E7NI37_YEASO	*Saccharomyces cerevisiae* (strain FostersO) (764101)
11.	Glyceraldehyde-3-phosphate dehydrogenase	TR:E7Q4A2_YEASB	*Saccharomyces cerevisiae* (strain FostersB) (764102)
12.	Glyceraldehyde-3-phosphate dehydrogenase	TR:E7QF80_YEASZ	*Saccharomyces cerevisiae* (strain Zymaflore VL3) (764100)
13.	Glyceraldehyde-3-phosphate dehydrogenase	TR:G2WES0_YEASK	*Saccharomyces cerevisiae* (strain Kyokai no. 7/NBRC 101557) (721032)
14.	Tdh3p	TR:H0GGT7_9SACH	*Saccharomyces cerevisiae* x *Saccharomyces kudriavzevii* VIN7 (1095631)
15.	Uncharacterized protein	TR:J7S7S3_KAZNA	*Kazachstania naganishii* (strain ATCC MYA-139/BCRC 22969/CBS 8797/CCRC 22969/KCTC 17520/NBRC 10181/NCYC 3082) (1071383)
16.	Tdh3p	TR:N1P2H7_YEASC	*Saccharomyces cerevisiae* (strain CEN.PK113-7D) (889517)
17.	Tdh3p	TR:W7PUI3_YEASX	*Saccharomyces cerevisiae* R008 (1182966)
18.	Tdh3p	TR:W7RBG4_YEASX	*Saccharomyces cerevisiae* P283 (1177187)

^a^ Defined by the Latin name and NCBI taxonomic identifier [72,73] (in parentheses).

The query peptide originates from yeasts (*Saccharomyces cerevisiae*) [66]. All proteins presented in Table 3 belong to the Glyceraldehyde/Erythrose phosphate dehydrogenase family (Signature IPR020831 in the InterPro classification system [74,75]). The data shown in Table 3 illustrate the possibility of the same fragment occurring in homologous proteins from various microbial species and strains. This phenomenon is noted when the bioactive fragment occurs in a strongly conserved part of the protein chain. The observation that a biologically active peptide may possess more than one precursor is emphasized in the AHTPDB database of antihypertensive peptides [43,44].

Peptide LAPSLPGKPKPD (BIOPEP ID: 8388; 8547; 8548; 8550) may serve as an example of a peptide with a single known precursor. It was found (26 February 2015) only in the sequence of visual system homeobox 2 (Entry name in UniProt: VSX2_CHICK) from chicken (*Gallus gallus*) eggs. Similar fragments containing 8–10 of the 12 amino acid residues in the above peptide were found in sequences of five microbial enzymes annotated in the UniProt database. Peptide LAPSLPGKPKPD is multifunctional. It acts as an inhibitor of angiotensin I-converting enzyme (EC 3.4.15.1), dipeptidyl peptidase IV (EC 3.4.14.5), α-glucosidase (EC 3.2.1.20), and it has antioxidant properties [76]. A BLAST [69,70] search (19 March 2015) revealed that complete sequences of visual system homeobox 2 proteins are available for mammals, reptiles and fish. Partial sequences of rock pigeon (*Columba livia*; protein: TR:I1TED5_COLLI), turkey (*Meleagris gallopavo*; protein: TR:G1NJ43_MELGA) and mallard (*Anas platyrhynchos* protein: TR:U3J596_ANAPL) proteins were characterized by 98%–100% identity with chicken visual system homeobox 2 proteins. Therefore, a more comprehensive list of bird proteins could reveal a higher number of potential precursors of peptide LAPSLPGKPKPD. To date, the chicken genome and proteome have been studied most extensively in birds due to the significance of chicken as a food source. Chicken egg proteins are also studied as a source of peptides with various biological activities [77].

## 3. Linear Epitopes

Epitopes attract the interest of researchers dealing with three fields of science: allergology, immunochemical analysis methods (ELISA) and vaccinology. The first two areas also capture the interest of food scientists due to the prevalence of food allergies and the broad application of immunochemical methods in food analysis. Epitopes are defined as protein fragments responsible for interactions with the immune system (antibodies, B cells, T cells). Epitopes are divided into two classes: sequential (linear) epitopes which are continuous fragments of the primary protein structure, and conformational epitopes which are formed by neighboring amino acid residues on the surface of the antigen, but do not form a continuous fragment in the primary structure. Role of spatial structure of epitopes is recently emphasized even in the case of linear ones [78,79].

The standard protocol for the search of linear epitopes covers protein hydrolysis or synthesis of protein chain fragments, followed by experimental detection of interactions between specific peptides and antibodies of allergy sufferers. Albrecht and co-workers [78] pointed out that interactions between antibodies and fragments of synthetic proteins do not always lead to interactions with the same fragment encrypted in the entire protein sequence. The spatial structure of a short peptide may differ from the structure of the same peptide that is a part of a larger molecule. On the other hand, an example of a protein modified by insertion of a linear epitope and interaction with immunoglobulin E has been described [78].

Some allergenic proteins, such as caseins, which are major milk proteins, do not form compact or well-established spatial structures. In this case, allergenic properties may be retained under denaturing conditions (e.g., after heating, a process that is commonly applied in food processing) [80,81]. The presence of proteins and protein fragments that do not form a well-defined structure (“naturally denatured” proteins) is a relatively common phenomenon [82,83]. In the case of naturally denatured proteins or protein fragments, interactions between short peptide fragments and antibodies of allergenic patients imply interactions between entire proteins and, consequently, cross-reactivity of proteins containing the same fragment which is recognized as an epitope.

The experimental criteria for allergenic proteins recommended by the International Union of Immunological Societies (IUIS) were summarized by Breiteneder and Chapman [84]. An allergenic protein should meet a number of biochemical criteria, such as a known sequence and posttranslational modification pattern (if applicable), purification to homogeneity or near homogeneity, determination of basic physicochemical properties (molecular weight, isoelectric point) and production of monoclonal or monospecific antibodies that interact with the allergen. Immunological criteria of allergenicity include comparisons of the prevalence of serum IgE antibodies in as many patients as possible (at least 50 are recommended), determination of allergenic activity (e.g., skin tests), reducing IgE binding capacity of the allergenic extract after allergen removal (e.g., by immunoabsorption) and detection of IgE binding ability of a recombinant allergen. Proteins whose sequences have not been confirmed experimentally, but predicted based on sequence or structure analysis, are sometimes regarded as allergens *in silico*. The simplest method of determining *in silico* allergenicity and predicting cross-reactivity involves a comparison of the sequence of the analyzed protein with experimentally confirmed allergens. A protein is a potential allergen if it contains a fragment with at least six to eight amino acid residues which are identical to the fragment of a known allergen, or a fragment of at least 80 amino acid residues with a minimum 35% identity with a fragment of the known allergen [85,86,87]. The presence of common sequential epitopes (as long as possible) seems to increase the likelihood of cross-reactivity [88,89]. Common sequential epitopes are usually present in homologous proteins, *i.e.*, proteins that have a common ancestor and belong to the same family. Homologous proteins possess similar amino acid sequences and similar structure. Protein families are classified based on the presence of characteristic domains that are attributed to protein functions [90]. Protein families are described in domain databases such as InterPro [74,75] and Pfam [91,92]. The AllFam database of allergen families [93,94] was developed based on the protein classification system found in the Pfam database. Fragments containing five amino acid residues are the smallest units that interact with the immune system [95]. The distribution of pentapeptides in the universal proteome was analyzed by *in silico* studies [95,96,97]. Tools for comparing protein sequences and identifying common pentapeptides and other common motifs have been recently developed [98,99,100,101].

In this review article, the possible distribution of epitopes across protein families is discussed based on 4 epitopes from wheat (*Triticum aestivum*) ω5-gliadin (UniProt entry name: Q402I5_WHEAT) [102]. Modifications of those epitopes have been proposed to significantly decrease gliadin allergenicity [103]. The discussed epitopes have the following amino acid sequences: QQFPQQQ (IEDB ID 52028), QQIPQQQ (IEDB ID 52043), QQLPQQQ (IEDB ID 52066) and QQYPQQQ (IEDB ID 52180). Numbers in parentheses indicate ID numbers in the Immune Epitope Database (IEDB) [104,105]. The search protocol was identical to that whose results are presented in Table 3. Wheat and related species are among the most commonly used resources in the food industry. Gliadins and their homologs from other cereals belong to the best known group of food proteins.

Due to space constraints, the table containing a list of proteins and fragments identical to gliadin epitopes is presented in the form of a supplement. The supplement includes proteins identified “at protein level” (e.g., by mass spectrometry) as well as amino acid sequences translated from the known nucleotide sequences (putative or identified at transcript level). It also contains a table of protein families defined according to the InterPro database (with links to records of particular domains), species annotated by their Latin names and taxonomic identifiers (with link to annotations of species on the UniProt website) and proteins annotated based on their entry names in UniProt (with links to particular records). Four peptides have been listed based on amino acid sequences in a single-letter code and chemical identifiers: SMILES [106], InChI and InChIKeys [107]. As previously noted [108] peptide structures annotated with the SMILES code may be used as input in many cheminformatics programs. They are used in specialized peptide databases such as AHTPDB [44]. Links to peptide databases deploying chemical codes (SMILES) are available on the MetaComBio website [109,110]. InChI and InChIKey offer more advantages in comparison with SMILES. Few versions of annotation of the same structure are possible using the last code. SMILES requires special search engines, whereas InChI and especially InChIKey are more versatile and may be used as queries in popular search engines such as Google™ [111]. Peptide structures described with InChI and InChIKey are thus more effective in identifying datasets (such as the supplement to this publication) than sequences written in a single-letter code. Peptides are commonly annotated with chemical codes in chemical databases such as ChEMBL, ChemSpider and PubChem. InChiKeys are also used in the BRENDA database [40,41]. The amino acid sequences presented in the supplement were translated from amino acid sequences in FASTA format into chemical codes using the Open Babel program [112,113].

The number of proteins containing the above-mentioned sequences is high due to the fact that repeated glutamine residues belong to the most common motifs in known protein sequences. For instance, according to the database associated with the Tachyon program, the fragment containing five glutamine residues is present in more than half a million sequences [98,99].

**Figure 2:**
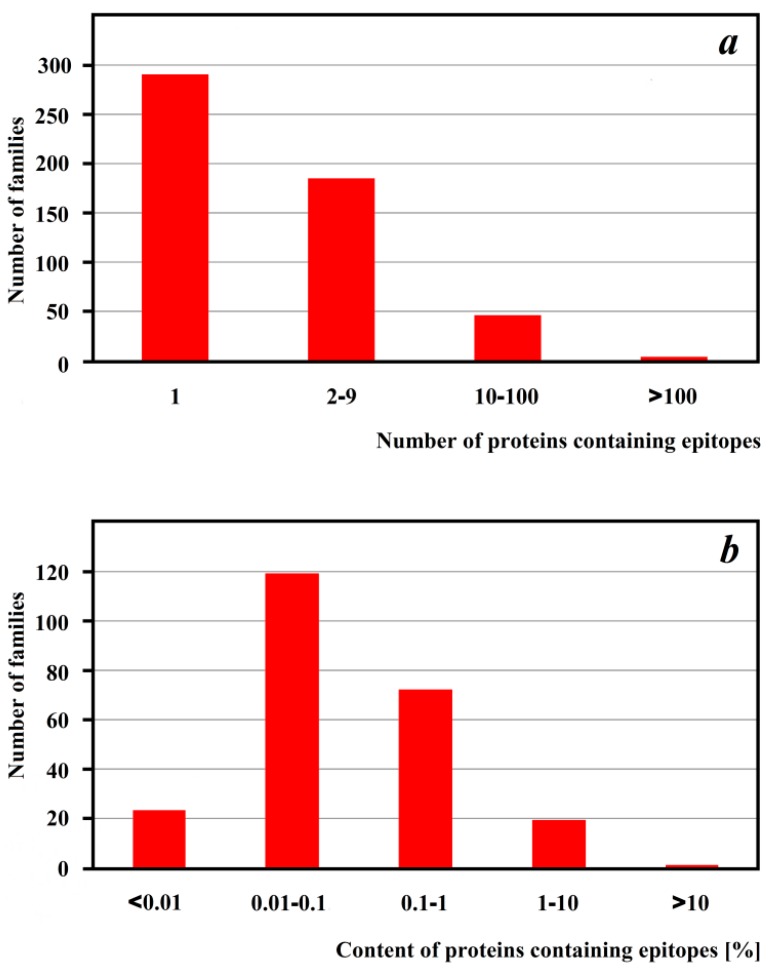
Distribution of proteins containing at least one of the four IgE-binding epitopes of ω5-gliadin [102] across protein families. (**a**) distribution based on the number of proteins containing epitopes in the family; (**b**) percentage content of proteins with epitopes in the family. The data for families containing at least two proteins with epitopes are shown in **b**.

Heptapeptides are distributed across hundreds of protein families (Figure 2), although in most cases, only several proteins in a family contain at least one epitope. Epitopes are most abundant in four protein families: gliadin, alpha/beta (IPR001376) (208 proteins), gliadin /low molecular weight glutenin (IPR001954) (199 proteins), bifunctional trypsin/alpha-amylase inhibitor helical domain (IPR013771) (199 proteins) and bifunctional inhibitor/plant lipid transfer protein/seed storage helical domain (IPR016140) (197 proteins). These families are characterized by a high ratio of epitope-containing sequences to proteins at 12.72%, 9.57%, 5.63% and 2.79%, respectively. They contain proteins that are closely related to ω5-gliadin, the first discovered precursor of the discussed epitopes.

In our previous publications, we discussed the distribution of specific epitopes at the level of protein families based on the presence of the corresponding domains rather than individual proteins [71,114]. Such choice is explained by the fact that the number of known protein sequences grows rapidly, but the number of known domains remains much more stable [3]. The observed increase in the number of protein families can be attributed to the discovery of new multidomain families. The probability that a protein contains an epitope together with a domain defining its family and function seems to be a more reliable determinant of epitope distribution, and it can be used as a prognostic tool. That probability may be expressed as the percentage of proteins within a family (with a domain defining the family), which contain an epitope (or another fragment of interest). A family may be defined in accordance with the rules of InterPro, Pfam or AllFam databases. Proteins belonging to the same family possess similar sequences, spatial structure and, consequently, similar physico-chemical properties (e.g., solubility under various conditions, susceptibility to thermal denaturation) that affect behavior during food processing. Proteins belonging to the same family can be expected to have a similar pattern of bioactive fragments.

There are two possible patterns of distribution of fragments that are identical to known epitopes. The hexapeptides from Baltic cod (*Gadus morhua* subsp. *callarias*) parvalbumin are distributed randomly across many protein families. None of them is present in other parvalbumins [71]. The epitopes from shrimp (*Farfantepenaeus aztecus*) tropomyosin, which contain 10–15 amino acid residues, occur in homologs of their precursor (other tropomyosins). Only one fragment with five residues is broadly distributed across various protein families. Two fragments containing eight amino acid residues were present in several proteins that did not belong to the tropomyosin family [114].

The traditional system for the classification of allergens includes the route of exposure (ingestion, inhalation, injection or contact), although several different routes can exist for the same allergen [115]. Routes of exposure for particular allergens are annotated e.g. in Allergome database [116,117]. Allergens are also divided into the following groups: food, indoor, outdoor and injected [84]. Organisms that synthesize proteins containing fragments identical to known epitopes may be classified based on the possibility of human contact [71,114]. Species that synthesize proteins containing fragments identical to linear epitopes may be divided into the following categories: animal and plant species relevant for the food industry and/or agriculture (mainly edible), microorganisms that are useful and potentially useful for the food industry and/or agriculture (e.g., used in biotechnological processes), human symbionts and commensals (e.g., gut microorganisms) as well as human pathogens and parasites [71]. The first two groups may be interesting from the point of view of food safety. A more detailed classification has been proposed in an article describing the distribution of fragments identical to shrimp (*Farfantepenaeus aztecus*) tropomyosin epitopes [114]. Invertebrates that synthesize tropomyosins containing fragments identical to the above epitopes belong to the following categories: edible invertebrates (crustaceans and molluscs), human parasites (e.g., worms), parasites of edible animals and plants (potential food contaminants), as well as species that come into contact with humans by other ways (indoor organisms such as dust mites and invertebrates cultured in laboratories, such as *Caenorhabditis elegans* or *Drosophila melanogaster*).

The enclosed supplement contains information about organisms belonging to all of the above categories (not only edible organisms or organisms used in food technology). The most abundant protein families are found in wheat and other cereals. Fragments identical to wheat gliadin epitopes were also found in the proteins of edible birds (e.g., *Gallus gallus*) or fish (*Takifugu rubripes*, *Oreochromis niloticus*). Yeast (*Saccharomyces cerevisiae*) is a model microorganism that is used in the food industry and contains proteins with fragments identical to the epitopes from wheat gliadin. Darewicz *et al.* [118] reported local sequence similarity between proteomes of wheat (*Triticum aestivum*) and yeast (*Saccharomyces cerevisiae*), where the yeast proteome contained short sequence fragments similar to celiac-toxic peptides. Vojdani and Tarash [119] observed interactions between yeast proteins and antibodies of patients suffering from celiac disease. Proteins from those species revealed cross-reactions with the human immune system.

Database screening may produce results that go beyond the area of interest in food science. The resulting data could also be interesting from the point of view of biological and medical sciences. *Candida albicans* is an example of a commensal microorganism that is ubiquitous in the human gut, but may cause opportunistic infections. This microorganism may be thus classified in two categories as a commensal and a pathogen [120]. *Candida albicans* proteins contain fragments identical to fragments of wheat (Table S1) as well as cod parvalbumins [71]. Protein sequences of the human parasite *Trichinella spiralis* contain short subsequences that are identical to the fragments of three allergenic proteins: wheat gliadin (Table S1), cod parvalbumin [71] and shrimp tropomyosin [114]. Fragments identical to wheat gliadin epitopes are also present in human protein sequences. Kanduc described [96] the degree of identity between allergenic epitopes (e.g., from food allergens) and human proteins at the pentapeptide level. Amino acid subsequences identical to parvalbumin and tropomyosin fragments were are also found in the human proteome [71,114]. Human tropomyosin, which contains a fragment identical to the shrimp allergen, is regarded as an autoantigen [121].

## 4. Peptides Relevant as Allergen Markers

Mass spectrometry is the recommended method for identifying and determining allergenic proteins in foods. Some of them are major food components. The presence of such proteins in food products may also result from contamination or adulteration. Qualitative and quantitative analyses of food allergens are based on the identification of peptides representative of allergens and considered as allergen markers (signatures) [122,123,124]. Peptides are usually released by trypsin (EC 3.4.21.4), an enzyme that is widely used in proteomics [122,123,124,125]. Recent experiments conducted with the use of mass spectrometry were described by Pilolli *et al.* [126], Gomaa and Boye [127] and by Posada-Ayala *et al.* [128]. Koeberl *et al.* [124] discussed the advantages of mass spectrometry for allergen analysis, including short time of analysis and the option of identifying multiple allergens in a single analysis. Some authors [129,130] recommended protein fragments overlapping with linear epitopes as markers for mass spectrometry. In this approach, the same fragments can be used in mass spectrometry and immunochemical methods, which is an unquestioned advantage.

The unique character of peptides (presence of a fragment in a single precursor) is emphasized as a major advantage in analyses that rely on the identification of protein fragments [131]. The rapid increase in the number of protein sequences annotated in UniProt [21,22], NCBI [132,133] or Allergome [116,117] makes this recommendation increasingly difficult to fulfill. Peptides used as markers may be released from multiple precursors, as demonstrated in the examples in Table 4.

Both peptides listed in Table 4 are fragments of multiple proteins from several species. The criteria for the choice of protein markers [131] indicate that an identified peptide may originate from proteins that are unlikely to be present in foods. Such proteins may originate from species that are not edible, wild, not used as sources of industrially processed foods or inhabit limited areas. In Table 4, such species are represented by wild birds from south-east Asia: *Gallus lafayetii* and *Gallus sonneratii*. The milk of the yak (*Bos mutus*) and water buffalo (*Bubalus bubalis*) as well as quail (*Coturnix coturnix japonica*) eggs are used as food, but they are less popular than cow milk and chicken eggs. The likelihood that yak or buffalo milk proteins will be identified in food products depends on their geographical origin. Peptide FFVAPFPEVFGK may indicate the presence of bovine α_s1_-casein in products originating from Europe as well as the presence of yak or buffalo proteins in products from central or Southeast Asia.

α_s1_-Casein from *Bos mutus* and lysozyme C from *Coturnix coturnix japonica* are not annotated in Allergome (as checked 28 April 2015), but they share linear epitopes with bovine (*Bos taurus*) α_s1_-casein and chicken (*Gallus gallus*) lysozyme C, respectively. Both proteins can be classified as allergens *in silico* according to criteria that are based on local sequence identity, including common fragments containing at least six to eight amino acid residues [85,86] or common, experimentally found epitopes [89].

Proteins which are the precursors of peptide FFVAPFPEVFGK belong to the following families: casein (IPR001588) and α_s1_-casein (IPR026999) in the InterPro database [74,75]; casein (PF00363) in the Pfam database [91,92] and alpha/beta casein (AF065) in the AllFam database [93,94]. Proteins containing the FESNFNTQATNR fragment belong to families: Glyco_hydro_22 (IPR001916), Glyco_hydro_22_CS (IPR019799), Glyco_hydro_22_lys (IPR000974), Lysozyme-like_dom (IPR023346) and Lysozyme_C (IPR030056) in the InterPro database, Lys (PF00062) in Pfam database and C-type lysozyme/alpha-lactalbumin family (AF016) in the AllFam database. In both cases, the group of precursors of a given peptide marker includes only a part of the protein family. The same applies to group markers predicted *in silico* [134] as well as common epitopes [71,114]. The group of proteins identified or determined in a single marker (signature) peptide should be precisely defined and updated to track the increase in the number of known protein sequences.

The presence of conserved fragments in a family creates new analytical opportunities. The same fragment may be present in proteins with and without a known sequence. The strategy that relies on local identity or similarity between sequenced and non-sequenced proteins is referred to as comparative proteomics [2,18]. Numerous edible organisms have not been subject to extensive studies aimed at protein sequencing to date. Edible insects, emerging as novel food resources [135,136], could also constitute a source of such proteins. Arthropod tropomyosins are allergens. Tropomyosins from various arthropods contain many identical fragments [114]. It is likely that selected peptides—markers of crustacean tropomyosins—may be used to detect allergens in insects. This could also apply to allergens from other sources.

**Table 4:** Proteins containing fragments FFVAPFPEVFGK and FESNFNTQATNR, used as markers of α_s1_-casein from milk and lysozyme from eggs, respectively [126]. The UniProt Knowledgebase [21,22] was screened with the BLAST program [69,70] with the use of the above sequence as a query and screening parameters described by Minkiewicz *et al.* [71]. The search was performed in April 2015.

No	Entry Name in UniProtKB	Allergome Annotation	Organism ^a^
Peptide (R)FFVAPFPEVFGK ^b^—marker of α_s1_-casein			
1.	CASA1_BOVIN	Bos d 9.0101; Code 10197	*Bos taurus* (9913)
2.	CASA1_BUBBU	Bub b 8; Code 1259	*Bubalus bubalis* (89462)
3.	G3C8Y4_BUBBU		*Bubalus bubalis* (89462)
4.	B5B3R8_BOVIN	Bos d 9; Code 2734	*Bos taurus* (9913)
5.	L8I5S0_9CETA		*Bos mutus (Bos grunniens)* (72004)
6.	G3C8Y5_BUBBU		*Bubalus bubalis* (89462)
7.	Q4F6X6_BUBBU		*Bubalus bubalis* (89462)
Peptide (K)FESNFNTQATNR ^c^—marker of lysozyme C			
1.	LYSC_CHICK	Gal d 4.0101; Code 3294	*Gallus gallus* (9031)
2.	LYSC_COTJA		*Coturnix coturnix japonica* (93934)
3.	B8YK77_GALLA	Gal la 4; Code 9143	*Gallus lafayetii* (9032)
4.	B8YK75_GALSO	Gal so 4; Code 9144	*Gallus sonneratii* (9033)
5.	B8YK79_CHICK	Gal d 4; Code 362	*Gallus gallus* (9031)
6.	B8YJP1_CHICK	Gal d 4; Code 362	*Gallus gallus* (9031)
7.	B8YJN9_CHICK	Gal d 4; Code 362	*Gallus gallus* (9031)
8.	B8YJT7_CHICK	Gal d 4; Code 362	*Gallus gallus* (9031)

^a^ Defined by the Latin name and NCBI taxonomic identifier [72,73] (in parentheses); ^b^ Fragment preceded by arginine residue in the sequences of all proteins annotated in the Table. The preceding residue (in parentheses) was included in the query sequence; ^c^ Fragment preceded by lysine residue in the sequences of all proteins annotated in the Table. The preceding residue (in parentheses) was included in the query sequence.

## 5. Mass Spectrometry as a Tool for Experimental Identification of Common Subsequences

Experimental proteomics or peptidomics studies (relating to food and nutrition) require the identification of peptide sequences. Mass spectrometry is a popular identification tool. The significance of mass spectrometry in research into proteins and their fragments was emphasized and extensively discussed in several reviews [7,19,122,123,124,125,137,138,139,140]. Almost all peptide sequences listed in databases and discussed in bioinformatics studies, including in this review article, were identified by mass spectrometry. There are no special mass spectrometric techniques that support the search for common subsequences. The question “Is this subsequence common?” requires bioinformatics tools.

Several practical applications of mass spectrometry in food peptide analyses are presented in Table 5.

**Table 5:** Selected applications of mass spectrometry for the identification of food peptides.

Aim of the Experiment	Mass Spectrometry Technique	Separation Method	Reference
Identification of Angiotensin I-converting enzyme (ACE) inhibitory peptides released during simulated gastrointestinal digestion of salmon (*Salmo salar*) muscles	ESI-IT-MS/MS, SRM	RP-HPLC, low TFA concentration in mobile phase	[58]
Detection and quantitative determination of peptides that are markers of bovine (*Bos taurus*) casein and chicken (*Gallus gallus*) egg proteins	ESI-MS/MS, SRM	RP-HPLC	[126]
Detection and quantitative determination of peptides that are markers of mustard allergen Sin a 1 in foods	ESI-MS/MS, SRM	RP-HPLC	[128]
Identification of peptides from peanut (*Arachis hypogaea*) allergens	nano-ESI Q-TOF MS/MS	capillary RP-HPLC	[129]
Identification of peptides from soybean (*Glycine max*) allergens	MALDI-TOF and MALDI-TOF-TOF	RP-HPLC	[130]

Abbreviations used in Table 5: ESI: electrospray ionization; IT: ion trap; MALDI: Matrix-Assisted Laser Desorption Ionization; MS: mass spectrometry; MS/MS: tandem mass spectrometry; Nano-ESI: nanoelectrospray; Q-TOF: quadrupole-time-of-flight; RP-HPLC: reversed-phase high-performance liquid chromatography; SRM: selected reaction monitoring; TFA: trifluoroacetic acid; TOF: time of flight.

Mass spectrometry protocols used for peptide identification include peptide fragmentation to determine the complete or partial sequence. Various tandem mass (MS/MS) techniques are used for this purpose, including triple quadrupole or ion trap. Electrospray (ESI) is the most popular peptide ionization method, and matrix-assisted laser desorption (MALDI) is also commonly used. Reversed-phase high-performance liquid chromatography with a water/acetonitrile mobile phase is usually applied as a method for on-line separation in combination with mass spectrometry. Trifluoroacetic acid (TFA), used as the third mobile phase component causes quenching electrospray ionization. Protocols with low TFA concentration in the mobile phase are thus developed. Formic acid may be also used as a mobile phase component. Formic acid produces mass spectra of excellent quality, but the quality of the resulting chromatograms is low. MALDI-MS is applied off line with RP-HPLC without any restrictions concerning trifluoroacetic acid concentrations. The MALDI ionization technique is more resistant to the presence of inorganic salts than ESI. On-line capillary electrophoresis with mass spectrometry is also used in proteomics and peptidomics [139]. The selected reaction monitoring (SRM) method supports quantitative analyses of peptides. It involves measurements of peak intensity corresponding to selected fragment ion or ions from the peptide of interest [126,128]. The SRM method involving more fragment ions from a single peptide may be used for peptide identification [58].

## 6. Final Remarks

Common amino acid subsequences occur in numerous proteins. This phenomenon should be taken into consideration in food science. The point for discussion is: Are common subsequences “friends” or “foes” of food scientists?

In biologically active peptides, the presence of common subsequences creates new opportunities for experimental design. Experiments involving bioactive peptides may be designed to search for new active compounds or known compounds in peptide mixtures [7]. The first strategy involves the separation of peptide mixtures into fractions, measurements of peptide activity, determination of amino acid sequences in active fraction compounds and confirmation of biological activity with the use of synthetic peptides. In the second strategy, peptides are identified be screening databases based on identified sequences as queries or by predicting peptide release. Examples of such experiments are summarized in Table 2. The search for novel active peptides and the existing databases may be significantly enhanced by high-throughput screening of peptide libraries. An experiment of the type has been recently described by Lan *et al.* [141]. They constructed a library of 367 dipeptides and screened it for compounds inhibiting dipeptidyl peptidase IV. The active peptides discovered via similar experiments may be found in food protein sequences and identified among products of their hydrolysis. Chanput *et al.* [142] predicted the biological activity of tripeptides, determined their location in protein sequences and simulated proteolysis. In regard to longer peptides which contain at least six amino acid residues, protein databases may be screened with the use of peptide sequences as queries to identify novel precursors and sources of bioactive peptides. Such protocols may support research into novel resources that can be potentially used in the production of functional foods.

The presence of common linear epitopes was recommended as a criterion of allergenicity and cross-reactivity prediction [89]. Recent recommendations to consider proteins as allergens *in silico* are more rigorous, and they account for the spatial structure of epitopes, even if they are sequential [79]. We can achieve consensus that protein database screening using epitope sequences as query may serve for construction of preliminary lists of potential allergens. They can be thus subjected to structure modeling *in silico* and finally to experiments aimed on fulfilling criteria summarized in a review published by Breiteneder and Chapman [84]. The structure and properties of the closest neighbor may be taken into account for the shortest sequences that are regarded as epitopes (pentapeptides). Common epitopes are particularly often found in conserved protein families such as tropomyosins [114]. This protein family is also characterized by conserved spatial structure. The presence of common linear epitopes is emphasized in databases such as the BIOPEP database of allergenic proteins and their epitopes [134] and Immune Epitope Database [105]. The latter contains a program that searches for common epitopes in a user-defined set of protein or peptide sequences. As previously noted [89,114] cross-reactivity between the allergens is also possible without identical epitopes or any identical fragments. This is a weak point of allergenicity and cross-reactivity predictions based on common subsequences.

In the context of the search for allergen markers, the presence of common subsequences may be considered as a weakness that obstructs the identification of a peptide signature of a unique protein. Despite the above, common subsequences create new opportunities for finding peptides that are markers of more than one protein. A single peptide may be a marker of a group of cross-reacting peptides. It would be interesting to use a single peptide as a marker of proteins with both known and unknown sequence according to the paradigm of comparative proteomics [18]. Chassaigne *et al.* [129] and Cucu *et al.* [130] recommend the use of peptide markers that overlap with epitopes, and such analyses would also create new opportunities. The same fragment can be used as a marker of a group of proteins identified by mass spectrometry and a marker of the same group of proteins detected by immunochemical methods.

The preparation and presentation of data relating to peptide sequences analyzed in single experiments or multiple precursors of single peptides may be fraught with problems. The process of updating major databases by insertion of hundreds of bioactive peptide sequences from a single protein chain or hundreds of precursors of single peptides may be difficult in real time. Publication of data in datasets such as the enclosed supplement creates useful opportunities for researchers. It is recommended that such datasets contain references or links to major databases (UniProt, Allergome, BIOPEP, IEDB, *etc.*) to provide as much information as possible in compact form. Data may be published in the form of supplements to articles or as separate datasets that are uploaded for instance on the websites of the authors' institutions. The use of chemical codes (InChI, InChiKey) for encoding peptides, in particular short peptides containing two or three amino acid residues, as recommended by Southan [111] may make finding of such datasets easier.

In this review we discussed the benefits for food scientist that follow from the use of common subsequences in the universal proteome and their relevance for food science. This phenomenon seems to be well known in biologically active peptides, where it has been discussed in the example of common epitopes, but it has not yet been analyzed in fragments that are protein markers.

## Supplementary Information

Supplementary materials can be found at http://www.mdpi.com/1422-0067/16/09/20748/s1.
