# Supplementary material for: Common Amino Acid Subsequences in a Universal Proteome—Relevance for Food Science
Source: Int J Mol Sci. 2015 Sep 1;16(9):20748–73. doi: 10.3390/ijms160920748 (PMC4613229; doi:10.3390/ijms160920748)
Supplement: Supplementary file 1 [file ijms-16-20748-s001.pdf]

Supplementary Information

Epitopes

QQFPQQQ (IEDB ID 52028)

SMILES:
N[C@H](C(=O)N[C@H](C(=O)N[C@H](C(=O)N1[C@H](C(=O)N[C@H](C(=O)N[C@H](C(=O)N[C@H](C(=O)O)CCC(=O)N)CCC(=O)N)CCC(=O)N)CCC1)Cc1ccccc1)CCC(=O)N)CCC(=O)N

InChI=1S/C39H58N12O13/c40-21(8-13-28(41)52)33(57)46-22(9-14-29(42)53)36(60)50-26(19-20-5-2-1-3-6-20)38(62)51-18-4-7-27(51)37(61)48-24(11-16-31(44)55)34(58)47-23(10-15-30(43)54)35(59)49-25(39(63)64)12-17-32(45)56/h1-3,5-6,21-27H,4,7-19,40H2,(H2,41,52)(H2,42,53)(H2,43,54)(H2,44,55)(H2,45,56)(H,46,57)(H,47,58)(H,48,61)(H,49,59)(H,50,60)(H,63,64)/t21-,22-,23-,24-,25-,26-,27-/m0/s1

InChIKey: NYDPXIRXHABUPK-DUJSLOSMSA-N

QQIPQQQ (IEDB ID 52043)

SMILES:
N[C@H](C(=O)N[C@H](C(=O)N[C@H](C(=O)N1[C@H](C(=O)N[C@H](C(=O)N[C@H](C(=O)N[C@H](C(=O)O)CCC(=O)N)CCC(=O)N)CCC(=O)N)CCC1)[C@H](CC)C)CCC(=O)N)CCC(=O)N

InChI=1S/C36H60N12O13/c1-3-17(2)29(47-33(57)21(9-14-27(41)52)43-30(54)18(37)6-11-24(38)49)35(59)48-16-4-5-23(48)34(58)45-20(8-13-26(40)51)31(55)44-19(7-12-25(39)50)32(56)46-22(36(60)61)10-15-28(42)53/h17-23,29H,3-16,37H2,1-2H3,(H2,38,49)(H2,39,50)(H2,40,51)(H2,41,52)(H2,42,53)(H,43,54)(H,44,55)(H,45,58)(H,46,56)(H,47,57)(H,60,61)/t17-,18-,19-,20-,21-,22-,23-,29-/m0/s1

InChIKey: OOICIMLPRJAEGU-GGAYEWCCSA-N

QQLPQQQ (IEDB ID 52066)

SMILES:
N[C@H](C(=O)N[C@H](C(=O)N[C@H](C(=O)N1[C@H](C(=O)N[C@H](C(=O)N[C@H](C(=O)N[C@H](C(=O)O)CCC(=O)N)CCC(=O)N)CCC(=O)N)CCC1)CC(C)C)CCC(=O)N)CCC(=O)N

InChI=1S/C36H60N12O13/c1-17(2)16-23(47-33(57)19(6-11-26(39)50)43-30(54)18(37)5-10-25(38)49)35(59)48-15-3-4-24(48)34(58)45-21(8-13-28(41)52)31(55)44-20(7-12-27(40)51)32(56)46-22(36(60)61)9-14-29(42)53/h17-24H,3-16,37H2,1-2H3,(H2,38,49)(H2,39,50)(H2,40,51)(H2,41,52)(H2,42,53)(H,43,54)(H,44,55)(H,45,58)(H,46,56)(H,47,57)(H,60,61)/t18-,19-,20-,21-,22-,23-,24-/m0/s1

InChIKey: HBTIKQRSQNKVSN-LQDRYOBXSA-N

QQYPQQQ (IEDB ID 52180)

SMILES:
N[C@H](C(=O)N[C@H](C(=O)N[C@H](C(=O)N1[C@H](C(=O)N[C@H](C(=O)N[C@H](C(=O)N[C@H](C(=O)O)CCC(=O)N)CCC(=O)N)CCC(=O)N)CCC1)Cc1ccc(cc1)O)CCC(=O)N)CCC(=O)N

InChI=1S/C39H58N12O14/c40-21(7-12-28(41)53)33(58)46-22(8-13-29(42)54)36(61)50-26(18-19-3-5-20(52)6-4-19)38(63)51-17-1-2-27(51)37(62)48-24(10-15-31(44)56)34(59)47-23(9-14-30(43)55)35(60)49-25(39(64)65)11-16-32(45)57/h3-6,21-27,52H,1-2,7-18,40H2,(H2,41,53)(H2,42,54)(H2,43,55)(H2,44,56)(H2,45,57)(H,46,58)(H,47,59)(H,48,62)(H,49,60)(H,50,61)(H,64,65)/t21-,22-,23-,24-,25-,26-,27-/m0/s1

InChIKey: XXLBCMJCIBJTLE-DUJSLOSMSA-N

Table S1. List oproteins containing epitopes from wheat ω-gliadin.

Domain names	Domain signatures in InterPro	Number of proteins containing domain	Number of proteins containing query sequence or sequences	Species	NCBI Taxonomy Identifiers (according to UniProt)	Proteins (entry names in UniProt)
Bromodomain, conserved site	IPR018359	3547	7	*Danio rerio*	7955	TR:Q1MT59_DANRE
						TR:F1R0I4_DANRE
						TR:A3KQN2_DANRE
				Takifugu rubripes	31033	TR:H2U5B7_TAKRU
						TR:H2U5B6_TAKRU
				Oreochromis niloticus	8128	TR:I3KHG7_ORENI
						TR:I3KHG6_ORENI
STAT transcription factor, DNA-binding	IPR013801	716	6	*Branchiostoma floridae*	7739	TR:C3ZUG6_BRAFL
				Debaryomyces hansenii	284592	TR:Q6BJE1_DEBHA
				Vanderwaltozyma polyspora	436907	TR:A7TQI4_VANPO
				Caenorhabditis elegans	6239	SP:P34545-3
						SP:P34545-2
						SP:CBP1_CAEEL
Domain of unknown function DUF902, CREBbp	IPR010303	215	7	*Danio rerio*	7955	TR:Q1MT59_DANRE
						TR:F1R0I4_DANRE
						TR:A3KQN2_DANRE
				Takifugu rubripes	31033	TR:H2U5B7_TAKRU
						TR:H2U5B6_TAKRU
				Oreochromis niloticus	8128	TR:I3KHG7_ORENI
						TR:I3KHG6_ORENI
Histone H3-K56 acetyltransferase, RTT109	IPR013178	576	7	*Danio rerio*	7955	TR:Q1MT59_DANRE
						TR:F1R0I4_DANRE
						TR:A3KQN2_DANRE
				Takifugu rubripes	31033	TR:H2U5B7_TAKRU
						TR:H2U5B6_TAKRU
				Oreochromis niloticus	8128	TR:I3KHG7_ORENI
						TR:I3KHG6_ORENI
Coactivator CBP, KIX domain	IPR003101	407	9	*Danio rerio*	7955	TR:Q1MT59_DANRE
						TR:F1R0I4_DANRE
						TR:A3KQN2_DANRE
				Takifugu rubripes	31033	TR:H2U5B7_TAKRU
						TR:H2U5B6_TAKRU
				Oreochromis niloticus	8128	TR:I3KHG7_ORENI

						TR:I3KHG6_ORENI
						TR:I3KHG7_ORENI
						TR:I3KHG6_ORENI
Nuclear receptor coactivator, interlocking	IPR009110	423	7	*Danio rerio*	7955	TR:Q1MT59_DANRE
						TR:F1R0I4_DANRE
						TR:A3KQN2_DANRE
				Takifugu rubripes	31033	TR:H2U5B7_TAKRU
						TR:H2U5B6_TAKRU
				Oreochromis niloticus	8128	TR:I3KHG7_ORENI
						TR:I3KHG6_ORENI
Nuclear receptor coactivator, CREB-bp-like, interlocking	IPR014744	162	7	*Danio rerio*	7955	TR:Q1MT59_DANRE
						TR:F1R0I4_DANRE
						TR:A3KQN2_DANRE
				Takifugu rubripes	31033	TR:H2U5B7_TAKRU
						TR:H2U5B6_TAKRU
				Oreochromis niloticus	8128	TR:I3KHG7_ORENI
						TR:I3KHG6_ORENI
Zinc finger, TAZ-type	IPR000197	598	10	*Danio rerio*	7955	TR:Q1MT59_DANRE
						TR:F1R0I4_DANRE
						TR:A3KQN2_DANRE
				Takifugu rubripes	31033	TR:H2U5B7_TAKRU
						TR:H2U5B6_TAKRU
				Oreochromis niloticus	8128	TR:I3KHG7_ORENI
						TR:I3KHG6_ORENI
				Caenorhabditis elegans	6239	SP:P34545-3
						SP:P34545-2
						SP:CBP1_CAEEL
Zinc finger, ZZ-type	IPR000433	3365	7	*Danio rerio*	7955	TR:Q1MT59_DANRE
						TR:A3KQN2_DANRE
						TR:F1R0I4_DANRE
				Takifugu rubripes	31033	TR:H2U5B7_TAKRU
						TR:H2U5B6_TAKRU
				Oreochromis niloticus	8128	TR:I3KHG7_ORENI
						TR:I3KHG6_ORENI
Extracellular ligand-binding receptor	IPR001828	7136	2	*Danio rerio*	7955	TR:I3NI77_DANRE
						TR:Q1LUC8_DANRE
Glutamate receptor, L-glutamate/glycine-binding	IPR019594	2097	2	*Danio rerio*	7955	TR:I3NI77_DANRE
						TR:Q1LUC8_DANRE

Ionotropic glutamate receptor	IPR001320	3520	2	*Danio rerio*	7955	TR:I3NI77_DANRE
						TR:Q1LUC8_DANRE
N-Methyl-D-aspartate (NMDA)	IPR001508	1899	2	*Danio rerio*	7955	TR:I3NI77_DANRE
						TR:Q1LUC8_DANRE
Extracellular solute-binding protein, family 3	IPR001638	45324	2	*Danio rerio*	7955	TR:I3NI77_DANRE
						TR:Q1LUC8_DANRE
Shisa family	IPR026910	478	6	*Takifugu rubripes*	31033	TR:H2T9N9_TAKRU
						TR:H2T9N8_TAKRU
						TR:H2T9N4_TAKRU
						TR:H2T9N5_TAKRU
						TR:H2T9N6_TAKRU
						TR:H2T9N7_TAKRU
Bromodomain	IPR001487	7322	10	*Takifugu rubripes*	31033	TR:H2U5B7_TAKRU
						TR:H2U5B6_TAKRU
				Oreochromis niloticus	8128	TR:I3KHG7_ORENI
						TR:I3KHG6_ORENI
				Branchiostoma floridae	7739	TR:C3ZUG6_BRAFL
				Debaryomyces hansenii	284592	TR:Q6BJE1_DEBHA
				Vanderwaltozyma polyspora	436907	TR:A7TQI4_VANPO
				Caenorhabditis elegans	6239	SP:P34545-3
						SP:P34545-2
						SP:CBP1_CAEEL
D-isomer specific 2-hydroxyacid dehydrogenase, catalytic domain	IPR006139	23105	3	*Takifugu rubripes*	31033	TR:H2RUN0_TAKRU
				Tetraodon nigroviridis	99883	TR:H3C4K1_TETNG
						TR:H3C2Q1_TETNG
D-isomer specific 2-hydroxyacid dehydrogenase, NAD-binding	IPR006140	30438	3	*Takifugu rubripes*		TR:H2RUN0_TAKRU
				Tetraodon nigroviridis	99883	TR:H3C4K1_TETNG
						TR:H3C2Q1_TETNG
NAD(P)-binding domain	IPR016040	665366	3	*Takifugu rubripes*	31033	TR:H2RUN0_TAKRU
				Tetraodon nigroviridis	99883	TR:H3C4K1_TETNG
						TR:H3C2Q1_TETNG
Glycine-tyrosine-phenylalanine (GYF)	IPR003169	1085	8	*Homo sapiens*	9606	TR:I1E4Y6_HUMAN
						TR:B9EG55_HUMAN
						TR:E9PBB0_HUMAN
						SP:PERQ2_HUMAN
						SP:Q6Y7W6-3
				Gorilla gorilla gorilla	9595	TR:G3S2D3_GORGO

				Pan troglodytes	9598	TR:H2R5Z9_PANTR
				Drosophila grimshawi	7222	TR:B4JZU4_DROGR
CUB (complement C1r/C1s, Uegf, Bmp1)	IPR000859	6617	1	*Heterocephalus glaber*	10181	TR:G5BHP6_HETGA
frizzled (fz) domain	IPR020067	2130	1	*Heterocephalus glaber*	10181	TR:G5BHP6_HETGA
Low-density lipoprotein (LDL) receptors class A	IPR023415	4529	3	*Heterocephalus glaber*	10181	TR:G5BHP6_HETGA
				Drosophila sechellia	7238	TR:B4HWV4_DROSE
				Drosophila melanogaster	7227	TR:Q9Y110_DROME
Low-density lipoprotein (LDL) receptors class A	IPR002172	6374	3	*Heterocephalus glaber*	10181	TR:G5BHP6_HETGA
				Drosophila sechellia	7238	TR:B4HWV4_DROSE
				Drosophila melanogaster	7227	TR:Q9Y110_DROME
Zinc finger, B-box	IPR000315	9283	2	*Loxodonta africana*	9785	TR:G3TB61_LOXAF
				Oxytricha trifallax	1172189	TR:J9IX07_9SPIT
Zinc finger, RING-type	IPR001841	59461	10	*Loxodonta africana*	9785	TR:G3TB61_LOXAF
				Drosophila virilis	7244	SP:NEUR_DROVI
						TR:B4M452_DROVI
				Drosophila mojavensis	7230	TR:B4KBU2_DROMO
				Drosophila persimilis	7234	TR:B4GI37_DROPE
				Drosophila pseudoobscura pseudoobscura	46245	TR:Q28Z87_DROPS
				Naumovozyma dairenensis	1071378	TR:G0WAM9_NAUDC
				Dictyostelium discoideum	44689	TR:Q75JM9_DICDI
				Paramecium tetraurelia	5888	TR:A0CI00_PARTE
				Tetrahymena thermophila	312017	TR:I7LXF1_TETTS
Zinc finger, RING/FYVE/PHD-type	IPR013083	86396	19	*Loxodonta africana*	9785	TR:G3TB61_LOXAF
				Drosophila mojavensis	7230	TR:B4KBU2_DROMO
				Drosophila virilis	7244	TR:B4LC34_DROVI
						SP:NEUR_DROVI
						TR:B4M452_DROVI
				Drosophila simulans	7240	TR:B4QJJ2_DROSI
				Anopheles gambiae	7165	TR:Q7QET6_ANOGA
						TR:A0ND10_ANOGA
				Drosophila persimilis	7234	TR:B4GI37_DROPE
				Drosophila pseudoobscura pseudoobscura	46245	TR:Q28Z87_DROPS
				Drosophila erecta	7220	TR:B3P3P0_DROER
						TR:B3ND01_DROER
				Drosophila melanogaster	7227	TR:Q9VUB5_DROME
				Candida dubliniensis	573826	TR:B9WA30_CANDC
				Gaeumannomyces graminis var. tritici	644352	TR:J3P975_GAGT3
				Naumovozyma dairenensis	1071378	TR:G0WAM9_NAUDC

				Dictyostelium discoideum	44689	TR:Q75JM9_DICDI
				Oxytricha trifallax	1172189	TR:J9IX07_9SPIT
				Tetrahymena thermophila	312017	TR:I7LXF1_TETTS
Zinc finger, RING-type, conserved site	IPR017907	26140	1	*Loxodonta africana*	9785	TR:G3TB61_LOXAF
Mediator complex, subunit Med15, metazoa	IPR019087	209	3	*Gallus gallus*	9031	TR:F1P1D8_CHICK
				Taeniopygia guttata	59729	TR:H0ZIR6_TAEGU
				Drosophila grimshawi	7222	TR:B4JL62_DROGR
Band 4.1 domain	IPR019749.	4803	1	*Xenopus tropicalis*	8364	TR:F6THY5_XENTR
Band 4.1 family	IPR019750	2356	1	*Xenopus tropicalis*	8364	TR:F6THY5_XENTR
Ezrin/radixin/moesin like	IPR000798	1508	1	*Xenopus tropicalis*	8364	TR:F6THY5_XENTR
FERM adjacent (FA)	IPR014847	1219	1	*Xenopus tropicalis*	8364	TR:F6THY5_XENTR
FERM/acyl-CoA-binding protein, 3-helical bundle	IPR014352	6107	1	*Xenopus tropicalis*	8364	TR:F6THY5_XENTR
FERM central domain	IPR019748	4904	1	*Xenopus tropicalis*	8364	TR:F6THY5_XENTR
FERM conserved site	IPR019747	2213	1	*Xenopus tropicalis*	8364	TR:F6THY5_XENTR
FERM domain	IPR000299.	5158	1	*Xenopus tropicalis*	8364	TR:F6THY5_XENTR
FERM, N-terminal	IPR018979	3230	1	*Xenopus tropicalis*	8364	TR:F6THY5_XENTR
FERM, C-terminal PH-like domain	IPR018980	2555	1	*Xenopus tropicalis*	8364	TR:F6THY5_XENTR
Pleckstrin homology-like domain	IPR011993	42524	4	*Xenopus tropicalis*	8364	TR:F6THY5_XENTR
				Trichosporon asahii var. asahii	1186058	TR:J4UAX5_TRIAS
						TR:K1WQD6_TRIAC
				Trichinella spiralis	6334	TR:E5S526_TRISP
Terminal deoxynucleotidyltransferase-interacting factor 1	PR026064.	78	1	*Crotalus adamanteus*	8729	TR:J3SC40_CROAD
Insect cuticle protein	IPR000618	3690	1	*Antheraea yamamai*	7121	TR:G8FVQ1_ANTYA
NOPS	IPR012975	304	3	*Bombyx mori*	7091	TR:Q0ZAL3_BOMMO
						TR:Q2F5M7_BOMMO
						TR:H9J6N5_BOMMO

Nucleotide-binding, alpha-beta plait	IPR012677	61810	12	*Bombyx mori*	7091	TR:Q0ZAL3_BOMMO
						TR:Q2F5M7_BOMMO
						TR:H9J6N5_BOMMO
				Drosophila persimilis	7234	TR:B4G5G9_DROPE
				Drosophila pseudoobscura pseudoobscura	46245	TR:Q299N5_DROPS
				Picea sitchensis	3332	TR:B8LMP2_PICSI
				Yarrowia lipolytica	284591	TR:Q6CDX5_YARLI
				Nectria haematococca	660122	TR:C7YQJ8_NECH7
				Naumovozyma dairenensis	1071378	TR:G0WAM9_NAUDC
				Torulaspora delbrueckii	1076872	TR:G8ZRC4_TORDC
				Capsaspora owczarzaki	595528	TR:E9C1R9_CAPO3
				Tetrahymena thermophila	312017	TR:I7MB64_TETTS
RNA	IPR000504	48359	12	*Bombyx mori*	7091	TR:Q0ZAL3_BOMMO
						TR:Q2F5M7_BOMMO
						TR:H9J6N5_BOMMO
				Drosophila persimilis	7234	TR:B4G5G9_DROPE
				Drosophila pseudoobscura pseudoobscura	46245	TR:Q299N5_DROPS
				Picea sitchensis	3332	TR:B8LMP2_PICSI
				Yarrowia lipolytica	284591	TR:Q6CDX5_YARLI
				Nectria haematococca	660122	TR:C7YQJ8_NECH7
				Naumovozyma dairenensis	1071378	TR:G0WAM9_NAUDC
				Torulaspora delbrueckii	1076872	TR:G8ZRC4_TORDC
				Capsaspora owczarzaki	595528	TR:E9C1R9_CAPO3
				Tetrahymena thermophila	312017	TR:I7MB64_TETTS
TSC-22 / Dip / Bun	IPR000580	498	1	*Acyrthosiphon pisum*	7029	TR:J9K207_ACYPI
Putative 5-3 exonuclease	IPR004859	947	2	*Acyrthosiphon pisum*	7029	TR:J9JVQ2_ACYPI
				Drosophila mojavensis	7230	TR:B4L6X0_DROMO
Domain of unknown function DUF243	IPR004145	416	1	*Acyrthosiphon pisum*	7029	TR:J9JR44_ACYPI
Transcription factor IIA, helical	IPR009083	789	2	*Acyrthosiphon pisum*	7029	TR:J9K8X1_ACYPI
				Phaeosphaeria nodorum	321614	TR:Q0U9N8_PHANO
Transcription factor IIA, alpha/beta subunit	IPR004855	488	2	*Acyrthosiphon pisum*	7029	TR:J9K8X1_ACYPI
				Phaeosphaeria nodorum	321614	TR:Q0U9N8_PHANO
Transcription factor IIA, alpha subunit, N-terminal	IPR013028	1210	2	*Acyrthosiphon pisum*	7029	TR:J9K8X1_ACYPI
				Phaeosphaeria nodorum	321614	TR:Q0U9N8_PHANO
Transcription factor IIA, beta-barrel	IPR009088	861	2	*Acyrthosiphon pisum*	7029	TR:J9K8X1_ACYPI
				Phaeosphaeria nodorum	321614	TR:Q0U9N8_PHANO

BRK domain	IPR006576	518	1	*Acyrthosiphon pisum*	7029	TR:J9JZG8_ACYPI
Chromo domain	IPR023780	5455	1	*Acyrthosiphon pisum*	7029	TR:J9JZG8_ACYPI
Chromo domain/shadow	IPR000953	6667	2	*Acyrthosiphon pisum*	7029	TR:J9JZG8_ACYPI
				Drosophila mojavensis	7230	TR:B4L5I5_DROMO
Chromo domain-like	IPR016197	7064	2	*Acyrthosiphon pisum*	7029	TR:J9JZG8_ACYPI
				Drosophila mojavensis	7230	TR:B4L5I5_DROMO
Helicase, superfamily 1/2, ATP-binding domain	IPR014001	137284	12	*Acyrthosiphon pisum*	7029	TR:J9JZG8_ACYPI
				Drosophila willistoni	7260	TR:B4NDR4_DROWI
				Arabidopsis thaliana	3702	SP:RH40_ARATH
				Arabidopsis lyrata subsp. lyrata	81972	TR:D7L5I4_ARALL
				Volvox carteri	3067	TR:D8TLQ2_VOLCA
				Debaryomyces hansenii	284592	TR:Q6BJE1_DEBHA
				Ferrimonas balearica	550540	TR:E1SVZ1_FERBD
				Caenorhabditis brenneri	135651	TR:G0M812_CAEBE
				Dictyostelium purpureum	5786	TR:F1A5J1_DICPU
				Dictyostelium discoideum	44689	TR:Q55GK2_DICDI
				Paramecium tetraurelia	5888	TR:A0BWN9_PARTE
				Oxytricha trifallax	1172189	TR:J9HL00_9SPIT
Helicase, C-terminal	IPR001650	130072	10	*Acyrthosiphon pisum*	7029	TR:J9JZG8_ACYPI
				Arabidopsis thaliana	3702	SP:RH40_ARATH
				Arabidopsis lyrata subsp. lyrata	81972	TR:D7L5I4_ARALL
				Volvox carteri	3067	TR:D8TLQ2_VOLCA
				Ferrimonas balearica	550540	TR:E1SVZ1_FERBD
				Caenorhabditis brenneri	135651	TR:G0M812_CAEBE
				Dictyostelium purpureum	5786	TR:F1A5J1_DICPU
				Dictyostelium discoideum	44689	TR:Q55GK2_DICDI
				Paramecium tetraurelia	5888	TR:A0BWN9_PARTE
				Oxytricha trifallax	1172189	TR:J9HL00_9SPIT
SANT/Myb domain	IPR001005	20475	8	*Acyrthosiphon pisum*	7029	TR:J9JZG8_ACYPI
				Vitis vinifera	29760	TR:F6I025_VITVI
				Candida parapsilosis	578454	TR:G8BH70_CANPC
				Cordyceps militaris	983644	TR:G3JEU7_CORMM
				Dictyostelium purpureum	5786	TR:F1A5J1_DICPU
				Dictyostelium discoideum	44689	TR:Q55GK2_DICDI
						SP:MYBU_DICDI
SNF2-related	IPR000330	18770	7	*Acyrthosiphon pisum*	7029	TR:J9JZG8_ACYPI
				Volvox carteri	3067	TR:D8TLQ2_VOLCA
				Debaryomyces hansenii	284592	TR:Q6BJE1_DEBHA

				Caenorhabditis brenneri	135651	TR:G0M812_CAEBE
				Dictyostelium purpureum	5786	TR:F1A5J1_DICPU
				Dictyostelium discoideum	44689	TR:Q55GK2_DICDI
				Oxytricha trifallax	1172189	TR:J9HL00_9SPIT
Protein of unknown function DUF745	IPR007999	193	2	*Drosophila virilis*	7244	TR:B4LE38_DROVI
				Drosophila grimshawi	7222	TR:B4IYI3_DROGR
Endonuclease/exonuclease/phosphatase	IPR005135	31253	2	*Drosophila virilis*	7244	TR:B4MF58_DROVI
				Oxytricha trifallax	1172189	TR:J9ETF9_9SPIT
FAS1 domain	IPR000782	3777	3	*Drosophila virilis*	7244	TR:B4M8R3_DROVI
				Drosophila mojavensis	7230	TR:B4KEJ8_DROMO
				Drosophila grimshawi	7222	TR:B4JD81_DROGR
Ankyrin repeat	IPR002110	54268	6	*Drosophila virilis*	7244	TR:B4M5Q9_DROVI
				Drosophila mojavensis	7230	TR:B4K7G3_DROMO
				Drosophila grimshawi	7222	TR:B4JVH5_DROGR
				Volvox carteri	3067	TR:D8U185_VOLCA
				Polysphondylium pallidum	13642	TR:D3AZ77_POLPA
				Tetrahymena thermophila	312017	TR:Q23QL4_TETTS
Ankyrin repeat-containing domain	IPR020683	57304	6	*Drosophila virilis*	7244	TR:B4M5Q9_DROVI
				Drosophila mojavensis	7230	TR:B4K7G3_DROMO
				Drosophila grimshawi	7222	TR:B4JVH5_DROGR
				Volvox carteri	3067	TR:D8U185_VOLCA
				Polysphondylium pallidum	13642	TR:D3AZ77_POLPA
				Tetrahymena thermophila	312017	TR:Q23QL4_TETTS
K Homology domain	IPR004087	32528	3	*Drosophila virilis*	7244	TR:B4M5Q9_DROVI
				Drosophila mojavensis	7230	TR:B4K7G3_DROMO
				Volvox carteri	3067	TR:D8TYC9_VOLCA
K Homology domain, type 1	IPR004088	23967	3	*Drosophila virilis*	7244	TR:B4M5Q9_DROVI
				Drosophila mojavensis	7230	TR:B4K7G3_DROMO
				Volvox carteri	3067	TR:D8TYC9_VOLCA
C2 calcium-dependent membrane targeting	IPR000008	18264	9	*Drosophila virilis*	7244	TR:B4M6Q2_DROVI
				Drosophila erecta	7220	TR:B3P3P0_DROER
				Volvox carteri	3067	TR:D8U185_VOLCA
				Gaeumannomyces graminis var. tritici	644352	TR:J3P6H6_GAGT3
				Polysphondylium pallidum	13642	TR:D3BU61_POLPA
				Dictyostelium fasciculatum	1054147	TR:F4PU20_DICFS
				Oxytricha trifallax	1172189	TR:J9EJ94_9SPIT
				Naegleria gruberi	5762	TR:D2VCR4_NAEGR

				Trichomonas vaginalis	5722	TR:A2EUT8_TRIVA
C2 calcium/lipid-binding domain, CaLB	IPR008973	20201	9	*Drosophila virilis*	7244	TR:B4M6Q2_DROVI
				Drosophila erecta	7220	TR:B3P3P0_DROER
				Volvox carteri	3067	TR:D8U185_VOLCA
				Gaeumannomyces graminis var. tritici	644352	TR:J3P6H6_GAGT3
				Polysphondylium pallidum	13642	TR:D3BU61_POLPA
				Dictyostelium fasciculatum	1054147	TR:F4PU20_DICFS
				Oxytricha trifallax	1172189	TR:J9EJ94_9SPIT
				Naegleria gruberi	5762	TR:D2VCR4_NAEGR
				Trichomonas vaginalis	5722	TR:A2EUT8_TRIVA
Protein of unknown function DUF3498	IPR021887	276	1	*Drosophila virilis*	7244	TR:B4M6Q2_DROVI
Pleckstrin homology domain	IPR001849	29818	4	*Drosophila virilis*	7244	TR:B4M6Q2_DROVI
				Trichosporon asahii var. asahii	1186058	TR:J4UAX5_TRIAS
				Trichosporon asahii var. asahii	1220162	TR:K1WQD6_TRIAC
				Trichinella spiralis	6334	TR:E5S526_TRISP
Ras GTPase-activating protein	IPR001936	2102	1	*Drosophila virilis*	7244	TR:B4M6Q2_DROVI
Ras GTPase-activating protein, conserved site	IPR023152	1305	1	*Drosophila virilis*	7244	TR:B4M6Q2_DROVI
Rho GTPase activation protein	IPR008936	11228	1	*Drosophila virilis*	7244	TR:B4M6Q2_DROVI
Galactose mutarotase-like domain	IPR011013	31578	1	*Drosophila virilis*	7244	TR:B4M4C9_DROVI
Glycoside hydrolase/deacetylase, beta/alpha-barrel	IPR011330	30252	1	*Drosophila virilis*	7244	TR:B4M4C9_DROVI
Glycosyl hydrolase, family 13, all-beta	IPR013780	27561	1	*Drosophila virilis*	7244	TR:B4M4C9_DROVI
Glycosyl hydrolase family 38, C-terminal	IPR011682	3048	1	*Drosophila virilis*	7244	TR:B4M4C9_DROVI
Glycoside hydrolase, family 38, central domain	IPR015341	3239	1	*Drosophila virilis*	7244	TR:B4M4C9_DROVI
Glycoside hydrolase family 38, N-terminal domain	IPR000602	3430	1	*Drosophila virilis*	7244	TR:B4M4C9_DROVI
Cysteine-rich flanking region, C-terminal	IPR000483	7421	12	*Drosophila virilis*	7244	TR:B4LQ18_DROVI
				Drosophila mojavensis	7230	TR:B4KT45_DROMO
				Drosophila ananassae	7217	TR:B3MJR7_DROAN
						TR:B3MEY9_DROAN
				Drosophila sechellia	7238	TR:B4HSK9_DROSE
				Drosophila simulans	7240	TR:B4QHG3_DROSI

				Drosophila yakuba	7245	TR:B4P6Q2_DROYA
				Drosophila erecta	7220	TR:B3NPW3_DROER
				Drosophila melanogaster	7227	TR:A1ZAB1_DROME
						TR:C4IXX4_DROME
						TR:C4IXX5_DROME
				Drosophila grimshawi	7222	TR:B4JWA4_DROGR
Immunoglobulin-like domain	IPR007110	63124	13	*Drosophila virilis*	7244	TR:B4LQ18_DROVI
				Drosophila mojavensis	7230	TR:B4KT45_DROMO
				Drosophila ananassae	7217	TR:B3MJR7_DROAN
						TR:B3MEY9_DROAN
				Drosophila sechellia	7238	TR:B4HSK9_DROSE
				Drosophila simulans	7240	TR:B4QHG3_DROSI
				Drosophila yakuba	7245	TR:B4P6Q2_DROYA
				Drosophila erecta	7220	TR:B3NPW3_DROER
				Drosophila melanogaster	7227	TR:A1ZAB1_DROME
						TR:C4IXX4_DROME
				Drosophila grimshawi	7222	TR:B4JWA4_DROGR
				Pristionchus pacificus	54126	TR:H3FTN9_PRIPA
				Ascaris suum	6253	TR:F1KQG0_ASCSU
Immunoglobulin-like fold	IPR013783	128338	16	*Drosophila virilis*	7244	TR:B4LQ18_DROVI
				Drosophila mojavensis	7230	TR:B4KT45_DROMO
				Drosophila ananassae	7217	TR:B3MJR7_DROAN
						TR:B3MEY9_DROAN
				Drosophila sechellia	7238	TR:B4HSK9_DROSE
				Drosophila simulans	7240	TR:B4QHG3_DROSI
				Drosophila yakuba	7245	TR:B4P6Q2_DROYA
				Drosophila erecta	7220	TR:B3NPW3_DROER
				Drosophila melanogaster	7227	TR:A1ZAB1_DROME
						TR:C4IXX4_DROME
				Drosophila grimshawi	7222	TR:B4JWA4_DROGR
						TR:B4JD22_DROGR
				Pristionchus pacificus	54126	TR:H3FTN9_PRIPA
				Ascaris suum	6253	TR:F1KQG0_ASCSU
				Kitasatospora setae	452652	TR:E4N1V7_KITSK
				Dictyostelium purpureum	5786	TR:F0ZYQ8_DICPU
Immunoglobulin I-set	IPR013098	19132	13	*Drosophila virilis*	7244	TR:B4LQ18_DROVI
				Drosophila mojavensis	7230	TR:B4KT45_DROMO
				Drosophila ananassae	7217	TR:B3MJR7_DROAN
						TR:B3MEY9_DROAN
				Drosophila sechellia	7238	TR:B4HSK9_DROSE
				Drosophila simulans	7240	TR:B4QHG3_DROSI
				Drosophila yakuba	7245	TR:B4P6Q2_DROYA

				Drosophila erecta	7220	TR:B3NPW3_DROER
				Drosophila melanogaster	7227	TR:A1ZAB1_DROME
						TR:C4IXX4_DROME
				Drosophila grimshawi	7222	TR:B4JWA4_DROGR
				Pristionchus pacificus	54126	TR:H3FTN9_PRIPA
				Ascaris suum	6253	TR:F1KQG0_ASCSU
Immunoglobulin subtype	IPR003599	28488	12	*Drosophila virilis*	7244	TR:B4LQ18_DROVI
				Drosophila mojavensis	7230	TR:B4KT45_DROMO
				Drosophila ananassae	7217	TR:B3MEY9_DROAN
				Drosophila sechellia	7238	TR:B4HSK9_DROSE
				Drosophila simulans	7240	TR:B4QHG3_DROSI
				Drosophila yakuba	7245	TR:B4P6Q2_DROYA
				Drosophila erecta	7220	TR:B3NPW3_DROER
				Drosophila melanogaster	7227	TR:A1ZAB1_DROME
						TR:C4IXX4_DROME
				Drosophila grimshawi	7222	TR:B4JWA4_DROGR
				Pristionchus pacificus	54126	TR:H3FTN9_PRIPA
				Ascaris suum	6253	TR:F1KQG0_ASCSU
Immunoglobulin subtype 2	IPR003598	19241	12	*Drosophila virilis*	7244	TR:B4LQ18_DROVI
				Drosophila mojavensis	7230	TR:B4KT45_DROMO
				Drosophila ananassae	7217	TR:B3MJR7_DROAN
						TR:B3MEY9_DROAN
				Drosophila sechellia	7238	TR:B4HSK9_DROSE
				Drosophila simulans	7240	TR:B4QHG3_DROSI
				Drosophila yakuba	7245	TR:B4P6Q2_DROYA
				Drosophila melanogaster	7227	TR:A1ZAB1_DROME
						TR:C4IXX4_DROME
				Drosophila grimshawi	7222	TR:B4JWA4_DROGR
				Pristionchus pacificus	54126	TR:H3FTN9_PRIPA
				Ascaris suum	6253	TR:F1KQG0_ASCSU
Leucine	IPR001611	42876	16	*Drosophila virilis*	7244	TR:B4LQ18_DROVI
				Drosophila mojavensis	7230	TR:B4KT45_DROMO
				Drosophila ananassae	7217	TR:B3MJR7_DROAN
						TR:B3MEY9_DROAN
				Drosophila sechellia	7238	TR:B4HSK9_DROSE
						TR:B4HTZ7_DROSE
				Drosophila simulans	7240	TR:B4QHG3_DROSI
				Drosophila yakuba	7245	TR:B4PHP3_DROYA
						TR:B4P6Q2_DROYA
				Drosophila erecta	7220	TR:B3NG33_DROER
						TR:B3NPW3_DROER
				Drosophila melanogaster	7227	TR:A1ZAB1_DROME

						TR:C4IXX4_DROME
						TR:C4IXX5_DROME
				Drosophila grimshawi	7222	TR:B4JWA4_DROGR
				Yarrowia lipolytica	284591	TR:Q6C1M0_YARLI
Leucine-rich repeat, typical subtype	IPR003591	16374	12	*Drosophila virilis*	7244	TR:B4LQ18_DROVI
				Drosophila mojavensis	7230	TR:B4KT45_DROMO
				Drosophila ananassae	7217	TR:B3MJR7_DROAN
						TR:B3MJR7_DROAN
						TR:B3MEY9_DROAN
				Drosophila sechellia	7238	TR:B4HSK9_DROSE
				Drosophila simulans	7240	TR:B4QHG3_DROSI
				Drosophila erecta	7220	TR:B3NPW3_DROER
				Drosophila melanogaster	7227	TR:A1ZAB1_DROME
						TR:C4IXX4_DROME
						TR:C4IXX5_DROME
				Drosophila grimshawi	7222	TR:B4JWA4_DROGR
Leucine-rich repeat-containing N-terminal	IPR000372	8238	11	*Drosophila virilis*	7244	TR:B4LQ18_DROVI
				Drosophila mojavensis	7230	TR:B4KT45_DROMO
				Drosophila ananassae	7217	TR:B3MEY9_DROAN
				Drosophila sechellia	7238	TR:B4HSK9_DROSE
				Drosophila simulans	7240	TR:B4QHG3_DROSI
				Drosophila yakuba	7245	TR:B4P6Q2_DROYA
				Drosophila erecta	7220	TR:B3NPW3_DROER
				Drosophila melanogaster	7227	TR:A1ZAB1_DROME
						TR:C4IXX4_DROME
						TR:C4IXX5_DROME
				Drosophila grimshawi	7222	TR:B4JWA4_DROGR
BRCT domain	IPR001357	13569	7	*Drosophila virilis*	7244	TR:B4LC32_DROVI
				Drosophila mojavensis	7230	TR:B4L165_DROMO
				Drosophila sechellia	7238	TR:B4HHB0_DROSE
				Drosophila simulans	7240	TR:B4QJJ4_DROSI
				Drosophila willistoni	7260	TR:B4MLV6_DROWI
				Drosophila melanogaster	7227	TR:Q9VUB7_DROME
				Drosophila grimshawi	7222	TR:B4IXZ0_DROGR
NEUZ is a domain of unknown function found in neuralized proteins	IPR006573	500	3	*Drosophila virilis*	7244	SP:NEUR_DROVI
				Drosophila mojavensis	7230	TR:B4KBU2_DROMO
				Drosophila virilis	7244	TR:B4M452_DROVI
Zinc finger, C2H2	IPR007087	72394	19	*Drosophila virilis*	7244	SP:NEUR_DROVI
						TR:B4M452_DROVI

				Drosophila mojavensis	7230	TR:B4KUQ6_DROMO
						TR:B4L1S9_DROMO
						TR:B4KIA8_DROMO
				Drosophila willistoni	7260	TR:B4N1N6_DROWI
						TR:B4MIR0_DROWI
				Anopheles gambiae	7165	TR:Q7QET6_ANOGA
						TR:A0ND10_ANOGA
				Tetraodon nigroviridis	99883	TR:Q4RUC4_TETNG
				Yarrowia lipolytica	284591	TR:Q6CGR7_YARLI
				Candida dubliniensis	573826	TR:B9WEZ3_CANDC
				Spathaspora passalidarum	619300	TR:G3AT45_SPAPN
				Macrophomina phaseolina	1126212	TR:K2S7S7_MACPH
				Vanderwaltozyma polyspora	436907	TR:A7TRZ6_VANPO
				Caenorhabditis remanei	31234	TR:E3LN59_CAERE
				Dictyostelium discoideum	44689	TR:Q556F5_DICDI
				Monosiga brevicollis	81824	TR:A9V3U7_MONBE
				Salpingoeca rosetta	946362	TR:F2TZX2_SALS5
Zinc finger, C2H2-like	IPR015880	82683	21	*Drosophila virilis*	7244	SP:NEUR_DROVI
						TR:B4M452_DROVI
				Culex quinquefasciatus	7176	TR:B0W767_CULQU
				Drosophila mojavensis	7230	TR:B4KUQ6_DROMO
						TR:B4L1S9_DROMO
						TR:B4KIA8_DROMO
				Drosophila willistoni	7260	TR:B4N1N6_DROWI
				Anopheles gambiae	7165	TR:Q7QET6_ANOGA
						TR:A0ND10_ANOGA
				Tetraodon nigroviridis	99883	TR:Q4RUC4_TETNG
				Yarrowia lipolytica	284591	TR:Q6CGR7_YARLI
				Candida dubliniensis	573826	TR:B9WEZ3_CANDC
				Spathaspora passalidarum	619300	TR:G3AT45_SPAPN
				Macrophomina phaseolina	1126212	TR:K2S5I6_MACPH
						TR:K2S7S7_MACPH
				Vanderwaltozyma polyspora	436907	TR:A7TRZ6_VANPO
				Caenorhabditis remanei	31234	TR:E3LN59_CAERE
				Dictyostelium discoideum	44689	TR:Q556F5_DICDI
				Tetrahymena thermophila	312017	TR:I7MB64_TETTS
				Monosiga brevicollis	81824	TR:A9V3U7_MONBE
				Salpingoeca rosetta	946362	TR:F2TZX2_SALS5
Zinc finger C2H2-type/integrase DNA-binding domain	IPR013087	63783	13	*Drosophila virilis*	7244	TR:B4LV56_DROVI
				Drosophila mojavensis	7230	TR:B4KUQ6_DROMO
						TR:B4L1S9_DROMO
				Drosophila willistoni	7260	TR:B4N1N6_DROWI
				Anopheles gambiae	7165	TR:Q7QET6_ANOGA

						TR:A0ND10_ANOGA
				Yarrowia lipolytica	284591	TR:Q6CGR7_YARLI
				Candida dubliniensis	573826	TR:B9WEZ3_CANDC
				Spathaspora passalidarum	619300	TR:G3AT45_SPAPN
				Vanderwaltozyma polyspora	436907	TR:A7TRZ6_VANPO
				Caenorhabditis remanei	31234	TR:E3LN59_CAERE
				Monosiga brevicollis	81824	TR:A9V3U7_MONBE
				Salpingoeca rosetta	946362	TR:F2TZX2_SALS5
Nuclear hormone receptor, ligand-binding	IPR008946	8956	4	*Drosophila virilis*	7244	TR:B4LHV8_DROVI
				Drosophila grimshawi	7222	TR:B4J0N8_DROGR
				Drosophila mojavensis	7230	TR:B4L034_DROMO
				Aedes aegypti	7159	TR:Q0IE91_AEDAE
Zinc finger, nuclear hormone receptor-type	IPR001628.	7972	4	*Drosophila virilis*	7244	TR:B4LHV8_DROVI
				Drosophila mojavensis	7230	TR:B4L034_DROMO
				Drosophila grimshawi	7222	TR:B4J0N8_DROGR
				Aedes aegypti	7159	TR:Q0IE91_AEDAE
Homeobox protein, antennapedia type, conserved site	IPR001827	1969	13	*Drosophila virilis*	7244	TR:Q8I195_DROVI
				Drosophila mojavensis	7230	TR:B4K6G4_DROMO
				Drosophila sechellia	7238	TR:B4I4J9_DROSE
				Drosophila simulans	7240	TR:B4QYW7_DROSI
				Drosophila persimilis	7234	TR:B4GEM5_DROPE
				Drosophila pseudoobscura pseudoobscura	46245	TR:Q296U8_DROPS
				Drosophila yakuba	7245	TR:B4PSJ0_DROYA
				Drosophila erecta	7220	TR:B3P2P9_DROER
				Drosophila melanogaster	7227	SP:HMPB_DROME
				Drosophila grimshawi	7222	TR:B4JGM0_DROGR
				Drosophila melanogaster	7227	SP:P31264-2
						SP:P31264-3
						SP:P31264-4
UBA-like	IPR009060	16268	5	*Drosophila virilis*	7244	TR:B4M1G9_DROVI
				Rhizopus delemar	246409	TR:I1BLN6_RHIO9
						TR:I1BI10_RHIO9
				Phaeosphaeria nodorum	321614	TR:Q0UXI8_PHANO
				Ichthyophthirius multifiliis	857967	TR:G0R1R9_ICHMG
Armadillo-like helical	IPR011989	58193	11	*Drosophila virilis*	7244	TR:B4LHR0_DROVI
				Drosophila grimshawi	7222	TR:B4JNB2_DROGR
				Anopheles gambiae	7165	TR:A7UVK4_ANOGA
						TR:Q7PNN3_ANOGA
				Arthroderma benhamiae	663331	TR:D4AIT9_ARTBC
				Trichophyton verrucosum	663202	TR:D4DFR8_TRIVH

				Nectria haematococca	660122	TR:C7YQJ8_NECH7
				Loa loa	7209	TR:J0DW06_LOALO
				Dictyostelium purpureum	5786	TR:F0ZS77_DICPU
				Paramecium tetraurelia	5888	TR:A0CBT3_PARTE
				Oxytricha trifallax	1172189	TR:J9I6T8_9SPIT
Armadillo -type fold	IPR016024	77988	7	*Drosophila virilis*	7244	TR:B4LHR0_DROVI
				Drosophila persimilis	7234	TR:B4G502_DROPE
				Drosophila pseudoobscura pseudoobscura	46245	TR:B5DYF9_DROPS
				Rhizopus delemar	246409	TR:I1CEW0_RHIO9
				Dictyostelium purpureum	5786	TR:F0ZS77_DICPU
				Paramecium tetraurelia	5888	TR:A0CBT3_PARTE
				Oxytricha trifallax	1172189	TR:J9I6T8_9SPIT
Cell morphogenesis protein N-terminal	IPR025614	418	1	*Drosophila virilis*	7244	TR:B4LHR0_DROVI
Cell morphogenesis protein C-terminal	IPR025481	410	1	*Drosophila virilis*	7244	TR:B4LHR0_DROVI
SET domain	IPR001214	11343	5	*Drosophila virilis*	7244	TR:B4LC34_DROVI
				Drosophila simulans	7240	TR:B4QJJ2_DROSI
				Drosophila erecta	7220	TR:B3ND01_DROER
				Drosophila melanogaster	7227	TR:Q9VUB5_DROME
				Candida dubliniensis	573826	TR:B9WA30_CANDC
Zinc finger, PHD-type, conserved site	IPR019786	10490	6	*Drosophila virilis*	7244	TR:B4LC34_DROVI
				Drosophila simulans	7240	TR:B4QJJ2_DROSI
				Drosophila erecta	7220	TR:B3ND01_DROER
				Drosophila melanogaster	7227	TR:Q9VUB5_DROME
				Candida dubliniensis	573826	TR:B9WA30_CANDC
				Gaeumannomyces graminis var. tritici	644352	TR:J3P975_GAGT3
Zinc finger, FYVE/PHD-type	IPR011011	20769	9	*Drosophila virilis*	7244	TR:B4LC34_DROVI
				Drosophila simulans	7240	TR:B4QJJ2_DROSI
				Drosophila erecta	7220	TR:B3P3P0_DROER
						TR:B3ND01_DROER
				Drosophila melanogaster	7227	TR:Q9VUB5_DROME
				Anopheles gambiae	7165	TR:Q7QET6_ANOGA
						TR:A0ND10_ANOGA
				Candida dubliniensis	573826	TR:B9WA30_CANDC
				Gaeumannomyces graminis var. tritici	644352	TR:J3P975_GAGT3
Zinc finger, PHD-type	IPR001965	14838	7	*Drosophila virilis*	7244	TR:B4LC34_DROVI
				Drosophila simulans	7240	TR:B4QJJ2_DROSI
				Drosophila erecta	7220	TR:B3ND01_DROER
				Anopheles gambiae	7165	TR:Q7QET6_ANOGA

						TR:A0ND10_ANOGA
				Drosophila melanogaster	7227	TR:Q9VUB5_DROME
				Gaeumannomyces graminis var. tritici	644352	TR:J3P975_GAGT3
Zinc finger, PHD-finger	IPR019787	11882	8	*Drosophila virilis*	7244	TR:B4LC34_DROVI
				Drosophila simulans	7240	TR:B4QJJ2_DROSI
				Drosophila erecta	7220	TR:B3ND01_DROER
				Anopheles gambiae	7165	TR:Q7QET6_ANOGA
						TR:A0ND10_ANOGA
				Drosophila melanogaster	7227	TR:Q9VUB5_DROME
				Candida dubliniensis	573826	TR:B9WA30_CANDC
				Gaeumannomyces graminis var. tritici	644352	TR:J3P975_GAGT3
Sec23/Sec24, helical domain	IPR006900	1663	7	*Drosophila virilis*	7244	TR:B4LL65_DROVI
				Drosophila mojavensis	7230	TR:B4KQA1_DROMO
				Drosophila sechellia	7238	TR:B4I2G8_DROSE
				Drosophila yakuba	7245	TR:B4NW71_DROYA
				Drosophila erecta	7220	TR:B3N9H8_DROER
				Drosophila melanogaster	7227	TR:Q9VQ94_DROME
				Paramecium tetraurelia	5888	TR:A0DPT5_PARTE
Sec23/Sec24, trunk domain	IPR006896	1755	7	*Drosophila virilis*	7244	TR:B4LL65_DROVI
				Drosophila mojavensis	7230	TR:B4KQA1_DROMO
				Drosophila sechellia	7238	TR:B4I2G8_DROSE
				Drosophila yakuba	7245	TR:B4NW71_DROYA
				Drosophila erecta	7220	TR:B3N9H8_DROER
				Drosophila melanogaster	7227	TR:Q9VQ94_DROME
				Paramecium tetraurelia	5888	TR:A0DPT5_PARTE
Sec23/Sec24 beta-sandwich	IPR012990	1658	7	*Drosophila virilis*	7244	TR:B4LL65_DROVI
				Drosophila mojavensis	7230	TR:B4KQA1_DROMO
				Drosophila sechellia	7238	TR:B4I2G8_DROSE
				Drosophila yakuba	7245	TR:B4NW71_DROYA
				Drosophila erecta	7220	TR:B3N9H8_DROER
				Drosophila melanogaster	7227	TR:Q9VQ94_DROME
				Paramecium tetraurelia	5888	TR:A0DPT5_PARTE
Zinc finger, Sec23/Sec24-type	IPR006895	1711	7	*Drosophila virilis*	7244	TR:B4LL65_DROVI
				Drosophila mojavensis	7230	TR:B4KQA1_DROMO
				Drosophila sechellia	7238	TR:B4I2G8_DROSE
				Drosophila yakuba	7245	TR:B4NW71_DROYA
				Drosophila erecta	7220	TR:B3N9H8_DROER
				Drosophila melanogaster	7227	TR:Q9VQ94_DROME
				Paramecium tetraurelia	5888	TR:A0DPT5_PARTE

Gelsolin domain	IPR007123	2644	6	*Drosophila mojavensis*	7230	TR:B4KQA1_DROMO
				Drosophila sechellia	7238	TR:B4I2G8_DROSE
				Drosophila yakuba	7245	TR:B4NW71_DROYA
				Drosophila erecta	7220	TR:B3N9H8_DROER
				Drosophila melanogaster	7227	TR:Q9VQ94_DROME
				Paramecium tetraurelia	5888	TR:A0DPT5_PARTE
Zinc finger, C2CH-type	IPR006612	1885	1	*Drosophila mojavensis*	7230	TR:B4KIA8_DROMO
Zinc finger, double-stranded RNA binding	IPR022755	2427	2	*Drosophila mojavensis*	7230	TR:B4L1S9_DROMO
				Drosophila willistoni	7260	TR:B4N1N6_DROWI
Nuclear hormone receptor, ligand-binding, core	IPR000536	7988	3	*Drosophila mojavensis*	7230	TR:B4L034_DROMO
				Drosophila grimshawi	7222	TR:B4J0N8_DROGR
				Aedes aegypti	7159	TR:Q0IE91_AEDAE
Steroid hormone receptor	IPR001723	6406	3	*Drosophila mojavensis*	7230	TR:B4L034_DROMO
				Drosophila grimshawi	7222	TR:B4J0N8_DROGR
				Aedes aegypti	7159	TR:Q0IE91_AEDAE
Zinc finger, NHR/GATA-type	IPR013088	13177	6	*Drosophila mojavensis*	7230	TR:B4L034_DROMO
				Drosophila grimshawi	7222	TR:B4J0N8_DROGR
				Aedes aegypti	7159	TR:Q0IE91_AEDAE
				Rhizopus delemar	246409	TR:I1CHV5_RHIO9
				Dictyostelium discoideum	44689	SP:GTAG_DICDI
				Salpingoeca rosetta	946362	TR:F2TY54_SALS5
Leucine rich repeat 5	IPR026906	5070	1	*Drosophila mojavensis*	7230	TR:B4KT45_DROMO
Polyadenylate-binding protein/Hyperplastic disc protein	IPR002004	1618	2	*Drosophila mojavensis*	7230	TR:B4L3F3_DROMO
				Capsaspora owczarzaki	595528	TR:E9C1R9_CAPO3
Chitin binding domain	IPR002557	3833	3	*Drosophila mojavensis*	7230	TR:B4KNT1_DROMO
				Anopheles gambiae	7165	TR:A7UVK4_ANOGA
						TR:Q7PNN3_ANOGA
High mobility group (HMG) box domain	IPR009071	10524	3	*Drosophila mojavensis*	7230	TR:B4KRC4_DROMO
				Drosophila ananassae	7217	TR:B3MM06_DROAN
				Komagataella pastoris	981350	TR:F2QM85_PICP7
Homeobox, conserved site	IPR017970	20442	20	*Drosophila mojavensis*	7230	TR:B4K845_DROMO
						TR:B4K6G4_DROMO
				Drosophila ananassae	7217	TR:B3M2C6_DROAN
				Drosophila sechellia	7238	TR:B4I4J9_DROSE
						TR:B4IBQ1_DROSE

				Drosophila simulans	7240	TR:B4QYW7_DROSI
						TR:B4QWE8_DROSI
				Drosophila persimilis	7234	TR:B4GEM5_DROPE
				Drosophila pseudoobscura pseudoobscura	46245	TR:Q296U8_DROPS
				Drosophila yakuba	7245	TR:B4PSJ0_DROYA
						TR:B4PMW0_DROYA
				Drosophila erecta	7220	TR:B3P2P9_DROER
						TR:B3NZB3_DROER
				Drosophila grimshawi	7222	TR:B4JGM0_DROGR
						TR:B4JX14_DROGR
				Drosophila melanogaster	7227	SP:ABDB_DROME
						SP:HMPB_DROME
						SP:P31264-2
						SP:P31264-3
						SP:P31264-4
Homeodomain, metazoa	IPR020479	9494	19	*Drosophila mojavensis*	7230	TR:B4K845_DROMO
						TR:B4K6G4_DROMO
				Drosophila ananassae	7217	TR:B3M2C6_DROAN
				Drosophila sechellia	7238	TR:B4I4J9_DROSE
						TR:B4IBQ1_DROSE
				Drosophila simulans	7240	TR:B4QYW7_DROSI
						TR:B4QWE8_DROSI
				Drosophila persimilis	7234	TR:B4GEM5_DROPE
				Drosophila pseudoobscura pseudoobscura	46245	TR:Q296U8_DROPS
				Drosophila yakuba	7245	TR:B4PSJ0_DROYA
						TR:B4PMW0_DROYA
				Drosophila erecta	7220	TR:B3P2P9_DROER
						TR:B3NZB3_DROER
				Drosophila grimshawi	7222	TR:B4JGM0_DROGR
				Drosophila melanogaster	7227	SP:ABDB_DROME
						SP:HMPB_DROME
						SP:P31264-2
						SP:P31264-3
						SP:P31264-4
Homeodomain	IPR001356	30961	20	*Drosophila mojavensis*	7230	TR:B4K845_DROMO
						TR:B4K6G4_DROMO
				Drosophila ananassae	7217	TR:B3M2C6_DROAN
				Drosophila sechellia	7238	TR:B4I4J9_DROSE
						TR:B4IBQ1_DROSE
				Drosophila simulans	7240	TR:B4QYW7_DROSI
						TR:B4QWE8_DROSI
				Drosophila persimilis	7234	TR:B4GEM5_DROPE
				Drosophila pseudoobscura pseudoobscura	46245	TR:Q296U8_DROPS

				Drosophila yakuba	7245	TR:B4PSJ0_DROYA
						TR:B4PMW0_DROYA
				Drosophila erecta	7220	TR:B3P2P9_DROER
						TR:B3NZB3_DROER
				Drosophila grimshawi	7222	TR:B4JGM0_DROGR
						TR:B4JX14_DROGR
				Drosophila melanogaster	7227	SP:ABDB_DROME
						SP:HMPB_DROME
						SP:P31264-2
						SP:P31264-3
						SP:P31264-4
Homeodomain-like	IPR009057	322630	26	*Drosophila mojavensis*	7230	TR:B4K845_DROMO
						TR:B4K6G4_DROMO
				Drosophila ananassae	7217	TR:B3M2C6_DROAN
				Drosophila sechellia	7238	TR:B4I4J9_DROSE
						TR:B4IBQ1_DROSE
				Drosophila simulans	7240	TR:B4QYW7_DROSI
						TR:B4QWE8_DROSI
				Drosophila persimilis	7234	TR:B4GEM5_DROPE
				Drosophila yakuba	7245	TR:B4PSJ0_DROYA
						TR:B4PMW0_DROYA
				Drosophila erecta	7220	TR:B3P2P9_DROER
						TR:B3NZB3_DROER
				Drosophila grimshawi	7222	TR:B4JGM0_DROGR
						TR:B4JX14_DROGR
				Drosophila melanogaster	7227	SP:ABDB_DROME
						SP:HMPB_DROME
						SP:P31264-2
						SP:P31264-3
						SP:P31264-4
				Dictyostelium discoideum	44689	SP:MYBU_DICDI
				Lodderomyces elongisporus	379508	TR:A5E1L5_LODEL
				Candida parapsilosis	578454	TR:G8BH70_CANPC
				Cordyceps militaris	983644	TR:G3JEU7_CORMM
				Vitis vinifera	29760	TR:F6I025_VITVI
				Dictyostelium purpureum	5786	TR:F1A5J1_DICPU
				Dictyostelium discoideum	44689	TR:Q55GK2_DICDI
Nebulin 35 residue motif	IPR000900	520	3	*Drosophila mojavensis*	7230	TR:B4KXK5_DROMO
				Drosophila pseudoobscura pseudoobscura	46245	TR:Q29DT1_DROPS
						TR:Q296U8_DROPS
Src homology-3 domain	IPR001452	24640	11	*Drosophila mojavensis*	7230	TR:B4KXK5_DROMO
				Drosophila sechellia	7238	TR:B4ID22_DROSE

				Drosophila simulans	7240	TR:B4Q6K2_DROSI
				Drosophila pseudoobscura pseudoobscura	46245	TR:Q29DT1_DROPS
				Drosophila willistoni	7260	TR:B4NBV6_DROWI
				Drosophila erecta	7220	TR:B3N892_DROER
				Drosophila melanogaster	7227	TR:Q9VPU1_DROME
				Drosophila grimshawi	7222	TR:B4JVH5_DROGR
				Lachancea thermotolerans	559295	TR:C5DLP2_LACTC
				Colletotrichum graminicola	645133	TR:E3QDI8_COLGM
				Kluyveromyces lactis	284590	TR:Q6CPM1_KLULA
Zinc finger, LIM-type	IPR001781	8426	3	*Drosophila mojavensis*	7230	TR:B4KXK5_DROMO
				Drosophila pseudoobscura pseudoobscura	46245	TR:Q29DT1_DROPS
				Scheffersomyces stipitis	322104	TR:A3LYJ2_PICST
RNA binding activity-knot of a chromodomain	IPR025995	1322	1	*Drosophila mojavensis*	7230	TR:B4L5I5_DROMO
MADF domain	IPR006578	1553	2	*Drosophila mojavensis*	7230	TR:B4KR77_DROMO
				Drosophila willistoni	7260	TR:B4MYB5_DROWI
Homeobox protein, antennapedia type	IPR017995	1086	4	*Drosophila mojavensis*	7230	TR:B4K6G4_DROMO
				Drosophila persimilis	7234	TR:B4GEM5_DROPE
				Drosophila pseudoobscura pseudoobscura	46245	TR:Q296U8_DROPS
				Drosophila grimshawi	7222	TR:B4JGM0_DROGR
Myc-type, basic helix-loop-helix (bHLH) domain	IPR011598	17829	2	*Drosophila mojavensis*	7230	TR:B4K809_DROMO
				Drosophila sechellia	7238	TR:B4IFN2_DROSE
Translation Initiation factor eIF- 4e-like domain	IPR023398	1969	1	*Drosophila mojavensis*	7230	TR:B4KW75_DROMO
Translation Initiation factor eIF- 4e	IPR001040	1780	1	*Drosophila mojavensis*	7230	TR:B4KW75_DROMO
Eukaryotic translation initiation factor 4E (eIF-4E), conserved site	IPR019770	976	1	*Drosophila mojavensis*	7230	TR:B4KW75_DROMO
GRASP55/65 PDZ-like domain (Golgi reassembly stacking protein of 55/65 kDa)	IPR024958	473	1	*Culex quinquefasciatus*	7176	TR:B0XBV1_CULQU
GRASP55 (Golgi reassembly stacking protein of 55 kDa) and GRASP65 (a 65 kDa)	IPR007583	444	1	*Culex quinquefasciatus*	7176	TR:B0XBV1_CULQU
PDZ domain	IPR001478	48125	5	*Culex quinquefasciatus*	7176	TR:B0XBV1_CULQU
				Drosophila simulans	7240	TR:B4QFM5_DROSI
				Drosophila willistoni	7260	TR:B4NBV6_DROWI
						TR:B4MKD0_DROWI
				Drosophila grimshawi	7222	TR:B4JVH5_DROGR

ALIX V-shaped domain	IPR025304	680	4	*Culex quinquefasciatus*	7176	TR:B0WWP0_CULQU
				Paramecium tetraurelia	5888	TR:A0CDE8_PARTE
						TR:A0DFK8_PARTE
				Leishmania braziliensis	5660	TR:A4HFU0_LEIBR
BRO1 domain	IPR004328	1272	4	*Culex quinquefasciatus*	7176	TR:B0WWP0_CULQU
				Paramecium tetraurelia	5888	TR:A0CDE8_PARTE
						TR:A0DFK8_PARTE
				Leishmania braziliensis	5660	TR:A4HFU0_LEIBR
Protein-tyrosine phosphatase, receptor/non-receptor type	IPR000242	6714	1	*Culex quinquefasciatus*	7176	TR:B0WWP0_CULQU
Peptidase C48, SUMO/Sentrin/ Ubl1	IPR003653	3905	2	*Anopheles gambiae*	7165	TR:A7UVK4_ANOGA
						TR:Q7PNN3_ANOGA
Acyl-CoA N-acyltransferase	IPR016181	182379	2	*Anopheles gambiae*	7165	TR:A0ND10_ANOGA
						TR:Q7QET6_ANOGA
Histone H1/H5	IPR005818	2159	2	*Anopheles gambiae*	7165	TR:A0ND10_ANOGA
						TR:Q7QET6_ANOGA
MOZ/SAS-like protein	IPR002717	1432	2	*Anopheles gambiae*	7165	TR:A0ND10_ANOGA
						TR:Q7QET6_ANOGA
Mediator complex, subunit Med21	IPR021384	320	1	*Anopheles gambiae*	7165	SP:MED21_ANOGA
DnaJ domain	IPR001623	38316	1	*Aedes aegypti*	7159	TR:Q17E65_AEDAE
	IPR003095		1	*Aedes aegypti*	7159	TR:Q17E65_AEDAE
EF-hand-like domain	IPR011992.	45075	7	*Volvox carteri*	3067	TR:D8U185_VOLCA
				Apis mellifera	7460	TR:H9K2L4_APIME
				Branchiostoma floridae	7739	TR:C3ZBA3_BRAFL
				Emericella nidulans	227321	TR:Q5B077_EMENI
				Dictyostelium fasciculatum	1054147	TR:F4Q4C0_DICFS
				Paramecium tetraurelia	5888	TR:A0BWN9_PARTE
				Phaeodactylum tricornutum	556484	TR:B7FRA9_PHATC
EF-Hand 1, calcium-binding site	IPR018247	73783	4	*Volvox carteri*	3067	TR:D8U185_VOLCA
				Apis mellifera	7460	TR:H9K2L4_APIME
				Emericella nidulans	227321	TR:Q5B077_EMENI
				Paramecium tetraurelia	5888	TR:A0BWN9_PARTE

Ribosomal protein L14 domain	IPR023571	7979	1	*Drosophila ananassae*	7217	TR:B3MYV1_DROAN
Ribosomal protein L14b/L23e	IPR000218	7984	1	*Drosophila ananassae*	7217	TR:B3MYV1_DROAN
Genetic suppressor element-like	IPR022207	123	12	*Drosophila ananassae*	7217	TR:B3M3P4_DROAN
				Drosophila sechellia	7238	TR:B4HUG6_DROSE
				Drosophila simulans	7240	TR:B4QRL6_DROSI
				Drosophila persimilis	7234	TR:B4H3D3_DROPE
				Drosophila pseudoobscura pseudoobscura	46245	TR:Q2LZM8_DROPS
				Drosophila yakuba	7245	TR:B4PIU8_DROYA
				Drosophila erecta	7220	TR:B3NGC4_DROER
				Drosophila melanogaster	7227	TR:F0JAN7_DROME
						TR:Q26459_DROME
						TR:Q8IGD4_DROME
						TR:Q9VRM5_DROME
						TR:Q8IQ63_DROME
Peptidase C2, calpain family	IPR022684	2050	1	*Drosophila ananassae*	7217	TR:B3MXY5_DROAN
Cysteine peptidase, cysteine active site	IPR000169	7906	1	*Drosophila ananassae*	7217	TR:B3MXY5_DROAN
Peptidase C2, calpain, catalytic domain	IPR001300	2584	1	*Drosophila ananassae*	7217	TR:B3MXY5_DROAN
Zinc finger, RanBP2-type	IPR001876	5271	3	*Drosophila ananassae*	7217	TR:B3MXY5_DROAN
				Tetrahymena thermophila	312017	TR:I7MB64_TETTS
				Sulfolobus acidocaldarius	330779	TR:Q4J9H4_SULAC
COPII coat assembly protein, Sec16	IPR024880	475	2	*Drosophila ananassae*	7217	TR:B3MRZ5_DROAN
				Drosophila grimshawi	7222	TR:B4JNJ2_DROGR
Single-stranded nucleic acid binding R3H	IPR001374	4026	3	*Drosophila ananassae*	7217	TR:B3M803_DROAN
				Drosophila persimilis	7234	TR:B4GS08_DROPE
				Drosophila grimshawi	7222	TR:B4IZW4_DROGR
SUZ domain	IPR024771	586	3	*Drosophila ananassae*	7217	TR:B3M803_DROAN
				Drosophila persimilis	7234	TR:B4GS08_DROPE
				Drosophila grimshawi	7222	TR:B4IZW4_DROGR
SH2 domain	IPR000980	10236	6	*Drosophila sechellia*	7238	TR:B4ID22_DROSE
				Drosophila simulans	7240	TR:B4Q6K2_DROSI
				Drosophila erecta	7220	TR:B3N892_DROER
				Drosophila melanogaster	7227	TR:Q9VPU1_DROME
				Drosophila grimshawi	7222	TR:B4JNB2_DROGR
				Branchiostoma floridae	7739	TR:C3ZBA3_BRAFL

BTB/POZ-like	IPR000210	20275	4	*Drosophila sechellia*	7238	TR:B4IGL8_DROSE
				Drosophila simulans	7240	TR:B4R414_DROSI
				Drosophila yakuba	7245	TR:B4Q201_DROYA
				Drosophila melanogaster	7227	TR:Q9I7S4_DROME
BTB/POZ fold	IPR011333	24169	4	*Drosophila sechellia*	7238	TR:B4IGL8_DROSE
				Drosophila simulans	7240	TR:B4R414_DROSI
				Drosophila yakuba	7245	TR:B4Q201_DROYA
				Drosophila melanogaster	7227	TR:Q9I7S4_DROME
BTB/POZ	IPR013069	16378	4	*Drosophila sechellia*	7238	TR:B4IGL8_DROSE
				Drosophila simulans	7240	TR:B4R414_DROSI
				Drosophila yakuba	7245	TR:B4Q201_DROYA
				Drosophila melanogaster	7227	TR:Q9I7S4_DROME
Rap GTPase activating proteins domain	IPR000331	1449	4	*Drosophila sechellia*	7238	TR:B4IGL8_DROSE
				Drosophila simulans	7240	TR:B4R414_DROSI
				Drosophila yakuba	7245	TR:B4Q201_DROYA
				Drosophila melanogaster	7227	TR:Q9I7S4_DROME
Inositol polyphosphate kinase	IPR005522	1250	5	*Drosophila sechellia*	7238	TR:B4I7T1_DROSE
				Drosophila simulans	7240	TR:B4QGI0_DROSI
				Drosophila yakuba	7245	TR:B4P8W1_DROYA
				Drosophila erecta	7220	TR:B3NMY7_DROER
				Drosophila melanogaster	7227	TR:Q9W2E9_DROME
Seven cysteines	IPR013980	333	2	*Drosophila sechellia*	7238	TR:B4HWV4_DROSE
				Drosophila melanogaster	7227	TR:Q9Y110_DROME
Seven cysteines, N-terminal	IPR011106	292	2	*Drosophila sechellia*	7238	TR:B4HWV4_DROSE
				Drosophila melanogaster	7227	TR:Q9Y110_DROME
Wnt	IPR005817	11699	2	*Drosophila sechellia*	7238	TR:B4HY51_DROSE
				Drosophila simulans	7240	TR:B4Q5E7_DROSI
Wnt-10 protein	IPR013302	188	2	*Drosophila sechellia*	7238	TR:B4HY51_DROSE
				Drosophila simulans	7240	TR:B4Q5E7_DROSI
Wnt protein, conserved site	IPR018161	2610	2	*Drosophila sechellia*	7238	TR:B4HY51_DROSE
				Drosophila simulans	7240	TR:B4Q5E7_DROSI
Leucine rich repeat 4	IPR025875	15629	5	*Drosophila sechellia*	7238	TR:B4HTZ7_DROSE
				Drosophila yakuba	7245	TR:B4PHP3_DROYA
				Drosophila yakuba	7245	TR:B4PHP3_DROYA
				Drosophila erecta	7220	TR:B3NG33_DROER

				Yarrowia lipolytica	284591	TR:Q6C1M0_YARLI
U2A'/phosphoprotein 32 family A, C-terminal	IPR003603	1754	3	*Drosophila sechellia*	7238	TR:B4HTZ7_DROSE
				Drosophila simulans	7240	TR:B4QQ40_DROSI
				Drosophila erecta	7220	TR:B3NG33_DROER
WW domain	IPR001202	8029	14	*Drosophila simulans*	7240	TR:B4QFM5_DROSI
				Drosophila willistoni	7260	TR:B4MKD0_DROWI
				Arabidopsis thaliana	3702	SP:RH40_ARATH
				Arabidopsis lyrata subsp. lyrata	81972	TR:D7L5I4_ARALL
				Lodderomyces elongisporus	379508	TR:A5DRZ7_LODEL
				Cryptococcus neoformans var. neoformans	283643	TR:F5HHK8_CRYNB
						TR:Q5KNA4_CRYNJ
				Tetrapisispora phaffii	1071381	TR:G8BS80_TETPH
				Lachancea thermotolerans	559295	TR:C5DBI0_LACTC
				Mixia osmundae	764103	TR:G7EAV5_MIXOS
				Cryptococcus neoformans var. grubii	235443	TR:J9VX35_CRYNV
				Glarea lozoyensis	1104152	TR:H0EF05_GLAL7
				Polysphondylium pallidum	13642	TR:D3BU61_POLPA
				Dictyostelium fasciculatum	1054147	TR:F4PU20_DICFS
2OGFeDO domain, nucleic acid-modifying type	IPR024779	347	4	*Drosophila simulans*	7240	TR:B4QNM4_DROSI
				Drosophila melanogaster	7227	TR:Q9VZX4_DROME
						TR:Q8T0I1_DROME
						TR:Q8T9G4_DROME
Cleavage stimulation factor subunit 2, hinge domain	IPR025742	495	2	*Drosophila persimilis*	7234	TR:B4G5G9_DROPE
				Drosophila pseudoobscura pseudoobscura	46245	TR:Q299N5_DROPS
Transcription termination and cleavage factor C-terminal domain	IPR026896	381	2	*Drosophila persimilis*	7234	TR:B4G5G9_DROPE
				Drosophila pseudoobscura pseudoobscura	46245	TR:Q299N5_DROPS
E2F Family	IPR015633	962	2	*Drosophila persimilis*	7234	TR:B4GDR3_DROPE
				Drosophila pseudoobscura pseudoobscura	46245	TR:Q295K4_DROPS
Transcription factor E2F/ dimerisation partner	IPR003316	1350	2	*Drosophila persimilis*	7234	TR:B4GDR3_DROPE
				Drosophila pseudoobscura pseudoobscura	46245	TR:Q295K4_DROPS
Winged helix-turn-helix transcription repressor DNA-binding domain	IPR011991	772973	4	*Drosophila persimilis*	7234	TR:B4GDR3_DROPE
				Drosophila pseudoobscura pseudoobscura	46245	TR:Q295K4_DROPS
				Candida dubliniensis	573826	TR:B9WKI8_CANDC
				Thalassiosira pseudonana	35128	TR:B8LD72_THAPS
Protease-associated domain, PA	IPR003137	6308	2	*Drosophila persimilis*	7234	TR:B4GI37_DROPE
				Drosophila pseudoobscura pseudoobscura	46245	TR:Q28Z87_DROPS

Initiation factor eIF-4 gamma, MA3	IPR003891	1618	3	*Drosophila persimilis*	7234	TR:B4G502_DROPE
				Drosophila pseudoobscura pseudoobscura	46245	TR:B5DYF9_DROPS
				Rhizopus delemar	246409	TR:I1CEW0_RHIO9
MIF4-like, type 1/2/3	IPR016021	4469	3	*Drosophila persimilis*	7234	TR:B4G502_DROPE
				Drosophila pseudoobscura pseudoobscura	46245	TR:B5DYF9_DROPS
				Rhizopus delemar	246409	TR:I1CEW0_RHIO9
MIF4G-like, type 3	IPR003890	2636	3	*Drosophila persimilis*	7234	TR:B4G502_DROPE
				Drosophila pseudoobscura pseudoobscura	46245	TR:B5DYF9_DROPS
				Rhizopus delemar	246409	TR:I1CEW0_RHIO9
Protein kinase-like domain	IPR011009	205404	23	*Drosophila persimilis*	7234	TR:B4G6Q5_DROPE
				Drosophila pseudoobscura pseudoobscura	46245	TR:I5ANW1_DROPS
				Drosophila willistoni	7260	TR:B4NEN2_DROWI
				Volvox carteri	3067	TR:D8UGB6_VOLCA
				Clavispora lusitaniae	306902	TR:C4Y9B9_CLAL4
				Gaeumannomyces graminis var. tritici	644352	TR:J3P6H6_GAGT3
				Candida dubliniensis	573826	TR:B9WBA1_CANDC
				Candida parapsilosis	578454	TR:G8B5E8_CANPC
				Podospora anserina	515849	TR:B2AX04_PODAN
				Beauveria bassiana	655819	TR:J5JXA4_BEAB2
				Candida albicans	294748	TR:C4YSR4_CANAW
				Wuchereria bancrofti	6293	TR:J9EPD0_WUCBA
				Trichinella spiralis	6334	TR:E5S526_TRISP
				Polysphondylium pallidum	13642	TR:D3B3V6_POLPA
				Dictyostelium discoideum	44689	SP:FRAY2_DICDI
						SP:Y9844_DICDI
						SP:PKD1_DICDI
						SP:PLK_DICDI
				Paramecium tetraurelia	5888	TR:A0D439_PARTE
						TR:A0BWN9_PARTE
				Oxytricha trifallax	1172189	TR:J9I6Z1_9SPIT
						TR:J9FQG1_9SPIT
						TR:J9HUE1_9SPIT
Mad3/BUB1 homology region 1	IPR013212	511	2	*Drosophila persimilis*	7234	TR:B4G6Q5_DROPE
				Drosophila pseudoobscura pseudoobscura	46245	TR:B5DVC6_DROPS
Protein kinase, catalytic domain	IPR000719	155751	23	*Drosophila persimilis*	7234	TR:B4G6Q5_DROPE
				Drosophila pseudoobscura pseudoobscura	46245	TR:I5ANW1_DROPS
				Drosophila willistoni	7260	TR:B4NEN2_DROWI
				Volvox carteri	3067	TR:D8UGB6_VOLCA
				Candida parapsilosis	578454	TR:G8B5E8_CANPC

				Podospora anserina	515849	TR:B2AX04_PODAN
				Candida dubliniensis	573826	TR:B9WBA1_CANDC
				Gaeumannomyces graminis var. tritici	644352	TR:J3P6H6_GAGT3
				Clavispora lusitaniae	306902	TR:C4Y9B9_CLAL4
				Beauveria bassiana	655819	TR:J5JXA4_BEAB2
				Candida albicans	294748	TR:C4YSR4_CANAW
				Wuchereria bancrofti	6293	TR:J9EPD0_WUCBA
				Trichinella spiralis	6334	TR:E5S526_TRISP
				Polysphondylium pallidum	13642	TR:D3B3V6_POLPA
				Dictyostelium discoideum	44689	SP:FRAY2_DICDI
						SP:Y9844_DICDI
						SP:PKD1_DICDI
						SP:PLK_DICDI
				Paramecium tetraurelia	5888	TR:A0D439_PARTE
						TR:A0BWN9_PARTE
				Oxytricha trifallax	1172189	TR:J9I6Z1_9SPIT
						TR:J9FQG1_9SPIT
						TR:J9HUE1_9SPIT
Protein kinase, ATP binding site	IPR017441	88650	17	*Drosophila persimilis*	7234	TR:B4G6Q5_DROPE
				Drosophila pseudoobscura pseudoobscura	46245	TR:I5ANW1_DROPS
				Drosophila willistoni	7260	TR:B4NEN2_DROWI
				Gaeumannomyces graminis var. tritici	644352	TR:J3P6H6_GAGT3
				Clavispora lusitaniae	306902	TR:C4Y9B9_CLAL4
				Candida parapsilosis	578454	TR:G8B5E8_CANPC
				Podospora anserina	515849	TR:B2AX04_PODAN
				Candida albicans	294748	TR:C4YSR4_CANAW
				Trichinella spiralis	6334	TR:E5S526_TRISP
				Polysphondylium pallidum	13642	TR:D3B3V6_POLPA
				Dictyostelium discoideum	44689	SP:FRAY2_DICDI
						SP:Y9844_DICDI
						SP:PLK_DICDI
				Paramecium tetraurelia	5888	TR:A0D439_PARTE
						TR:A0BWN9_PARTE
				Oxytricha trifallax	1172189	TR:J9I6Z1_9SPIT
						TR:J9FQG1_9SPIT
Serine/threonine-protein kinase, active site	IPR008271	101014	19	*Drosophila persimilis*	7234	TR:B4G6Q5_DROPE
				Drosophila pseudoobscura pseudoobscura	46245	TR:I5ANW1_DROPS
				Drosophila willistoni	7260	TR:B4NEN2_DROWI
				Candida dubliniensis	573826	TR:B9WBA1_CANDC
				Gaeumannomyces graminis var. tritici	644352	TR:J3P6H6_GAGT3
				Clavispora lusitaniae	306902	TR:C4Y9B9_CLAL4
				Candida parapsilosis	578454	TR:G8B5E8_CANPC
				Candida albicans	294748	TR:C4YSR4_CANAW

				Wuchereria bancrofti	6293	TR:J9EPD0_WUCBA
				Trichinella spiralis	6334	TR:E5S526_TRISP
				Polysphondylium pallidum	13642	TR:D3B3V6_POLPA
				Dictyostelium discoideum	44689	SP:Y9844_DICDI
						SP:PKD1_DICDI
						SP:PLK_DICDI
				Paramecium tetraurelia	5888	TR:A0D439_PARTE
						TR:A0BWN9_PARTE
				Oxytricha trifallax	1172189	TR:J9I6Z1_9SPIT
						TR:J9FQG1_9SPIT
						TR:J9HUE1_9SPIT
Mitotic checkpoint serine/threonine protein kinase Bub1/Mitotic spindle checkpoint component Mad3	IPR015661	555	2	*Drosophila persimilis*	7234	TR:B4G6Q5_DROPE
				Drosophila pseudoobscura pseudoobscura	46245	TR:B5DVC6_DROPS
B30.2/SPRY domain	IPR001870	8684	1	*Drosophila pseudoobscura pseudoobscura*	46245	TR:Q28Z53_DROPS
Concanavalin A-like lectin/glucanases superfamily	IPR008985	59437	1	*Drosophila pseudoobscura pseudoobscura*	46245	TR:Q28Z53_DROPS
SAP domain	IPR003034	5302	2	*Drosophila pseudoobscura pseudoobscura*	46245	TR:Q28Z53_DROPS
				Candida albicans	237561	TR:Q5A8Y9_CANAL
SPla/RYanodine receptor SPRY	IPR003877	8592	1	*Drosophila pseudoobscura pseudoobscura*	46245	TR:Q28Z53_DROPS
Peptidase C19, ubiquitin carboxyl-terminal hydrolase 2, conserved site	IPR018200	8328	1	*Drosophila pseudoobscura pseudoobscura*	46245	TR:Q2LZV0_DROPS
Peptidase C19, ubiquitin carboxyl-terminal hydrolase 2	IPR001394	10281	1	*Drosophila pseudoobscura pseudoobscura*	46245	TR:Q2LZV0_DROPS
Serine/threonine- / dual specificity protein kinase, catalytic domain	IPR002290	46148	9	*Drosophila pseudoobscura pseudoobscura*	46245	TR:I5ANW1_DROPS
				Candida dubliniensis	573826	TR:B9WBA1_CANDC
				Gaeumannomyces graminis var. tritici	644352	TR:J3P6H6_GAGT3
				Clavispora lusitaniae	306902	TR:C4Y9B9_CLAL4
				Trichinella spiralis	6334	TR:E5S526_TRISP
				Dictyostelium discoideum	44689	SP:FRAY2_DICDI
						SP:PKD1_DICDI
						SP:PLK_DICDI
				Paramecium tetraurelia	5888	TR:A0D439_PARTE
Basic-leucine zipper domain	IPR004827	11371	4	*Drosophila willistoni*	7260	TR:B4MYE3_DROWI
				Oryza brachyantha	4533	TR:J3NC10_ORYBR
				Wickerhamomyces ciferrii	1206466	TR:K0KJU9_9ASCO

				Candida tropicalis	294747	TR:C5ME60_CANTT
Guanylate kinase	IPR008144	10579	1	*Drosophila willistoni*	7260	TR:B4NBV6_DROWI
Guanylate kinase/L-type calcium channel	IPR008145	10614	1	*Drosophila willistoni*	7260	TR:B4NBV6_DROWI
Guanylate kinase, conserved site	IPR020590	7776	1	*Drosophila willistoni*	7260	TR:B4NBV6_DROWI
L27	IPR004172	1263	1	*Drosophila willistoni*	7260	TR:B4NBV6_DROWI
L27, C-terminal	IPR014775	904	1	*Drosophila willistoni*	7260	TR:B4NBV6_DROWI
Variant SH3	IPR011511	6612	2	*Drosophila willistoni*	7260	TR:B4NBV6_DROWI
				Drosophila grimshawi	7222	TR:B4JVH5_DROGR
Coronin	IPR015505	1011	1	*Drosophila willistoni*	7260	TR:B4MJ97_DROWI
Domain of unknown function DUF1899	IPR015048	761	1	*Drosophila willistoni*	7260	TR:B4MJ97_DROWI
Domain of unknown function DUF1900	IPR015049	922	1	*Drosophila willistoni*	7260	TR:B4MJ97_DROWI
WD40/YVTN repeat-like-containing domain	IPR015943	109781	20	*Drosophila willistoni*	7260	TR:B4MJ97_DROWI
				Zea mays	4577	TR:B6SPQ9_MAIZE
						TR:C0P3D9_MAIZE
				Saccharomyces cerevisiae	764102	TR:E7Q1H5_YEASB
						SP:TUP1_YEAST
						TR:A6ZTQ6_YEAS7
						TR:B3LUD8_YEAS1
						TR:B5VF12_YEAS6
						TR:C7GUW4_YEAS2
						TR:C8Z4H1_YEAS8
						TR:E7NFE6_YEASO
						TR:E7QC78_YEASZ
						TR:H0GD95_9SACH
						TR:G2WA92_YEASK
						TR:E7KA75_YEASA
				Saccharomyces arboricola	1160507	TR:J8Q781_SACAR
				Kitasatospora setae	452652	TR:E4N1V7_KITSK
				Paramecium tetraurelia	5888	TR:A0CXK4_PARTE
						TR:A0CYP9_PARTE
						TR:A0E4C1_PARTE
WD40 repeat	IPR001680	70208	19	*Drosophila willistoni*	7260	TR:B4MJ97_DROWI
				Zea mays	4577	TR:B6SPQ9_MAIZE
						TR:C0P3D9_MAIZE

				Saccharomyces cerevisiae	764102	TR:E7Q1H5_YEASB
						SP:TUP1_YEAST
						TR:A6ZTQ6_YEAS7
						TR:B3LUD8_YEAS1
						TR:B5VF12_YEAS6
						TR:C7GUW4_YEAS2
						TR:C8Z4H1_YEAS8
						TR:E7NFE6_YEASO
						TR:E7QC78_YEASZ
						TR:H0GD95_9SACH
						TR:G2WA92_YEASK
						TR:E7KA75_YEASA
				Saccharomyces arboricola	1160507	TR:J8Q781_SACAR
				Paramecium tetraurelia	5888	TR:A0CXK4_PARTE
						TR:A0CYP9_PARTE
						TR:A0E4C1_PARTE
WD40 repeat, conserved site	IPR019775	39072	19	*Drosophila willistoni*	7260	TR:B4MJ97_DROWI
				Zea mays	4577	TR:B6SPQ9_MAIZE
						TR:C0P3D9_MAIZE
				Saccharomyces cerevisiae	764102	TR:E7Q1H5_YEASB
						SP:TUP1_YEAST
						TR:A6ZTQ6_YEAS7
						TR:B3LUD8_YEAS1
						TR:B5VF12_YEAS6
						TR:C7GUW4_YEAS2
						TR:C8Z4H1_YEAS8
						TR:E7NFE6_YEASO
						TR:E7QC78_YEASZ
						TR:H0GD95_9SACH
						TR:G2WA92_YEASK
						TR:E7KA75_YEASA
				Saccharomyces arboricola	1160507	TR:J8Q781_SACAR
				Paramecium tetraurelia	5888	TR:A0CXK4_PARTE
						TR:A0CYP9_PARTE
						TR:A0E4C1_PARTE
WD40-repeat-containing domain	IPR017986	74523	19	*Drosophila willistoni*	7260	TR:B4MJ97_DROWI
				Zea mays	4577	TR:B6SPQ9_MAIZE
						TR:C0P3D9_MAIZE
				Saccharomyces cerevisiae	764102	TR:E7Q1H5_YEASB
						SP:TUP1_YEAST
						TR:A6ZTQ6_YEAS7
						TR:B3LUD8_YEAS1
						TR:B5VF12_YEAS6

						TR:C7GUW4_YEAS2
						TR:C8Z4H1_YEAS8
						TR:E7NFE6_YEASO
						TR:E7QC78_YEASZ
						TR:H0GD95_9SACH
						TR:G2WA92_YEASK
						TR:E7KA75_YEASA
				Saccharomyces arboricola	1160507	TR:J8Q781_SACAR
				Paramecium tetraurelia	5888	TR:A0CXK4_PARTE
						TR:A0CYP9_PARTE
						TR:A0E4C1_PARTE
RNA helicase UPF1, UPF2-interacting domain	IPR018999	364	1	*Drosophila willistoni*	7260	TR:B4NDR4_DROWI
GRAM	IPR004182	2817	1	*Drosophila willistoni*	7260	TR:B4NLS4_DROWI
mRNA decapping protein 2, Box A	IPR007722	359	1	*Drosophila willistoni*	7260	TR:B4MXW4_DROWI
NUDIX hydrolase, conserved site	IPR020084	36499	1	*Drosophila willistoni*	7260	TR:B4MXW4_DROWI
NUDIX hydrolase domain	IPR000086	60977	2	*Drosophila willistoni*	7260	TR:B4MXW4_DROWI
				Dictyostelium fasciculatum	1054147	TR:F4Q5V6_DICFS
NUDIX hydrolase domain-like	IPR015797	67351	2	*Drosophila willistoni*	7260	TR:B4MXW4_DROWI
				Dictyostelium fasciculatum	1054147	TR:F4Q5V6_DICFS
Peptidase A22, presenilin signal peptide	IPR006639	1285	1	*Drosophila willistoni*	7260	TR:B4N6M7_DROWI
Peptidase A22A, presenilin	IPR001108	404	1	*Drosophila willistoni*	7260	TR:B4N6M7_DROWI
Paramecium bursaria Chlorella virus 1, A79R	IPR009820	55	1	*Drosophila willistoni*	7260	TR:B4MIR0_DROWI
C2 membrane targeting protein	IPR018029	13871	6	*Drosophila erecta*	7220	TR:B3P3P0_DROER
				Polysphondylium pallidum	13642	TR:D3BU61_POLPA
				Dictyostelium fasciculatum	1054147	TR:F4PU20_DICFS
				Oxytricha trifallax	1172189	TR:J9EJ94_9SPIT
				Naegleria gruberi	5762	TR:D2VCR4_NAEGR
				Trichomonas vaginalis	5722	TR:A2EUT8_TRIVA
Synaptotagmin	IPR001565	1408	1	*Drosophila erecta*	7220	TR:B3P3P0_DROER
Transcription factor CBF/NF-Y/archaeal histone	IPR003958	3170	1	*Drosophila affinis*	7246	TR:D9MUS3_DROAI
Histone-fold	IPR009072	27659	4	*Drosophila affinis*	7246	TR:D9MUS3_DROAI
				Chaetomium thermophilum	759272	TR:G0S2A1_CHATD

				Grosmannia clavigera	655863	TR:F0XN84_GROCL
				Dictyostelium discoideum	44689	SP:TAF12_DICDI
Histone acetyltransferases subunit 3	IPR019340	280	1	*Drosophila grimshawi*	7222	TR:B4JJ95_DROGR
Thyroid hormone receptor	IPR001728	734	1	*Drosophila grimshawi*	7222	TR:B4J0N8_DROGR
SOCS protein, C-terminal	IPR001496	2535	1	*Drosophila grimshawi*	7222	TR:B4JNB2_DROGR
Dorsal protein	IPR011363	39	1	*Drosophila grimshawi*	7222	TR:B4JD22_DROGR
Immunoglobulin E-set	IPR014756	54860	2	*Drosophila grimshawi*	7222	TR:B4JD22_DROGR
				Dictyostelium purpureum	5786	TR:F0ZYQ8_DICPU
Cell surface receptor IPT/TIG	IPR002909	4608	2	*Drosophila grimshawi*	7222	TR:B4JD22_DROGR
				Dictyostelium purpureum	5786	TR:F0ZYQ8_DICPU
NF-kappa-B/Rel/Dorsal	IPR000451	622	1	*Drosophila grimshawi*	7222	TR:B4JD22_DROGR
p53-like transcription factor, DNA- binding	IPR008967	5876	4	*Drosophila grimshawi*	7222	TR:B4JD22_DROGR
				Branchiostoma floridae	7739	TR:C3ZBA3_BRAFL
				Lodderomyces elongisporus	379508	TR:A5H2N9_LODEL
				Spathaspora passalidarum	619300	TR:G3AKA5_SPAPN
Rel homology domain	IPR011539	1184	1	*Drosophila grimshawi*	7222	TR:B4JD22_DROGR
Sec16, central conserved domain	IPR024340	391	3	*Drosophila grimshawi*	7222	TR:B4JNJ2_DROGR
				Komagataella pastoris	981350	TR:F2R092_PICP7
				Komagataella pastoris	4922	TR:Q45TY0_PICPA
Bifunctional inhibitor/plant lipid transfer protein/seed storage helical domain	IPR016140	7058	197	*Triticum aestivum*	4565	TR:Q0ZCA8_WHEAT
						TR:Q0ZCB0_WHEAT
						TR:Q5TLY8_WHEAT
						TR:B3EY90_WHEAT
						TR:Q5MFP7_WHEAT
						TR:Q41551_WHEAT
						TR:Q5MFP8_WHEAT
						TR:D0EVP6_WHEAT
						TR:G3F3Z1_WHEAT
						TR:G3F3Z3_WHEAT
						TR:I3RTU0_WHEAT
						TR:Q5MFQ5_WHEAT
						TR:I1XB56_WHEAT
						TR:D3UAL6_WHEAT
						TR:F8SGN9_WHEAT

						TR:Q8W3W0_WHEAT
						TR:D0EVP1_WHEAT
						TR:C5IXL3_WHEAT
						TR:F8SGL6_WHEAT
						TR:F8SGL8_WHEAT
						TR:F8SGL9_WHEAT
						TR:Q571Q5_WHEAT
						TR:F8SGL7_WHEAT
						TR:Q18NR2_WHEAT
						TR:Q68AN2_WHEAT
						TR:Q75ZV8_WHEAT
						TR:D3UAL8_WHEAT
						TR:Q8W3W1_WHEAT
						TR:B3EY89_WHEAT
						TR:B5TWK5_WHEAT
						TR:Q5MFQ4_WHEAT
						TR:Q5MFQ6_WHEAT
						TR:B2BZC4_WHEAT
						TR:B2BZD2_WHEAT
						TR:B2Y2S1_WHEAT
						TR:B2Y2S6_WHEAT
						TR:B3EY88_WHEAT
						TR:B5TWK4_WHEAT
						TR:C3VN79_WHEAT
						TR:C3VN80_WHEAT
						TR:D3U318_WHEAT
						TR:F8SGL2_WHEAT
						TR:F8SGL3_WHEAT
						TR:F8SGL4_WHEAT
						TR:F8SGM0_WHEAT
						TR:F8SGM5_WHEAT
						TR:F8SGM7_WHEAT
						TR:F8SGM8_WHEAT
						TR:F8SGM9_WHEAT
						TR:F8SGN0_WHEAT
						TR:F8SGP6_WHEAT
						TR:F8SGP8_WHEAT
						TR:F8SGQ1_WHEAT
						TR:F8SGQ2_WHEAT
						TR:I3RTU2_WHEAT
						TR:Q00M61_WHEAT
						TR:Q84U20_WHEAT
						TR:Q8W3W2_WHEAT
						TR:D0EVP0_WHEAT
						TR:D0EVP2_WHEAT

						TR:D0EVP4_WHEAT
						TR:Q5MFQ1_WHEAT
						TR:Q5MFQ2_WHEAT
						TR:B2Y2Q1_WHEAT
						TR:B2Y2Q4_WHEAT
						TR:B2Y2Q3_WHEAT
						TR:Q8W3V6_WHEAT
						TR:B2Y2R5_WHEAT
						TR:B2Y2Q2_WHEAT
						TR:Q9ZNY0_WHEAT
						TR:Q8H0J4_WHEAT
						TR:O22108_WHEAT
						TR:F6M7E1_WHEAT
						TR:H9XH02_WHEAT
						TR:D2DII8_WHEAT
						TR:A4KZ73_WHEAT
						TR:H9XGZ8_WHEAT
						TR:Q52NZ4_WHEAT
						TR:Q8W3V8_WHEAT
						TR:Q8W3V9_WHEAT
						TR:D2DII5_WHEAT
						TR:D3U315_WHEAT
						TR:D2DII6_WHEAT
						TR:Q8GU18_WHEAT
						TR:Q41550_WHEAT
						TR:B5ANT3_WHEAT
				Triticum aestivum subsp. yunnanense	425487	TR:A8CA05_WHEAT
						TR:A9YSH4_WHEAT
				Triticum aestivum subsp. tibeticum	231718	TR:Q7Y075_WHEAT
				Triticum turgidum subsp. dicoccon	49225	TR:D5FPE5_TRITU
				Triticum turgidum	4571	TR:Q0Q2J0_TRITU
						TR:Q0Q2J1_TRITU
						TR:A7XDG0_TRITU
						TR:A7X9X7_TRITU
				Triticum turgidum subsp. durum	4567	TR:D5FPE4_TRIDB
						TR:O49958_TRIDB
						TR:Q9XGE9_TRIDB
						TR:Q9FEQ1_TRIDB
						TR:Q41603_TRIDB
				Triticum monococcum	4568	TR:B8XU58_TRIMO
						TR:B8XU59_TRIMO
						TR:I7G262_TRIMO
						TR:B8XU60_TRIMO
				Triticum dicoccoides	85692	TR:B8Y0L5_TRIDC
						TR:D5FPE0_TRIDC

						TR:B6UKK3_TRIDC
						TR:B6UKJ8_TRIDC
						TR:B6UKK0_TRIDC
						TR:B6UKK1_TRIDC
						TR:B6UKK4_TRIDC
						TR:B6UKK6_TRIDC
						TR:B6UKK9_TRIDC
						TR:B6UKL0_TRIDC
						TR:A2IBJ9_TRIDC
						TR:D5FPE7_TRIDC
				Triticum zhukovskyi	77602	TR:A2IBK1_9POAL
						TR:A2IBK1_9POAL
						TR:Q84U13_9POAL
				Leymus mollis	183794	TR:C6K8R5_9POAL
						TR:F5A7G7_9POAL
						TR:F5A7G8_9POAL
						TR:F5A7G6_9POAL
				Secale sylvestre	4552	TR:Q5MGR3_9POAL
						TR:Q5MGR4_9POAL
				Triticum polonicum	77606	TR:A2TN60_9POAL
				Thinopyrum intermedium	85679	TR:E5FEZ7_9POAL
						TR:Q84U17_9POAL
				Dasypyrum villosum	40247	TR:A9Z0K7_9POAL
						TR:F6KHQ1_9POAL
						TR:J9QGN7_9POAL
						TR:F8S8T4_9POAL
						TR:F8S8T7_9POAL
						TR:F8S8U3_9POAL
						TR:F8S8U4_9POAL
						TR:F8S8U6_9POAL
						TR:J9QFU9_9POAL
						TR:J9QGY5_9POAL
						TR:F6KHQ0_9POAL
						TR:F8S8T5_9POAL
						TR:F8S8T6_9POAL
						TR:F8S8T8_9POAL
						TR:F8S8T9_9POAL
						TR:F8S8U0_9POAL
						TR:F8S8U1_9POAL
						TR:F8S8U2_9POAL
						TR:F8S8U5_9POAL
						TR:F8S8U7_9POAL
						TR:J9QFV1_9POAL
						TR:J9QEK9_9POAL
						TR:J9QFG0_9POAL

						TR:A9Z0J9_9POAL
						TR:A9Z0K1_9POAL
						TR:A9Z0K4_9POAL
						TR:A9Z0K2_9POAL
						TR:A9Z0K5_9POAL
						TR:A9Z0K6_9POAL
						TR:A9Z0K8_9POAL
						TR:A9Z0K3_9POAL
				Dasypyrum hordeaceum	49449	TR:A8VZH1_9POAL
						TR:A8VZI4_9POAL
						TR:A9Z0L4_9POAL
						TR:A8VZF6_9POAL
						TR:D1MI66_9POAL
				Thinopyrum elongatum	4588	TR:A7LHA8_LOPEL
						TR:Q5XY00_LOPEL
				Hordeum vulgare	4513	SP:HOG3_HORVU
						TR:I6TEV2_HORVU
				Aegilops longissima	4486	TR:A9YWM3_AEGLO
						TR:D5FPF2_AEGLO
						TR:D5FPF0_AEGLO
						TR:C8CCM4_AEGLO
						TR:A2IBJ8_AEGLO
						TR:A9YWM2_AEGLO
						TR:A9YWM7_AEGLO
				Aegilops tauschii	37682	TR:Q5MB98_AEGTA
						TR:D3U335_AEGTA
						TR:Q6J6U9_AEGTA
						TR:B6UKJ3_AEGTA
						TR:B6UKJ4_AEGTA
						TR:B6UKI7_AEGTA
						TR:B6UKI9_AEGTA
						TR:B6UKJ1_AEGTA
						TR:B6UKJ5_AEGTA
						TR:D3U330_AEGTA
						TR:Q5MB99_AEGTA
				Aegilops tauschii x Triticum turgidum	285950	TR:Q6B780_9POAL
				Thinopyrum ponticum x Triticum aestivum	222994	TR:A8IEA6_9POAL
				Aegilops markgrafii	4494	TR:C8CCM6_9POAL
				Psathyrostachys huashanica	37730	TR:B8Y444_9POAL
						TR:E3UMZ0_9POAL
						TR:E3UMZ1_9POAL
				Psathyrostachys juncea	4586	TR:G0YLZ5_PSAJU
						TR:G0YLZ4_PSAJU
						TR:G0YLZ0_PSAJU
						TR:G0YLZ1_PSAJU

				Aegilops speltoides	4573	TR:C8CCM8_AEGSP
				Aegilops triuncialis	39391	TR:Q5MB97_AEGTR
Gliadin /Low molecular weight glutenin	IPR001954	2080	199	*Triticum aestivum*	4565	TR:Q0ZCA8_WHEAT
						TR:Q0ZCB0_WHEAT
						TR:Q5TLY8_WHEAT
						TR:B3EY90_WHEAT
						TR:Q5MFP7_WHEAT
						TR:Q41551_WHEAT
						TR:Q5MFP8_WHEAT
						TR:D0EVP6_WHEAT
						TR:G3F3Z1_WHEAT
						TR:G3F3Z3_WHEAT
						TR:I3RTU0_WHEAT
						TR:Q5MFQ5_WHEAT
						TR:I1XB56_WHEAT
						TR:D3UAL6_WHEAT
						TR:F8SGN9_WHEAT
						TR:Q8W3W0_WHEAT
						TR:D0EVP1_WHEAT
						TR:C5IXL3_WHEAT
						TR:F8SGL6_WHEAT
						TR:F8SGL8_WHEAT
						TR:F8SGL9_WHEAT
						TR:Q571Q5_WHEAT
						TR:F8SGL7_WHEAT
						TR:Q18NR2_WHEAT
						TR:Q68AN2_WHEAT
						TR:Q75ZV8_WHEAT
						TR:D3UAL8_WHEAT
						TR:Q8W3W1_WHEAT
						TR:B3EY89_WHEAT
						TR:B5TWK5_WHEAT
						TR:Q5MFQ4_WHEAT
						TR:Q5MFQ6_WHEAT
						TR:B2BZC4_WHEAT
						TR:B2BZD2_WHEAT
						TR:B2Y2S1_WHEAT
						TR:B2Y2S6_WHEAT
						TR:B3EY88_WHEAT
						TR:B5TWK4_WHEAT
						TR:C3VN79_WHEAT
						TR:C3VN80_WHEAT
						TR:D3U318_WHEAT
						TR:F8SGL2_WHEAT

						TR:F8SGL3_WHEAT
						TR:F8SGL4_WHEAT
						TR:F8SGM0_WHEAT
						TR:F8SGM5_WHEAT
						TR:F8SGM7_WHEAT
						TR:F8SGM8_WHEAT
						TR:F8SGM9_WHEAT
						TR:F8SGN0_WHEAT
						TR:F8SGP6_WHEAT
						TR:F8SGP8_WHEAT
						TR:F8SGQ1_WHEAT
						TR:F8SGQ2_WHEAT
						TR:I3RTU2_WHEAT
						TR:Q00M61_WHEAT
						TR:Q84U20_WHEAT
						TR:Q8W3W2_WHEAT
						TR:D0EVP0_WHEAT
						TR:D0EVP2_WHEAT
						TR:D0EVP4_WHEAT
						TR:Q5MFQ1_WHEAT
						TR:Q5MFQ2_WHEAT
						TR:B2Y2Q1_WHEAT
						TR:B2Y2Q4_WHEAT
						TR:B2Y2Q3_WHEAT
						TR:Q8W3V6_WHEAT
						TR:B2Y2R5_WHEAT
						TR:B2Y2Q2_WHEAT
						TR:Q9ZNY0_WHEAT
						TR:Q8H0J4_WHEAT
						TR:O22108_WHEAT
						TR:F6M7E1_WHEAT
						TR:H9XH02_WHEAT
						TR:D2DII8_WHEAT
						TR:A4KZ73_WHEAT
						TR:H9XGZ8_WHEAT
						TR:Q52NZ4_WHEAT
						TR:Q8W3V8_WHEAT
						TR:Q8W3V9_WHEAT
						TR:D2DII5_WHEAT
						TR:D3U315_WHEAT
						TR:D2DII6_WHEAT
						TR:Q8GU18_WHEAT
						TR:Q41550_WHEAT
						TR:B5ANT3_WHEAT
				Triticum aestivum subsp. yunnanense	425487	TR:A8CA05_WHEAT

						TR:A9YSH4_WHEAT
				Triticum aestivum subsp. tibeticum	231718	TR:Q7Y075_WHEAT
				Triticum turgidum subsp. dicoccon	49225	TR:D5FPE5_TRITU
				Triticum turgidum	4571	TR:Q0Q2J0_TRITU
						TR:Q0Q2J1_TRITU
						TR:A7XDG0_TRITU
						TR:A7X9X7_TRITU
				Triticum turgidum subsp. durum	4567	TR:D5FPE4_TRIDB
						TR:O49958_TRIDB
						TR:Q9XGE9_TRIDB
						TR:Q9FEQ1_TRIDB
						TR:Q41603_TRIDB
				Triticum monococcum	4568	TR:B8XU58_TRIMO
						TR:B8XU59_TRIMO
						TR:I7G262_TRIMO
						TR:B8XU60_TRIMO
				Triticum dicoccoides	85692	TR:B8Y0L5_TRIDC
						TR:D5FPE0_TRIDC
						TR:B6UKK3_TRIDC
						TR:B6UKJ8_TRIDC
						TR:B6UKK0_TRIDC
						TR:B6UKK1_TRIDC
						TR:B6UKK4_TRIDC
						TR:B6UKK6_TRIDC
						TR:B6UKK9_TRIDC
						TR:B6UKL0_TRIDC
						TR:A2IBJ9_TRIDC
						TR:D5FPE7_TRIDC
				Triticum zhukovskyi	77602	TR:A2IBK1_9POAL
						TR:A2IBK1_9POAL
				Triticum aestivum/Thinopyrum intermedium	218488	TR:Q84U13_9POAL
				Secale sylvestre	4552	TR:C6K8R5_9POAL
						TR:F5A7G7_9POAL
						TR:F5A7G8_9POAL
						TR:F5A7G6_9POAL
						TR:Q5MGR3_9POAL
						TR:Q5MGR4_9POAL
				Triticum polonicum	77606	TR:A2TN60_9POAL
				Thinopyrum intermedium	85679	TR:E5FEZ7_9POAL
						TR:Q84U17_9POAL
				Dasypyrum villosum	40247	TR:A9Z0K7_9POAL
						TR:F6KHQ1_9POAL
						TR:J9QGN7_9POAL
						TR:F8S8T4_9POAL
						TR:F8S8T7_9POAL

						TR:F8S8U3_9POAL
						TR:F8S8U4_9POAL
						TR:F8S8U6_9POAL
						TR:J9QFU9_9POAL
						TR:J9QGY5_9POAL
						TR:F6KHQ0_9POAL
						TR:F8S8T5_9POAL
						TR:F8S8T6_9POAL
						TR:F8S8T8_9POAL
						TR:F8S8T9_9POAL
						TR:F8S8U0_9POAL
						TR:F8S8U1_9POAL
						TR:F8S8U2_9POAL
						TR:F8S8U5_9POAL
						TR:F8S8U7_9POAL
						TR:J9QFV1_9POAL
						TR:J9QEK9_9POAL
						TR:J9QFG0_9POAL
						TR:A9Z0J9_9POAL
						TR:A9Z0K1_9POAL
						TR:A9Z0K4_9POAL
						TR:A9Z0K2_9POAL
						TR:A9Z0K5_9POAL
						TR:A9Z0K6_9POAL
						TR:A9Z0K8_9POAL
						TR:A9Z0K3_9POAL
				Dasypyrum hordeaceum	49449	TR:A8VZH1_9POAL
						TR:A8VZI4_9POAL
						TR:A9Z0L4_9POAL
						TR:A8VZF6_9POAL
						TR:D1MI66_9POAL
				Thinopyrum elongatum	4588	TR:A7LHA8_LOPEL
						TR:Q5XY00_LOPEL
				Hordeum vulgare	4513	SP:HOG3_HORVU
						TR:I6TEV2_HORVU
				Aegilops longissima	4486	TR:A9YWM3_AEGLO
						TR:D5FPF2_AEGLO
						TR:D5FPF0_AEGLO
						TR:C8CCM4_AEGLO
						TR:A2IBJ8_AEGLO
						TR:A9YWM2_AEGLO
						TR:A9YWM7_AEGLO
				Aegilops tauschii	37682	TR:Q5MB98_AEGTA
						TR:D3U335_AEGTA
						TR:Q6J6U9_AEGTA

						TR:B6UKJ3_AEGTA
						TR:B6UKJ4_AEGTA
						TR:B6UKI7_AEGTA
						TR:B6UKI9_AEGTA
						TR:B6UKJ1_AEGTA
						TR:B6UKJ5_AEGTA
						TR:D3U330_AEGTA
						TR:Q5MB99_AEGTA
				Aegilops tauschii x Triticum turgidum	285950	TR:Q6B780_9POAL
				Thinopyrum ponticum x Triticum aestivum	222994	TR:A8IEA6_9POAL
				Aegilops markgrafii	4494	TR:C8CCM6_9POAL
				Psathyrostachys huashanica	37730	TR:B8Y444_9POAL
						TR:E3UMZ0_9POAL
						TR:E3UMZ1_9POAL
				Psathyrostachys juncea	4586	TR:G0YLZ5_PSAJU
						TR:G0YLZ4_PSAJU
						TR:G0YLZ0_PSAJU
						TR:G0YLZ1_PSAJU
				Aegilops speltoides	4573	TR:C8CCM8_AEGSP
				Aegilops triuncialis	39391	TR:Q5MB97_AEGTR
Gliadin, alpha/beta	IPR001376	1965	208	*Triticum aestivum*	4565	TR:Q0ZCA8_WHEAT
						TR:Q0ZCB0_WHEAT
						TR:Q5TLY8_WHEAT
						TR:B3EY90_WHEAT
						TR:Q5MFP7_WHEAT
						TR:Q41551_WHEAT
						TR:Q5MFP8_WHEAT
						TR:B6ETS0_WHEAT
						TR:D0EVP6_WHEAT
						TR:G3F3Z1_WHEAT
						TR:G3F3Z3_WHEAT
						TR:I3RTU0_WHEAT
						TR:Q5MFQ5_WHEAT
						TR:I1XB56_WHEAT
						TR:D3UAL6_WHEAT
						TR:F8SGN9_WHEAT
						TR:Q8W3W0_WHEAT
						TR:D0EVP1_WHEAT
						TR:C5IXL3_WHEAT
						TR:F8SGL6_WHEAT
						TR:F8SGL8_WHEAT
						TR:F8SGL9_WHEAT
						TR:Q571Q5_WHEAT
						TR:F8SGL7_WHEAT

						TR:Q18NR2_WHEAT
						TR:Q68AN2_WHEAT
						TR:Q75ZV8_WHEAT
						TR:D3UAL8_WHEAT
						TR:B6ETS1_WHEAT
						TR:Q8W3W1_WHEAT
						TR:B3EY89_WHEAT
						TR:B5TWK5_WHEAT
						TR:Q5MFQ4_WHEAT
						TR:Q5MFQ6_WHEAT
						TR:B2BZC4_WHEAT
						TR:B2BZD2_WHEAT
						TR:B2Y2S1_WHEAT
						TR:B2Y2S6_WHEAT
						TR:B3EY88_WHEAT
						TR:B5TWK4_WHEAT
						TR:C3VN79_WHEAT
						TR:C3VN80_WHEAT
						TR:D3U318_WHEAT
						TR:F8SGL2_WHEAT
						TR:F8SGL3_WHEAT
						TR:F8SGL4_WHEAT
						TR:F8SGM0_WHEAT
						TR:F8SGM5_WHEAT
						TR:F8SGM7_WHEAT
						TR:F8SGM8_WHEAT
						TR:F8SGM9_WHEAT
						TR:F8SGN0_WHEAT
						TR:F8SGP6_WHEAT
						TR:F8SGP8_WHEAT
						TR:F8SGQ1_WHEAT
						TR:F8SGQ2_WHEAT
						TR:I3RTU2_WHEAT
						TR:Q00M61_WHEAT
						TR:Q84U20_WHEAT
						TR:Q8W3W2_WHEAT
						TR:D0EVP0_WHEAT
						TR:D0EVP2_WHEAT
						TR:D0EVP4_WHEAT
						TR:Q5MFQ1_WHEAT
						TR:Q5MFQ2_WHEAT
						TR:B2Y2Q1_WHEAT
						TR:B2Y2Q4_WHEAT
						TR:B2Y2Q3_WHEAT
						TR:Q402I5_WHEAT

						TR:Q8W3V6_WHEAT
						TR:B2Y2R5_WHEAT
						TR:B2Y2Q2_WHEAT
						TR:Q9ZNY0_WHEAT
						TR:Q8H0J4_WHEAT
						TR:O22108_WHEAT
						TR:F6M7E1_WHEAT
						TR:H9XH02_WHEAT
						TR:D2DII8_WHEAT
						TR:A4KZ73_WHEAT
						TR:H9XGZ8_WHEAT
						TR:Q52NZ4_WHEAT
						TR:Q8W3V8_WHEAT
						TR:Q8W3V9_WHEAT
						TR:D2DII5_WHEAT
						TR:D3U315_WHEAT
						TR:D2DII6_WHEAT
						TR:Q8GU18_WHEAT
						TR:Q41550_WHEAT
						TR:B5ANT3_WHEAT
				Triticum aestivum subsp. yunnanense	425487	TR:A8CA05_WHEAT
						TR:A9YSH4_WHEAT
				Triticum aestivum subsp. tibeticum	231718	TR:Q7Y075_WHEAT
				Triticum turgidum subsp. dicoccon	49225	TR:D5FPE5_TRITU
				Triticum turgidum	4571	TR:Q0Q2J0_TRITU
						TR:Q0Q2J1_TRITU
						TR:A7XDG0_TRITU
						TR:A7X9X7_TRITU
				Triticum turgidum subsp. durum	4567	TR:D5FPE4_TRIDB
						TR:O49958_TRIDB
						TR:Q9XGE9_TRIDB
						TR:Q9FEQ1_TRIDB
						TR:Q41603_TRIDB
				Triticum monococcum	4568	TR:B8XU58_TRIMO
						TR:B8XU59_TRIMO
						TR:I7G262_TRIMO
						TR:B8XU60_TRIMO
				Triticum dicoccoides	85692	TR:B8Y0L5_TRIDC
						TR:D5FPE0_TRIDC
						TR:B6UKK3_TRIDC
						TR:B6UKJ8_TRIDC
						TR:B6UKK0_TRIDC
						TR:B6UKK1_TRIDC
						TR:B6UKK4_TRIDC
						TR:B6UKK6_TRIDC

						TR:B6UKK9_TRIDC
						TR:B6UKL0_TRIDC
						TR:A2IBJ9_TRIDC
						TR:D5FPE7_TRIDC
				Triticum zhukovskyi	77602	TR:A2IBK1_9POAL
						TR:A2IBK1_9POAL
						TR:Q84U13_9POAL
				Leymus mollis	183794	TR:C6K8R5_9POAL
						TR:F5A7G7_9POAL
						TR:F5A7G8_9POAL
						TR:F5A7G6_9POAL
				Secale sylvestre	4552	TR:Q5MGR3_9POAL
						TR:Q5MGR4_9POAL
				Triticum polonicum	77606	TR:A2TN60_9POAL
				Thinopyrum intermedium	85679	TR:E5FEZ7_9POAL
						TR:Q84U17_9POAL
				Dasypyrum villosum	40247	TR:A9Z0K7_9POAL
						TR:F6KHQ1_9POAL
						TR:J9QGN7_9POAL
						TR:F8S8T4_9POAL
						TR:F8S8T7_9POAL
						TR:F8S8U3_9POAL
						TR:F8S8U4_9POAL
						TR:F8S8U6_9POAL
						TR:J9QFU9_9POAL
						TR:J9QGY5_9POAL
						TR:F6KHQ0_9POAL
						TR:F8S8T5_9POAL
						TR:F8S8T6_9POAL
						TR:F8S8T8_9POAL
						TR:F8S8T9_9POAL
						TR:F8S8U0_9POAL
						TR:F8S8U1_9POAL
						TR:F8S8U2_9POAL
						TR:F8S8U5_9POAL
						TR:F8S8U7_9POAL
						TR:J9QFV1_9POAL
						TR:J9QEK9_9POAL
						TR:J9QFG0_9POAL
						TR:A9Z0J9_9POAL
						TR:A9Z0K1_9POAL
						TR:A9Z0K4_9POAL
						TR:A9Z0K2_9POAL
						TR:A9Z0K5_9POAL
						TR:A9Z0K6_9POAL

						TR:A9Z0K8_9POAL
						TR:A9Z0K3_9POAL
				Dasypyrum hordeaceum	49449	TR:A8VZH1_9POAL
						TR:A8VZI4_9POAL
						TR:A9Z0L4_9POAL
						TR:A8VZF6_9POAL
						TR:D1MI66_9POAL
				Thinopyrum elongatum	4588	TR:G8G230_LOPEL
						TR:G8G227_LOPEL
						TR:G8G225_LOPEL
						TR:G8G229_LOPEL
						TR:G8G226_LOPEL
						TR:G8G231_LOPEL
						TR:G8G228_LOPEL
						TR:A7LHA8_LOPEL
						TR:Q5XY00_LOPEL
				Hordeum vulgare	4513	SP:HOG3_HORVU
						TR:I6TEV2_HORVU
				Aegilops longissima	4486	TR:A9YWM3_AEGLO
						TR:D5FPF2_AEGLO
						TR:D5FPF0_AEGLO
						TR:C8CCM4_AEGLO
						TR:A2IBJ8_AEGLO
						TR:A9YWM2_AEGLO
						TR:A9YWM7_AEGLO
				Aegilops tauschii	37682	TR:Q5MB98_AEGTA
						TR:D3U335_AEGTA
						TR:Q6J6U9_AEGTA
						TR:B6UKJ3_AEGTA
						TR:B6UKJ4_AEGTA
						TR:B6UKI7_AEGTA
						TR:B6UKI9_AEGTA
						TR:B6UKJ1_AEGTA
						TR:B6UKJ5_AEGTA
						TR:D3U330_AEGTA
						TR:Q5MB99_AEGTA
				Aegilops tauschii x Triticum turgidum	285950	TR:Q6B780_9POAL
				Thinopyrum ponticum x Triticum aestivum	222994	TR:A8IEA6_9POAL
				Aegilops markgrafii	4494	TR:C8CCM6_9POAL
				Psathyrostachys huashanica	37730	TR:B9VSH9_9POAL
						TR:B8Y444_9POAL
						TR:E3UMZ0_9POAL
						TR:E3UMZ1_9POAL
				Psathyrostachys juncea	4586	TR:G0YLZ5_PSAJU
						TR:G0YLZ4_PSAJU

						TR:G0YLZ0_PSAJU
						TR:G0YLZ1_PSAJU
				Aegilops speltoides	4573	TR:C8CCM8_AEGSP
				Aegilops triuncialis	39391	TR:Q5MB97_AEGTR
Bifunctional trypsin/alpha-amylase inhibitor helical domain	IPR013771	3533	199	*Triticum aestivum*	4565	TR:Q0ZCA8_WHEAT
						TR:Q0ZCB0_WHEAT
						TR:Q5TLY8_WHEAT
						TR:B3EY90_WHEAT
						TR:Q5MFP7_WHEAT
						TR:Q41551_WHEAT
						TR:Q5MFP8_WHEAT
						TR:D0EVP6_WHEAT
						TR:G3F3Z1_WHEAT
						TR:G3F3Z3_WHEAT
						TR:I3RTU0_WHEAT
						TR:Q5MFQ5_WHEAT
						TR:I1XB56_WHEAT
						TR:D3UAL6_WHEAT
						TR:F8SGN9_WHEAT
						TR:Q8W3W0_WHEAT
						TR:D0EVP1_WHEAT
						TR:C5IXL3_WHEAT
						TR:F8SGL6_WHEAT
						TR:F8SGL8_WHEAT
						TR:F8SGL9_WHEAT
						TR:Q571Q5_WHEAT
						TR:F8SGL7_WHEAT
						TR:Q18NR2_WHEAT
						TR:Q68AN2_WHEAT
						TR:Q75ZV8_WHEAT
						TR:D3UAL8_WHEAT
						TR:Q8W3W1_WHEAT
						TR:B3EY89_WHEAT
						TR:B5TWK5_WHEAT
						TR:Q5MFQ4_WHEAT
						TR:Q5MFQ6_WHEAT
						TR:B2BZC4_WHEAT
						TR:B2BZD2_WHEAT
						TR:B2Y2S1_WHEAT
						TR:B2Y2S6_WHEAT
						TR:B3EY88_WHEAT
						TR:B5TWK4_WHEAT
						TR:C3VN79_WHEAT
						TR:C3VN80_WHEAT

						TR:D3U318_WHEAT
						TR:F8SGL2_WHEAT
						TR:F8SGL3_WHEAT
						TR:F8SGL4_WHEAT
						TR:F8SGM0_WHEAT
						TR:F8SGM5_WHEAT
						TR:F8SGM7_WHEAT
						TR:F8SGM8_WHEAT
						TR:F8SGM9_WHEAT
						TR:F8SGN0_WHEAT
						TR:F8SGP6_WHEAT
						TR:F8SGP8_WHEAT
						TR:F8SGQ1_WHEAT
						TR:F8SGQ2_WHEAT
						TR:I3RTU2_WHEAT
						TR:Q00M61_WHEAT
						TR:Q84U20_WHEAT
						TR:Q8W3W2_WHEAT
						TR:D0EVP0_WHEAT
						TR:D0EVP2_WHEAT
						TR:D0EVP4_WHEAT
						TR:Q5MFQ1_WHEAT
						TR:Q5MFQ2_WHEAT
						TR:B2Y2Q1_WHEAT
						TR:B2Y2Q4_WHEAT
						TR:B2Y2Q3_WHEAT
						TR:Q8W3V6_WHEAT
						TR:B2Y2R5_WHEAT
						TR:B2Y2Q2_WHEAT
						TR:Q9ZNY0_WHEAT
						TR:Q8H0J4_WHEAT
						TR:O22108_WHEAT
						TR:F6M7E1_WHEAT
						TR:H9XH02_WHEAT
						TR:D2DII8_WHEAT
						TR:A4KZ73_WHEAT
						TR:H9XGZ8_WHEAT
						TR:Q52NZ4_WHEAT
						TR:Q8W3V8_WHEAT
						TR:Q8W3V9_WHEAT
						TR:D2DII5_WHEAT
						TR:D3U315_WHEAT
						TR:D2DII6_WHEAT
						TR:Q8GU18_WHEAT
						TR:Q41550_WHEAT

						TR:B5ANT3_WHEAT
				Triticum aestivum subsp. yunnanense	425487	TR:A8CA05_WHEAT
						TR:A9YSH4_WHEAT
				Triticum aestivum subsp. tibeticum	231718	TR:Q7Y075_WHEAT
				Triticum turgidum subsp. dicoccon	49225	TR:D5FPE5_TRITU
				Triticum turgidum	4571	TR:Q0Q2J0_TRITU
						TR:Q0Q2J1_TRITU
						TR:A7XDG0_TRITU
						TR:A7X9X7_TRITU
				Triticum turgidum subsp. durum	4567	TR:D5FPE4_TRIDB
						TR:O49958_TRIDB
						TR:Q9XGE9_TRIDB
						TR:Q9FEQ1_TRIDB
						TR:Q41603_TRIDB
				Triticum monococcum	4568	TR:B8XU58_TRIMO
						TR:B8XU59_TRIMO
						TR:I7G262_TRIMO
						TR:B8XU60_TRIMO
				Triticum dicoccoides	85692	TR:B8Y0L5_TRIDC
						TR:D5FPE0_TRIDC
						TR:B6UKK3_TRIDC
						TR:B6UKJ8_TRIDC
						TR:B6UKK0_TRIDC
						TR:B6UKK1_TRIDC
						TR:B6UKK4_TRIDC
						TR:B6UKK6_TRIDC
						TR:B6UKK9_TRIDC
						TR:B6UKL0_TRIDC
						TR:A2IBJ9_TRIDC
						TR:D5FPE7_TRIDC
				Triticum zhukovskyi	77602	TR:A2IBK1_9POAL
						TR:A2IBK1_9POAL
				Triticum aestivum/Thinopyrum intermedium	218488	TR:Q84U13_9POAL
				Leymus mollis	183794	TR:C6K8R5_9POAL
						TR:F5A7G7_9POAL
						TR:F5A7G8_9POAL
						TR:F5A7G6_9POAL
				Secale sylvestre	4552	TR:Q5MGR3_9POAL
						TR:Q5MGR4_9POAL
				Triticum polonicum	77606	TR:A2TN60_9POAL
				Thinopyrum intermedium	85679	TR:E5FEZ7_9POAL
						TR:Q84U17_9POAL
				Dasypyrum villosum	40247	TR:A9Z0K7_9POAL
						TR:F6KHQ1_9POAL
						TR:J9QGN7_9POAL

						TR:F8S8T4_9POAL
						TR:F8S8T7_9POAL
						TR:F8S8U3_9POAL
						TR:F8S8U4_9POAL
						TR:F8S8U6_9POAL
						TR:J9QFU9_9POAL
						TR:J9QGY5_9POAL
						TR:F6KHQ0_9POAL
						TR:F8S8T5_9POAL
						TR:F8S8T6_9POAL
						TR:F8S8T8_9POAL
						TR:F8S8T9_9POAL
						TR:F8S8U0_9POAL
						TR:F8S8U1_9POAL
						TR:F8S8U2_9POAL
						TR:F8S8U5_9POAL
						TR:F8S8U7_9POAL
						TR:J9QFV1_9POAL
						TR:J9QEK9_9POAL
						TR:J9QFG0_9POAL
						TR:A9Z0J9_9POAL
						TR:A9Z0K1_9POAL
						TR:A9Z0K4_9POAL
						TR:A9Z0K2_9POAL
						TR:A9Z0K5_9POAL
						TR:A9Z0K6_9POAL
						TR:A9Z0K8_9POAL
						TR:A9Z0K3_9POAL
				Dasypyrum hordeaceum	49449	TR:A8VZH1_9POAL
						TR:A8VZI4_9POAL
						TR:A9Z0L4_9POAL
						TR:A8VZF6_9POAL
						TR:D1MI66_9POAL
				Thinopyrum elongatum	4588	TR:A7LHA8_LOPEL
						TR:Q5XY00_LOPEL
				Hordeum vulgare	4513	SP:HOG3_HORVU
						TR:I6TEV2_HORVU
				Aegilops longissima	4486	TR:A9YWM3_AEGLO
						TR:D5FPF2_AEGLO
						TR:D5FPF0_AEGLO
						TR:C8CCM4_AEGLO
						TR:A2IBJ8_AEGLO
						TR:A9YWM2_AEGLO
						TR:A9YWM7_AEGLO
				Aegilops tauschii	37682	TR:Q5MB98_AEGTA

						TR:D3U335_AEGTA
						TR:Q6J6U9_AEGTA
						TR:B6UKJ3_AEGTA
						TR:B6UKJ4_AEGTA
						TR:B6UKI7_AEGTA
						TR:B6UKI9_AEGTA
						TR:B6UKJ1_AEGTA
						TR:B6UKJ5_AEGTA
						TR:D3U330_AEGTA
						TR:Q5MB99_AEGTA
				Aegilops tauschii x Triticum turgidum	285950	TR:Q6B780_9POAL
				Thinopyrum ponticum x Triticum aestivum	222994	TR:A8IEA6_9POAL
				Aegilops markgrafii	4494	TR:C8CCM6_9POAL
				Psathyrostachys huashanica	37730	TR:B8Y444_9POAL
						TR:E3UMZ0_9POAL
						TR:E3UMZ1_9POAL
				Psathyrostachys juncea	4586	TR:G0YLZ5_PSAJU
						TR:G0YLZ4_PSAJU
						TR:G0YLZ0_PSAJU
						TR:G0YLZ1_PSAJU
				Aegilops speltoides	4573	TR:C8CCM8_AEGSP
				Aegilops triuncialis	39391	TR:Q5MB97_AEGTR
F-box associated interaction domain	IPR017451	6512	1	*Sorghum bicolor*	4558	TR:C5WR35_SORBI
ATP-dependent RNA helicase DEAD-box, conserved site	IPR000629	63977	2	*Arabidopsis thaliana*	3702	SP:RH40_ARATH
				Arabidopsis lyrata subsp. lyrata	81972	TR:D7L5I4_ARALL
DWNN domain	IPR014891	794	1	*Coccomyxa subellipsoidea C-169*	574566	TR:I0Z6X5_9CHLO
Zinc knuckle CX2CX3GHX4C	IPR025829	382	1	*Coccomyxa subellipsoidea C-169*	574566	TR:I0Z6X5_9CHLO
Protein of unknown function DUF3250	IPR021656	259	1	*Volvox carteri*	3067	TR:D8U185_VOLCA
DNA helicase, UvrD-like, C-terminal	IPR014017	74617	1	*Volvox carteri*	3067	TR:D8U185_VOLCA
Calcium/calmodulin-dependent/calcium-dependent protein kinase	IPR020636	10583	1	*Volvox carteri*	3067	TR:D8UGB6_VOLCA
HARP domain	IPR010003		1	*Volvox carteri*	3067	TR:D8TLQ2_VOLCA
CHCH	IPR010625	1095	3	*Tetraodon nigroviridis*	99883	TR:Q4TG53_TETNG
						TR:Q4REM5_TETNG
						TR:H3C8B8_TETNG

DZF	IPR006561	578	1	*Tetraodon nigroviridis*	99883	TR:Q4RUC4_TETNG
Zinc finger, U1-type	IPR003604	3080	1	*Tetraodon nigroviridis*	99883	TR:Q4RUC4_TETNG
Helicase/SANT-associated, DNA binding	IPR014012	1065	5	*Vitis vinifera*	29760	TR:F6I025_VITVI
				Debaryomyces hansenii	284592	TR:Q6BJE1_DEBHA
				Caenorhabditis brenneri	135651	TR:G0M812_CAEBE
				Dictyostelium purpureum	5786	TR:F1A5J1_DICPU
				Dictyostelium discoideum	44689	TR:Q55GK2_DICDI
HSA	IPR006562	943	3	*Vitis vinifera*	29760	TR:F6I025_VITVI
				Candida parapsilosis	578454	TR:G8BH70_CANPC
				Dictyostelium purpureum	5786	TR:F1A5J1_DICPU
Myb-like domain	IPR017877	4255	4	*Vitis vinifera*	29760	TR:F6I025_VITVI
				Candida parapsilosis	578454	TR:G8BH70_CANPC
				Dictyostelium purpureum	5786	TR:F1A5J1_DICPU
				Dictyostelium discoideum	44689	TR:Q55GK2_DICDI
AUX/IAA protein	IPR003311	1935	1	*Cucumis sativus*	3659	TR:Q6L8U3_CUCSA
Aux/IAA-ARF-dimerisation	IPR011525	1942	1	*Cucumis sativus*	3659	TR:Q6L8U3_CUCSA
Auxin response factor	IPR010525	804	1	*Cucumis sativus*	3659	TR:Q6L8U3_CUCSA
B3 DNA binding domain	IPR003340	2168	1	*Cucumis sativus*	3659	TR:Q6L8U3_CUCSA
DNA-binding pseudobarrel domain	IPR015300	2609	1	*Cucumis sativus*	3659	TR:Q6L8U3_CUCSA
PWI domain	IPR002483	1166	1	*Yarrowia lipolytica*	284591	TR:Q6CDX5_YARLI
Transcription factor	IPR007219	12952	5	*Yarrowia lipolytica*	284591	TR:Q6CHB0_YARLI
				Spathaspora passalidarum	619300	TR:G3ATH7_SPAPN
				Hypocrea atroviridis	452589	TR:G9NL19_HYPAI
				Podospora anserina	515849	TR:B2ACE3_PODAN
				Verticillium alfalfae	526221	TR:C9SN42_VERA1
Zn(2)-C6 fungal-type DNA-binding domain	IPR001138	17917	17	*Yarrowia lipolytica*	284591	TR:Q6CHB0_YARLI
				Spathaspora passalidarum	619300	TR:G3APH2_SPAPN
				Hypocrea atroviridis	452589	TR:G9NL19_HYPAI
				Podospora anserina	515849	TR:B2ACE3_PODAN
				Verticillium alfalfae	526221	TR:C9SN42_VERA1
				Saccharomyces cerevisiae	764102	TR:E7Q716_YEASB
						SP:ECM22_YEAST
						TR:A7A1B5_YEAS7

						TR:B5VNE5_YEAS6
						TR:C7GT26_YEAS2
						TR:C8ZDJ2_YEAS8
						TR:E7LXR7_YEASV
						TR:E7NKS4_YEASO
						TR:E7QI59_YEASZ
						TR:H0GKF3_9SACH
						TR:G2WJ57_YEASK
						TR:E7KRV5_YEASL
RAM signalling pathway, SOG2	IPR019487	158	1	*Yarrowia lipolytica*	284591	TR:Q6C1M0_YARLI
Transcription factor, STE-like	IPR003120	210	1	*Yarrowia lipolytica*	284591	TR:Q6C5Q0_YARLI
Cyclin-like	IPR013763	11051	5	*Lodderomyces elongisporus*	379508	TR:A5DUV2_LODEL
				Arthroderma benhamiae	663331	TR:D4AIT9_ARTBC
				Trichophyton verrucosum	663202	TR:D4DFR8_TRIVH
				Trichophyton tonsurans	647933	TR:F2RYA1_TRIT1
				Trichophyton equinum	559882	TR:F2PRJ7_TRIEC
Cyclin, C-terminal domain	IPR004367	3414	1	*Lodderomyces elongisporus*	379508	TR:A5DUV2_LODEL
NDT80 DNA-binding domain	IPR024061	463	2	*Lodderomyces elongisporus*	379508	TR:A5H2N9_LODEL
				Spathaspora passalidarum	619300	TR:G3AKA5_SPAPN
DDE superfamily endonuclease, CENP-B-like	IPR004875	3316	3	*Lodderomyces elongisporus*	379508	TR:A5E1L5_LODEL
				Candida albicans	294748	TR:C4YMY3_CANAW
						TR:Q59NK7_CANAL
HTH CenpB-type DNA-binding domain	IPR006600	2593	2	*Lodderomyces elongisporus*	379508	TR:A5E1L5_LODEL
				Komagataella pastoris	981350	TR:F2QM85_PICP7
Ubiquitin system component Cue	IPR003892	2120	2	*Komagataella pastoris*	644223	TR:C4QW10_PICPG
				Saccharomyces cerevisiae	764097	TR:E7KEY3_YEASA
PSP1, C-terminal	IPR007557	2263	1	*Candida dubliniensis*	573826	TR:B9WJJ2_CANDC
RNA- binding protein Lupus La	IPR006630	1617	1	*Candida dubliniensis*	573826	TR:B9WKI8_CANDC
6-phosphogluconate dehydrogenase, C-terminal-like	IPR008927	78846	1	*Scheffersomyces stipitis*	322104	TR:A3GHQ4_PICST
Ketopantoate reductase ApbA/PanE, C-terminal	IPR013752	6448	1	*Scheffersomyces stipitis*	322104	TR:A3GHQ4_PICST
Dehydrogenase, multihelical	IPR013328	58539	1	*Scheffersomyces stipitis*	322104	TR:A3GHQ4_PICST

GRZYBY						
Activator of mitotic machinery Cdc14 phosphatase activation C-terminal	IPR013941	165	1	*Scheffersomyces stipitis*	322104	TR:A3LS86_PICST
ARID/BRIGHT DNA-binding domain	IPR001606	2042	5	*Naumovozyma castellii*	1064592	TR:G0VCN5_NAUCC
				Meyerozyma guilliermondii	294746	TR:A5DE07_PICGU
				Candida parapsilosis	578454	TR:G8BIK7_CANPC
				Trichosporon asahii var. asahii	1186058	TR:J6EXZ0_TRIAS
				Trichosporon asahii var. asahii	1220162	TR:K1WJ31_TRIAC
Transcription factor, MADS-box	IPR002100	6778	4	*Tetrapisispora blattae*	1071380	TR:I2GZF5_TETBL
				Kazachstania africana	1071382	TR:H2AN55_KAZAF
				Rhizopus delemar	246409	TR:I1BLN6_RHIO9
				Rhizopus delemar	246409	TR:I1BI10_RHIO9
LsmAD domain	IPR009604	472	4	*Hypocrea jecorina*	431241	TR:G0RDB0_HYPJQ
				Nectria haematococca	660122	TR:C7YHZ6_NECH7
				Tetrahymena thermophila	312017	TR:Q23QL4_TETTS
				Salpingoeca rosetta	946362	TR:F2UB05_SALS5
AAA+ ATPase domain	IPR003593	657571	2	*Hypocrea jecorina*	431241	TR:G0RGX3_HYPJQ
				Saccharomonospora viridis	471857	TR:C7MXV2_SACVD
ATPase, AAA-type, core	IPR003959	62855	1	*Hypocrea jecorina*	431241	TR:G0RGX3_HYPJQ
Mediator complex, subunit Med15, fungi	IPR008626	99	1	*Rhizopus delemar*	246409	TR:I1BJ65_RHIO9
Protein of unknown function DUF1752, fungi	IPR013860	541	1	*Rhizopus delemar*	246409	TR:I1CHV5_RHIO9
Zinc finger, GATA-type	IPR000679	3775	3	*Rhizopus delemar*	246409	TR:I1CHV5_RHIO9
				Dictyostelium discoideum	44689	SP:GTAG_DICDI
				Salpingoeca rosetta	946362	TR:F2TY54_SALS5
Fungal chitin synthase	IPR004835	1505	1	*Fibroporia radiculosa*	1078123	TR:J4G782_9APHY
SNF5/SMARCB1/INI1	IPR006939	546	3	*Cryptococcus neoformans var. neoformans*	283643	TR:F5HD71_CRYNB
						TR:Q5KNA4_CRYNJ
				Cryptococcus gattii	367775	TR:E6QYJ3_CRYGW
Chitin synthesis regulation, Congo red resistance, RCR protein	IPR020999	333	4	*Arthroderma otae*	554155	TR:C5G0Y8_ARTOC
				Trichophyton tonsurans	647933	TR:F2S4E4_TRIT1
				Trichophyton equinum	559882	TR:F2PUE2_TRIEC
				Trichophyton rubrum	559305	TR:F2SKR7_TRIRC
C4-dicarboxylate transporter/malic acid	IPR004695	3750	1	*Magnaporthe oryzae*	242507	TR:G4MY83_MAGO7

transport protein						
Arf GTPase activating protein	IPR001164	3825	2	*Phaeosphaeria nodorum*	321614	TR:Q0UXI8_PHANO
				Eimeria tenella	5802	TR:C8TDR1_EIMTE
Ubiquitin-associated/translation elongation factor EF1B, N-terminal, eukaryote	IPR015940	6732	2	*Phaeosphaeria nodorum*	321614	TR:Q0UXI8_PHANO
				Ichthyophthirius multifiliis	857967	TR:G0R1R9_ICHMG
Uncharacterised protein family Cys-rich	IPR006461	1339	1	*Pyrenophora teres f. teres*	861557	TR:E3SA10_PYRTT
Nuclear abundant poly(A) RNA-binding protein 2, Nab2	IPR021083	31	1	*Kazachstania naganishii*	1071383	TR:J7S062_KAZNA
Zinc finger, BED-type predicted	IPR003656	1939	1	*Macrophomina phaseolina*	1126212	TR:K2S7S7_MACPH
Transcription factor Aft1, osmotic stress domain	IPR020956	144	2	*Wickerhamomyces ciferrii*	1206466	TR:K0KJU9_9ASCO
				Candida tropicalis	294747	TR:C5ME60_CANTT
Sel1-like	IPR006597	15768	2	*Wickerhamomyces ciferrii*	1206466	TR:K0KUU0_9ASCO
				Botryotinia fuckeliana	999810	TR:G2XW17_BOTF4
Tetratricopeptide-like helical	IPR011990	180074	4	*Wickerhamomyces ciferrii*	1206466	TR:K0KUU0_9ASCO
				Botryotinia fuckeliana	999810	TR:G2XW17_BOTF4
				Burkholderia phytofirmans	398527	TR:B2TFR9_BURPP
				Dictyostelium purpureum	5786	TR:F0ZYQ8_DICPU
Subunit of cleavage factor IA Pcf11	IPR021605	59	1	*Wickerhamomyces ciferrii*	1206466	TR:K0KL65_9ASCO
ENTH/VHS	IPR008942	6361	7	*Wickerhamomyces ciferrii*	1206466	TR:K0KL65_9ASCO
				Meyerozyma guilliermondii	294746	TR:A5DGW4_PICGU
				Zygosaccharomyces rouxii	559307	TR:C5DYJ4_ZYGRC
				Verticillium dahliae	498257	TR:G2X9K9_VERDV
				Verticillium alfalfae	526221	TR:C9SQG8_VERA1
				Entamoeba dispar	370354	TR:B0EU18_ENTDS
				Oxytricha trifallax	1172189	TR:J9I8U5_9SPIT
CID domain	IPR006569	2116	2	*Wickerhamomyces ciferrii*	1206466	TR:K0KL65_9ASCO
				Entamoeba dispar	370354	TR:B0EU18_ENTDS
LisH dimerisation motif, subgroup	IPR013720	1567	2	*Pichia sorbitophila*	559304	TR:G8YRJ1_PICSO
				Lachancea thermotolerans	559295	TR:C5DE52_LACTC
LisH dimerisation motif	IPR006594	4924	3	*Pichia sorbitophila*	559304	TR:G8YRJ1_PICSO
				Lachancea thermotolerans	559295	TR:C5DE52_LACTC
				Tetrahymena thermophila	312017	TR:Q231Q2_TETTS

Transcription initiation factor TFIID	IPR003228	420	3	*Chaetomium thermophilum*	759272	TR:G0S2A1_CHATD
				Grosmannia clavigera	655863	TR:F0XN84_GROCL
				Dictyostelium discoideum	44689	SP:TAF12_DICDI
Heat shock protein 70 family	IPR013126	24709	1	*Gaeumannomyces graminis var. tritici*	644352	TR:J3PE17_GAGT3
Glycoside hydrolase, catalytic domain	IPR013781	118272	1	*Gaeumannomyces graminis var. tritici*	644352	TR:J3NUQ4_GAGT3
Glycoside hydrolase, superfamily	IPR017853	148383	1	*Gaeumannomyces graminis var. tritici*	644352	TR:J3NUQ4_GAGT3
Rab-GTPase-TBC domain	IPR000195	8059	1	*Gaeumannomyces graminis var. tritici*	644352	TR:J3NNY7_GAGT3
AGC-kinase, C-terminal	IPR000961	8598	2	*Gaeumannomyces graminis var. tritici*	644352	TR:J3P6H6_GAGT3
				Trichinella spiralis	6334	TR:E5S526_TRISP
HR1 rho-binding repeat	IPR011072	986	1	*Gaeumannomyces graminis var. tritici*	644352	TR:J3P6H6_GAGT3
Protein kinase, C-terminal	IPR017892	4478	2	*Gaeumannomyces graminis var. tritici*	644352	TR:J3P6H6_GAGT3
				Trichinella spiralis	6334	TR:E5S526_TRISP
Protein kinase C-like, phorbol ester/diacylglycerol binding	IPR002219	6999	2	*Gaeumannomyces graminis var. tritici*	644352	TR:J3P6H6_GAGT3
				Trichinella spiralis	6334	TR:E5S526_TRISP
Transcription factor Spt20	IPR021950	359	6	*Clavispora lusitaniae*	306902	TR:C4Y1X6_CLAL4
				Ajellomyces dermatitidis	559298	TR:C5JYC6_AJEDS
						TR:C5GM52_AJEDR
				Paracoccidioides brasiliensis	502780	TR:C1GH85_PARBD
				Paracoccidioides lutzii	502779	TR:C1H3C8_PARBA
				Paracoccidioides brasiliensis	482561	TR:C0SEI1_PARBP
Ubiquitin-associated domain	IPR013896	157	1	*Clavispora lusitaniae*	306902	TR:C4Y9B9_CLAL4
AP180 N-terminal homology (ANTH) domain	IPR011417	1306	**4**	*Meyerozyma guilliermondii*	294746	TR:A5DGW4_PICGU
				Zygosaccharomyces rouxii	559307	TR:C5DYJ4_ZYGRC
				Verticillium dahliae	498257	TR:G2X9K9_VERDV
						TR:C9SQG8_VERA1
Clathrin adaptor, phosphoinositide-binding, GAT-like	IPR014712	821	4	*Meyerozyma guilliermondii*	294746	TR:A5DGW4_PICGU
				Zygosaccharomyces rouxii	559307	TR:C5DYJ4_ZYGRC
				Verticillium dahliae	498257	TR:G2X9K9_VERDV
						TR:C9SQG8_VERA1
Epsin-like, N-terminal	IPR013809	2434	5	*Meyerozyma guilliermondii*	294746	TR:A5DGW4_PICGU
				Zygosaccharomyces rouxii	559307	TR:C5DYJ4_ZYGRC

				Verticillium dahliae	498257	TR:G2X9K9_VERDV
						TR:C9SQG8_VERA1
				Oxytricha trifallax	1172189	TR:J9I8U5_9SPIT
Glutamine-Leucine-Glutamine	IPR014978	673	1	*Debaryomyces hansenii*	284592	TR:Q6BJE1_DEBHA
Like-Sm (LSM) domain	IPR010920	29772	1	*Emericella nidulans*	227321	TR:Q5B077_EMENI
Mechanosensitive ion channel MscS-like, plants/fungi	IPR016688	181	1	*Emericella nidulans*	227321	TR:Q5B077_EMENI
Mechanosensitive ion channel MscS	IPR006685	19061	1	*Emericella nidulans*	227321	TR:Q5B077_EMENI
Domain of unknown function DUF1720	IPR013182	117	1	*Colletotrichum graminicola*	645133	TR:E3QDI8_COLGM
SLA1 homology domain 1, SHD1	IPR007131	197	1	*Colletotrichum graminicola*	645133	TR:E3QDI8_COLGM
AT hook, DNA-binding motif	IPR017956	7441	2	*Neurospora crassa*	367110	TR:Q7S0G6_NEUCR
				Dictyostelium purpureum	5786	TR:F0ZB68_DICPU
Zinc finger, MIZ-type	IPR004181	1329	2	*Neurospora crassa*	367110	TR:Q7S0G6_NEUCR
				Candida albicans	237561	TR:Q5A8Y9_CANAL
Cyclin PHO80-like	IPR013922	1585	5	*Arthroderma benhamiae*	663331	TR:D4AIT9_ARTBC
				Trichophyton verrucosum	663202	TR:D4DFR8_TRIVH
				Trichophyton tonsurans	647933	TR:F2RYA1_TRIT1
				Trichophyton equinum	559882	TR:F2PRJ7_TRIEC
				Lachancea thermotolerans	559295	TR:C5DLP2_LACTC
Neutrophil cytosol factor 2 p67phox	IPR000108	2328	1	*Kluyveromyces lactis*	284590	TR:Q6CPM1_KLULA
Domain of unknown function DUF1771	IPR013899	579	2	*Penicillium marneffei*	441960	TR:B6QCM0_PENMQ
				Aspergillus kawachii	1033177	TR:G7XNI2_ASPKW
Smr protein/MutS2 C-terminal	IPR002625	7475	2	*Penicillium marneffei*	441960	TR:B6QCM0_PENMQ
				Aspergillus kawachii	1033177	TR:G7XNI2_ASPKW
Steadiness box	IPR017916	411	1	*Pyrenophora tritici-repentis*	426418	TR:B2WIB9_PYRTR
Ubiquitin-conjugating enzyme/RWD-like	IPR016135	13589	1	*Pyrenophora tritici-repentis*	426418	TR:B2WIB9_PYRTR
Ubiquitin E2 variant, N-terminal	IPR008883	525	1	*Pyrenophora tritici-repentis*	426418	TR:B2WIB9_PYRTR
5-AMP-activated protein kinase, beta subunit, interaction domain	IPR006828	591	1	*Naumovozyma dairenensis*	1071378	TR:G0W772_NAUDC

Zinc finger, CCCH-type	IPR000571	12071	2	*Naumovozyma dairenensis*	1071378	TR:G0WAM9_NAUDC
				Ajellomyces dermatitidis	559298	TR:C5JW14_AJEDS
Bromo adjacent homology (BAH) domain	IPR001025	2315	1	*Vanderwaltozyma polyspora*	436907	TR:A7TQI4_VANPO
von Willebrand factor, type A	IPR002035	31980	4	*Ajellomyces capsulatus*	544712	TR:C6HR06_AJECH
				Ajellomyces dermatitidis	559297	TR:C5GNZ8_AJEDR
				Ajellomyces dermatitidis	653446	TR:F2TDT9_AJEDA
				Naegleria gruberi	5762	TR:D2VCR4_NAEGR
Anp1	IPR005109	473	1	*Zygosaccharomyces rouxii*	559307	TR:C5DXH7_ZYGRC
Glycosyl transferase, family 8	IPR002495	7788	1	*Chaetomium globosum*	306901	TR:Q2GW94_CHAGB
Cohesin loading factor	IPR019440	262	1	*Fusarium pseudograminearum*	1028729	TR:K3W1C3_FUSPC
CYTH-like domain	IPR023577	5948	2	*Verticillium dahliae*	498257	TR:G2X9B0_VERDV
				Hypocrea virens	413071	TR:G9N2J5_HYPVG
mRNA capping enzyme, beta subunit, structural domain	IPR004206	309	2	*Verticillium dahliae*	498257	TR:G2X9B0_VERDV
				Hypocrea virens	413071	TR:G9N2J5_HYPVG
DNA/RNA non-specific endonuclease, active site	IPR018524	1398	2	*Aspergillus niger*	425011	TR:A2QZ96_ASPNC
						TR:G3XVE5_ASPNA
DNA/RNA non-specific endonuclease	IPR001604	3452	2	*Aspergillus niger*	425011	TR:A2QZ96_ASPNC
						TR:G3XVE5_ASPNA
Extracellular Endonuclease, subunit A	IPR020821	2700	2	*Aspergillus niger*	425011	TR:A2QZ96_ASPNC
						TR:G3XVE5_ASPNA
PINIT domain	IPR023321	542	1	*Candida albicans*	237561	TR:Q5A8Y9_CANAL
CCR4-Not complex component, Not1, C-terminal	IPR007196	471	1	*Candida tropicalis*	294747	TR:C5M8N8_CANTT
CCR4-Not complex, Not1 subunit, domain of unknown function DUF3819	IPR024557	437	1	*Candida tropicalis*	294747	TR:C5M8N8_CANTT
TORC1 complex, subunit TCO89	IPR018857	61	1	*Candida tropicalis*	294747	TR:C5MEJ3_CANTT
Ovarian tumour, otubain	IPR003323	3211	2	*Arthroderma gypseum*	535722	TR:E4UWP5_ARTGP
				Paramecium tetraurelia	5888	TR:A0BX13_PARTE
Peptidase C65, otubain	IPR019400	512	2	*Arthroderma gypseum*	535722	TR:E4UWP5_ARTGP

				Paramecium tetraurelia	5888	TR:A0BX13_PARTE
Dcp1-like decapping	IPR010334	478	1	*Puccinia triticina*	630390	TR:J3QDM0_PUCT1
Myb domain	IPR017930	11779	1	*Cordyceps militaris*	983644	TR:G3JEU7_CORMM
Telomere repeat-binding factor, dimerisation domain	IPR013867	298	1	*Cordyceps militaris*	983644	TR:G3JEU7_CORMM
Kelch-type beta propeller	IPR015915	16973	2	*Eremothecium cymbalariae*	931890	TR:G8JP18_ERECY
				Chlorella variabilis	554065	TR:E1ZPR5_CHLVA
Kelch repeat type 1	IPR006652	9098	1	*Eremothecium cymbalariae*	931890	TR:G8JP18_ERECY
Protein of unknown function DUF3468	IPR021858	4243	12	*Saccharomyces cerevisiae*	764102	TR:E7Q716_YEASB
					559292	SP:ECM22_YEAST
					307796	TR:A7A1B5_YEAS7
					545124	TR:B5VNE5_YEAS6
					574961	TR:C7GT26_YEAS2
					643680	TR:C8ZDJ2_YEAS8
					764099	TR:E7LXR7_YEASV
					764101	TR:E7NKS4_YEASO
					764100	TR:E7QI59_YEASZ
					1095631	TR:H0GKF3_9SACH
					721032	TR:G2WJ57_YEASK
					764098	TR:E7KRV5_YEASL
G-protein beta WD-40 repeat	IPR020472	21634	15	*Saccharomyces cerevisiae*	764102	TR:E7Q1H5_YEASB
					559292	SP:TUP1_YEAST
					307796	TR:A6ZTQ6_YEAS7
					285006	TR:B3LUD8_YEAS1
					545124	TR:B5VF12_YEAS6
					574961	TR:C7GUW4_YEAS2
					643680	TR:C8Z4H1_YEAS8
					764101	TR:E7NFE6_YEASO
					764100	TR:E7QC78_YEASZ
					1095631	TR:H0GD95_9SACH
					721032	TR:G2WA92_YEASK
					764097	TR:E7KA75_YEASA
				Saccharomyces arboricola	1160507	TR:J8Q781_SACAR
				Paramecium tetraurelia	5888	TR:A0CXK4_PARTE
				Paramecium tetraurelia	5888	TR:A0CYP9_PARTE
Transcriptional repressor Tup1, N-terminal	IPR013890	204	13	*Saccharomyces cerevisiae*	764102	TR:E7Q1H5_YEASB
					559292	SP:TUP1_YEAST

					307796	TR:A6ZTQ6_YEAS7
					285006	TR:B3LUD8_YEAS1
					545124	TR:B5VF12_YEAS6
					574961	TR:C7GUW4_YEAS2
					643680	TR:C8Z4H1_YEAS8
					764101	TR:E7NFE6_YEASO
					764100	TR:E7QC78_YEASZ
					1095631	TR:H0GD95_9SACH
					721032	TR:G2WA92_YEASK
					764097	TR:E7KA75_YEASA
				Saccharomyces arboricola	1160507	TR:J8Q781_SACAR
Quinonprotein alcohol dehydrogenase-like-superfamily	IPR011047	15130	1	*Kitasatospora setae*	452652	TR:E4N3L6_KITSK
Collagen-binding surface protein Cna-like, B-type domain	IPR008454	6667	1	*Kitasatospora setae*	452652	TR:E4N1V7_KITSK
LPXTG-motif cell wall anchor	IPR019931	22958	1	*Kitasatospora setae*	452652	TR:E4N1V7_KITSK
Twin-arginine translocation pathway, signal sequence	IPR006311	73771	3	*Kitasatospora setae*	452652	TR:E4N1V7_KITSK
				Pseudomonas chlororaphis	1037915	TR:I4XT35_9PSED
						TR:J2YIM7_9PSED
Uncharacterised protein family UPF0755, YceG-like	IPR003770	5547	4	*Kitasatospora setae*	452652	TR:E4N7R6_KITSK
				Streptomyces sp. W007	1055352	TR:H0BMT4_9ACTO
				Streptomyces griseus	649189	TR:G0Q9Q0_STRGR
				Streptomyces griseus subsp. griseus	455632	TR:B1W448_STRGG
Polyadenylate binding protein, human types 1, 2, 3, 4	IPR006515	711	1	*Capsaspora owczarzaki*	595528	TR:E9C1R9_CAPO3
Sterile alpha motif domain	IPR001660	8644	1	*Capsaspora owczarzaki*	595528	TR:E9BWQ2_CAPO3
Sterile alpha motif/pointed domain	IPR013761	11290	1	*Capsaspora owczarzaki*	595528	TR:E9BWQ2_CAPO3
Sterile alpha motif, type 2	IPR011510	3760	1	*Capsaspora owczarzaki*	595528	TR:E9BWQ2_CAPO3
DNA/RNA helicase, DEAD/DEAH box type, N-terminal	IPR011545	80893	4	*Arabidopsis thaliana*	3702	SP:RH40_ARATH
				Arabidopsis lyrata subsp. lyrata	81972	TR:D7L5I4_ARALL
				Ferrimonas balearica	550540	TR:E1SVZ1_FERBD
				Paramecium tetraurelia	5888	TR:A0BWN9_PARTE
Domain of unknown function DUF3854	IPR024385	289	1	*Nostoc sp.*	103690	TR:Q8YKS6_NOSS1

Domain of unknown function DUF4149	IPR025423	1238	1	*Neisseria shayeganii*	1032488	TR:G4CEQ1_9NEIS
Fimbrial assembly PilN	IPR007813	2315	1	*Idiomarina xiamenensis*	740709	TR:K2JPH4_9GAMM
Lactobacillus phage LBR48, DUF805	IPR008523	4961	1	*Arthrobacter globiformis*	1077972	TR:H0QLI2_ARTGO
ATPase, AAA-3	IPR011703	5429	1	*Nocardia brasiliensis*	1133849	TR:K0F1A7_9NOCA
Domain of unknown function DUF4333	IPR025637	396	1	*Mycobacterium colombiense*	1041522	TR:J5EM93_9MYCO
Integrating conjugative element protein, PFL4697-type	IPR022266	549	3	*Erwinia amylovora*	1027397	TR:G8LQK2_ERWAM
				Erwinia amylovora	1027397	TR:I1VYP7_ERWAM
				Erwinia billingiae	634500	TR:D8MJF0_ERWBE
Bax inhibitor-1	IPR010539	957	2	*Micromonospora aurantiaca*	644283	TR:D9TB19_MICAI
				Micromonospora sp.	648999	TR:E8S0K6_MICSL
Outer membrane protein, bacterial	IPR006664	14910	1	*Methylomonas methanica*	857087	TR:G0A7E8_METMM
Outer membrane protein, conserved site	IPR006690	6247	1	*Methylomonas methanica*	857087	TR:G0A7E8_METMM
Outer membrane protein, C-terminal	IPR006665	25722	1	*Methylomonas methanica*	857087	TR:G0A7E8_METMM
SNARE associated Golgi protein	IPR015414	22314	1	*Streptomyces hygroscopicus subsp. jinggangensis*	1133850	TR:H2JM60_STRHJ
ABC transporter-like	IPR003439	422549	1	*Saccharomonospora viridis*	471857	TR:C7MXV2_SACVD
ABC transporter, conserved site	IPR017871	348002	1	*Saccharomonospora viridis*	471857	TR:C7MXV2_SACVD
CheC-like protein	IPR007597	1466	1	*Clostridium clariflavum*	720554	TR:G8LVG6_CLOCD
Flagellar motor switch FliN	IPR012826	3186	1	*Clostridium clariflavum*	720554	TR:G8LVG6_CLOCD
Flagellar motor switch FliN/Type III secretion HrcQb	IPR001172	4473	1	*Clostridium clariflavum*	720554	TR:G8LVG6_CLOCD
Surface presentation of antigen (SpoA)	IPR001543	8751	1	*Clostridium clariflavum*	720554	TR:G8LVG6_CLOCD
Beta-lactamase /transpeptidase-like	IPR012338	79727	1	*Saccharophagus degradans*	203122	TR:Q21N62_SACD2
Glycosyl transferase, family 51	IPR001264	17316	1	*Saccharophagus degradans*	203122	TR:Q21N62_SACD2
Penicillin-binding protein 1C	IPR011815	1648	1	*Saccharophagus degradans*	203122	TR:Q21N62_SACD2

Penicillin-binding, C-terminal	IPR009647	1745	1	*Saccharophagus degradans*	203122	TR:Q21N62_SACD2
Penicillin-binding protein, transpeptidase	IPR001460	33574	1	*Saccharophagus degradans*	203122	TR:Q21N62_SACD2
Cellulose synthase operon C, C-terminal	IPR008410	1093	1	*Burkholderia phytofirmans*	398527	TR:B2TFR9_BURPP
Cellulose synthase, subunit C	IPR003921	269	1	*Burkholderia phytofirmans*	398527	TR:B2TFR9_BURPP
Tetratricopeptide repeat-containing domain	IPR013026	88871	2	*Burkholderia phytofirmans*	398527	TR:B2TFR9_BURPP
				Dictyostelium purpureum	5786	TR:F0ZYQ8_DICPU
Tetratricopeptide repeat	IPR019734	81297	2	*Burkholderia phytofirmans*	398527	TR:B2TFR9_BURPP
				Dictyostelium purpureum	5786	TR:F0ZYQ8_DICPU
LemA	IPR007156	3765	1	*Xanthomonas vesicatoria*	925775	TR:F0BH19_9XANT
LemA-like domain	IPR023353	3912	1	*Xanthomonas vesicatoria*	925775	TR:F0BH19_9XANT
NYN domain, limkain-b1-type	IPR021139	3904	1	*Hyphomicrobium denitrificans*	582899	TR:D8JU17_HYPDA
Mycobacterial pentapeptide repeat	IPR002989	1059	1	*Nocardia farcinica*	247156	TR:Q5Z1N9_NOCFA
Phosphoesterase	IPR007312	2531	3	*Pseudomonas chlororaphis*	1037915	TR:J2MZD3_9PSED
						TR:I4XT35_9PSED
						TR:J2YIM7_9PSED
Phospholipase C, bacterial, C-terminal	IPR008475	772	3	*Pseudomonas chlororaphis*	1037915	TR:J2MZD3_9PSED
						TR:I4XT35_9PSED
						TR:J2YIM7_9PSED
Phospholipase C, phosphocholine-specific	IPR017767	719	3	*Pseudomonas chlororaphis*	1037915	TR:J2MZD3_9PSED
						TR:I4XT35_9PSED
						TR:J2YIM7_9PSED
Flagellar hook-length control protein-like, C-terminal	IPR021136	3875	1	*Idiomarina loihiensis*	283942	TR:Q5QZP6_IDILO
Protein of unknown function DUF459	IPR007407	216	1	*Xanthobacter autotrophicus*	78245	TR:A7IGS6_XANP2
SGNH hydrolase-type esterase domain	IPR013830	95206	1	*Xanthobacter autotrophicus*	78245	TR:A7IGS6_XANP2
SGNH hydrolase-type esterase domain	IPR013831	25619	1	*Xanthobacter autotrophicus*	78245	TR:A7IGS6_XANP2
Metallopeptidase, catalytic domain	IPR024079	30369	1	*Moumouvirus Monve*	1128131	TR:H2EF10_9VIRU

Peptidase M43, pregnancy-associated plasma-A	IPR008754	707	1	*Moumouvirus Monve*	1128131	TR:H2EF10_9VIRU
Oxoglutarate / iron-dependent dioxygenase	IPR005123	18771	1	*Pristionchus pacificus*	54126	TR:H3F7D7_PRIPA
Prolyl 4-hydroxylase, alpha subunit	IPR006620	6062	1	*Pristionchus pacificus*	54126	TR:H3F7D7_PRIPA
Domain of unknown function DB	IPR002602	276	1	*Pristionchus pacificus*	54126	TR:H3DZM1_PRIPA
Potential DNA-binding domain	IPR025927	307	3	*Wuchereria bancrofti*	6293	TR:J9F0P6_WUCBA
				Brugia malayi	6279	TR:A8QG91_BRUMA
				Dictyostelium purpureum	5786	TR:F1A3Z1_DICPU
Serine/threonine-protein kinase OSR1/WNK, CCT domain	IPR024678	494	1	*Wuchereria bancrofti*	6293	TR:J9EPD0_WUCBA
Protein of unknown function DUF1136	IPR010939	129	1	*Ascaris suum*	6253	TR:F1KQG0_ASCSU
CTF transcription factor/nuclear factor 1	IPR000647	562	1	*Caenorhabditis remanei*	31234	TR:E3LQ82_CAERE
CTF transcription factor/nuclear factor 1, DNA-binding domain	IPR020604	494	1	*Caenorhabditis remanei*	31234	TR:E3LQ82_CAERE
CTF transcription factor/nuclear factor 1, N-terminal	IPR019548	456	1	*Caenorhabditis remanei*	31234	TR:E3LQ82_CAERE
	IPR003619		1	*Caenorhabditis remanei*	31234	TR:E3LQ82_CAERE
C-5 cytosine methyltransferase	IPR001525	8738	1	*Caenorhabditis remanei*	31234	TR:E3MFU8_CAERE
Zinc finger, CXXC-type	IPR002857	953	1	*Caenorhabditis remanei*	31234	TR:E3MFU8_CAERE
Domain of unknown function DUF148	IPR003677	266	1	*Caenorhabditis briggsae*	6238	TR:A8X193_CAEBR
Citron -like	IPR001180	2260	1	*Trichinella spiralis*	6334	TR:E5S526_TRISP
Myotonic dystrophy protein kinase, coiled coil	IPR014930	320	1	*Trichinella spiralis*	6334	TR:E5S526_TRISP
Serine/threonine-protein kinase MRCK	IPR026611	351	1	*Trichinella spiralis*	6334	TR:E5S526_TRISP
CCR4-NOT complex, subunit 3/ 5	IPR012270	334	1	*Loa loa*	7209	TR:J0DW06_LOALO
NOT2/NOT3/NOT5	IPR007282	926	1	*Loa loa*	7209	TR:J0DW06_LOALO
Not CCR4-Not complex component, N-terminal	IPR007207	441	1	*Loa loa*	7209	TR:J0DW06_LOALO

VHS	IPR002014	2099	1	*Entamoeba dispar*	370354	TR:B0EU18_ENTDS
Dbl homology (DH) domain	IPR000219	8248	2	*Entamoeba dispar*	370354	TR:B0EAI1_ENTDS
						TR:B0EP22_ENTDS
Serine-threonine/tyrosine-protein kinase catalytic domain	IPR001245	27727	1	*Polysphondylium pallidum*	13642	TR:D3B3V6_POLPA
Kinesin, motor region, conserved site	IPR019821	7503	1	*Polysphondylium pallidum*	13642	TR:D3BBQ6_POLPA
Kinesin, motor domain	IPR001752	10590	1	*Polysphondylium pallidum*	13642	TR:D3BBQ6_POLPA
Adenylyl cyclase class-3/4/guanylyl cyclase	IPR001054	71551	1	*Polysphondylium pallidum*	13642	TR:D3BFN2_POLPA
Adenylyl cyclase class-3/4/guanylyl cyclase, conserved site	IPR018297	3012	1		13642	TR:D3BFN2_POLPA
CHASE	IPR006189	1636	1	*Polysphondylium pallidum*	13642	TR:D3BFN2_POLPA
EPS15 homology (EH)	IPR000261	2081	1	*Dictyostelium fasciculatum*	1054147	TR:F4Q4C0_DICFS
			1	*Phaeodactylum tricornutum*	556484	TR:B7FRA9_PHATC
DNA repair protein Sae2/CtIP	IPR013882	244	1	*Dictyostelium fasciculatum*	1054147	TR:F4Q497_DICFS
Vacuolar protein sorting-associated	IPR007143	436	1	*Dictyostelium fasciculatum*	1054147	TR:F4PUM6_DICFS
Vacuolar protein sorting-associated, VPS28, C-terminal	IPR017899	368	1	*Dictyostelium fasciculatum*	1054147	TR:F4PUM6_DICFS
Vacuolar protein sorting-associated, VPS28, N-terminal	IPR017898	370	1	*Dictyostelium fasciculatum*	1054147	TR:F4PUM6_DICFS
Cell division protein FtsZ	IPR000158	7980	1	*Dictyostelium fasciculatum*	1054147	TR:F4QDY7_DICFS
Cell division protein FtsZ, conserved site	IPR020805	7056	1	*Dictyostelium fasciculatum*	1054147	TR:F4QDY7_DICFS
Cell division protein FtsZ, C-terminal	IPR024757	7353	1	*Dictyostelium fasciculatum*	1054147	TR:F4QDY7_DICFS
Tubulin/FtsZ, C-terminal	IPR008280	18645	1	*Dictyostelium fasciculatum*	1054147	TR:F4QDY7_DICFS
Tubulin/FtsZ, 2-layer sandwich domain	IPR018316	18326	1	*Dictyostelium fasciculatum*	1054147	TR:F4QDY7_DICFS
Tubulin/FtsZ, GTPase domain	IPR003008	25809	1	*Dictyostelium fasciculatum*	1054147	TR:F4QDY7_DICFS

Argonaute / Dicer protein, PAZ	IPR003100	2705	1	*Dictyostelium fasciculatum*	1054147	TR:F4PME8_DICFS
Stem cell self - renewal protein Piwi	IPR003165	2451	1	*Dictyostelium fasciculatum*	1054147	TR:F4PME8_DICFS
Ribonuclease H-like domain	IPR012337	174851	1	*Dictyostelium fasciculatum*	1054147	TR:F4PME8_DICFS
AT hook -like	IPR020478	957	1	*Dictyostelium purpureum*	5786	TR:F0ZB68_DICPU
High mobility group	IPR000116	299	1	*Dictyostelium purpureum*	5786	TR:F0ZB68_DICPU
Domain of unknown function DUF4062	IPR025139	510	1	*Dictyostelium purpureum*	5786	TR:F0ZYQ8_DICPU
SAC3/GANP/Nin1/mts3/eIF-3 p25	IPR005062	1273	1	*Dictyostelium purpureum*	5786	TR:F0ZXV1_DICPU
Adaptor protein complex AP-2, alpha subunit	IPR017104	411	1	*Dictyostelium purpureum*	5786	TR:F0ZS77_DICPU
Clathrin / coatomer adaptor, adaptin-like, N-terminal	IPR002553	4234	2	*Dictyostelium purpureum*	5786	TR:F0ZS77_DICPU
				Paramecium tetraurelia	5888	TR:A0CBT3_PARTE
Clathrin adaptor, alpha-adaptin, appendage, Ig-like subdomain	IPR013038	494	1	*Dictyostelium purpureum*	5786	TR:F0ZS77_DICPU
Clathrin adaptor, alpha-adaptin, appendage, C-terminal subdomain	IPR003164	401	1	*Dictyostelium purpureum*	5786	TR:F0ZS77_DICPU
Clathrin adaptor, alpha/beta/gamma-adaptin, appendage, Ig-like subdomain	IPR008152	1617	1	*Dictyostelium purpureum*	5786	TR:F0ZS77_DICPU
Clathrin alpha-adaptin/coatomer adaptor, appendage, C-terminal subdomain	IPR015873	913	1	*Dictyostelium purpureum*	5786	TR:F0ZS77_DICPU
Coatomer/calthrin adaptor appendage, C-terminal subdomain	IPR009028	1154	1	*Dictyostelium purpureum*	5786	TR:F0ZS77_DICPU
Coatomer/clathrin adaptor appendage, Ig-like subdomain	IPR013041	2331	2	*Dictyostelium purpureum*	5786	TR:F0ZS77_DICPU
				Paramecium tetraurelia	5888	TR:A0CBT3_PARTE
Zinc finger, N- recognin	IPR003126	1363	1	*Dictyostelium discoideum*	44689	TR:Q75JM9_DICDI
PWWP	IPR000313	2721	1	*Dictyostelium discoideum*	44689	TR:Q75JG1_DICDI
Mediator complex, subunit Med12	IPR019035	329	1	*Dictyostelium discoideum*	44689	SP:MED12_DICDI
POLO box duplicated domain	IPR000959	655	1	*Dictyostelium discoideum*	44689	SP:PLK_DICDI

Peptidase C1A, papain C-terminal	IPR000668	7839	1	*Plasmodium vivax*	126793	TR:A5KBM4_PLAVS
Peptidase C1A, papain	IPR013128	7674	1	*Plasmodium vivax*	126793	TR:A5KBM4_PLAVS
Exoribonuclease, phosphorolytic domain 1	IPR001247	12219	1	*Eimeria tenella*	5802	TR:H9B9T6_EIMTE
Ribosomal protein S5 domain 2-type fold	IPR020568	136229	1	*Eimeria tenella*	5802	TR:H9B9T6_EIMTE
Calcium-binding EF-hand	IPR002048	40446	2	*Volvox carteri*	3067	TR:D8U185_VOLCA
				Paramecium tetraurelia	5888	TR:A0BWN9_PARTE
RNA helicase, DEAD-box type, Q motif	IPR014014	29092	4	*Arabidopsis thaliana*	3702	SP:RH40_ARATH
				Arabidopsis lyrata subsp. lyrata	81972	TR:D7L5I4_ARALL
				Paramecium tetraurelia	5888	TR:A0BWN9_PARTE
				Paramecium tetraurelia	5888	TR:A0CI00_PARTE
Katanin p80 subunit B1	IPR026962	300	2	*Paramecium tetraurelia*	5888	TR:A0CXK4_PARTE
						TR:A0CYP9_PARTE
Mannose-binding lectin	IPR001229	1224	1	*Paramecium tetraurelia*	5888	TR:A0C353_PARTE
LPS-induced tumor necrosis factor alpha factor	IPR006629	860	4	*Paramecium tetraurelia*	5888	TR:A0DA79_PARTE
						TR:A0CFW6_PARTE
						TR:A0D0N7_PARTE
				Ichthyophthirius multifiliis	857967	TR:G0R2G1_ICHMG
Ubiquitin	IPR000626	10610	1	*Paramecium tetraurelia*	5888	TR:A0CBH5_PARTE
Ubiquitin supergroup	IPR019955	11848	1	*Paramecium tetraurelia*	5888	TR:A0CBH5_PARTE
Arfaptin homology (AH) domain	IPR010504	570	1	*Paramecium tetraurelia*	5888	TR:A0EC99_PARTE
Protein of unknown function DUF2816	IPR021258	73	1	*Paramecium tetraurelia*	5888	TR:Q3SDH1_PARTE
HECT	IPR000569	5084	1	*Paramecium tetraurelia*	5888	TR:A0CYJ5_PARTE
AP complex subunit beta	IPR026739	1443	1	*Paramecium tetraurelia*	5888	TR:A0CBT3_PARTE
Beta-adaptin appendage, C-terminal subdomain	IPR015151	447	1	*Paramecium tetraurelia*	5888	TR:A0CBT3_PARTE
Clathrin adaptor, beta-adaptin, appendage, Ig-like subdomain	IPR013037	343	1	*Paramecium tetraurelia*	5888	TR:A0CBT3_PARTE
MORN motif	IPR003409	4670	2	*Paramecium tetraurelia*	5888	TR:A0CR71_PARTE

						TR:A0CJB5_PARTE
Cyclic nucleotide-binding-like	IPR018490	32809	1	*Paramecium tetraurelia*	5888	TR:A0D372_PARTE
Cyclic nucleotide-binding domain	IPR000595	32468	1	*Paramecium tetraurelia*	5888	TR:A0D372_PARTE
Ion transport 2	IPR013099	9320	1	*Paramecium tetraurelia*	5888	TR:A0D372_PARTE
RmlC-like jelly roll fold	IPR014710	114755	1	*Paramecium tetraurelia*	5888	TR:A0D372_PARTE
Zinc finger, CCHC-type	IPR001878	48430	2	*Paramecium tetraurelia*	5888	TR:A0D372_PARTE
				Coccomyxa subellipsoidea C-169	574566	TR:I0Z6X5_9CHLO
Oxysterol-binding protein	IPR000648	2803	1	*Paramecium tetraurelia*	5888	TR:A0DIZ9_PARTE
Oxysterol- binding protein, conserved site	IPR018494	1938	1	*Paramecium tetraurelia*	5888	TR:A0DIZ9_PARTE
START-like domain	IPR023393	24503	1	*Paramecium tetraurelia*	5888	TR:A0DIZ9_PARTE
START domain	IPR002913	3166	1	*Paramecium tetraurelia*	5888	TR:A0DIZ9_PARTE
Nucleic acid-binding, OB-fold	IPR012340	237689	1	*Oxytricha trifallax*	1172189	TR:J9F316_9SPIT
	IPR016027	?	1	*Oxytricha trifallax*	1172189	TR:J9F316_9SPIT
IQ motif, EF-hand binding site	IPR000048	13024	1	*Oxytricha trifallax*	1172189	TR:J9ISV1_9SPIT
Zinc finger C2HC domain-containing protein	IPR026319	410	1	*Oxytricha trifallax*	1172189	TR:J9IYU2_9SPIT
Pumilio RNA - binding repeat	IPR001313	2344	1	*Oxytricha trifallax*	1172189	TR:J9I6T8_9SPIT
Epsin domain, N-terminal	IPR001026	1289	1	*Oxytricha trifallax*	1172189	TR:J9I8U5_9SPIT
Inositol polyphosphate-related phosphatase	IPR000300	2292	1	*Oxytricha trifallax*	1172189	TR:J9ETF9_9SPIT
Synaptojanin, N-terminal	IPR002013	1824	1	*Oxytricha trifallax*	1172189	TR:J9ETF9_9SPIT
Peptidase A2A, retrovirus, catalytic	IPR001995	136363	1	*Ichthyophthirius multifiliis*	857967	TR:G0R1R9_ICHMG
Aspartic peptidase	IPR021109	150638	1	*Ichthyophthirius multifiliis*	857967	TR:G0R1R9_ICHMG
	IPR009007	?	1	*Ichthyophthirius multifiliis*	857967	TR:G0R1R9_ICHMG
Aspartic peptidase, DDI1-type	IPR019103	499	1	*Ichthyophthirius multifiliis*	857967	TR:G0R1R9_ICHMG

Ubiquitin- associated/translation elongation factor EF1B, N-terminal	IPR000449	10774	1	*Ichthyophthirius multifiliis*	857967	TR:G0R1R9_ICHMG
GCN5-like 1	IPR009395	270	1	*Tetrahymena thermophila*	312017	TR:I7LT28_TETTS
Nucleotidyl transferase domain	IPR002934	11708	1	*Tetrahymena thermophila*	312017	TR:I7LWD5_TETTS
Nucleotidyltransferase, class I, C-terminal-like	IPR011068	913	1	*Tetrahymena thermophila*	312017	TR:I7LWD5_TETTS
Poly(A) polymerase, central domain	IPR007012	841	1	*Tetrahymena thermophila*	312017	TR:I7LWD5_TETTS
Poly(A) polymerase, RNA-binding domain	IPR007010	731	1	*Tetrahymena thermophila*	312017	TR:I7LWD5_TETTS
SIT4 phosphatase-associated protein family	IPR007587	715	1	*Tetrahymena thermophila*	312017	TR:Q22CQ3_TETTS
Domain of unknown function DUF221	IPR003864	1564	1	*Tetrahymena thermophila*	312017	TR:I7LUC5_TETTS
SM domain found in ataxin-2	IPR025852	451	1	*Salpingoeca rosetta*	946362	TR:F2UB05_SALS5
Autophagy- related protein 13	IPR018731	380	1	*Salpingoeca rosetta*	946362	TR:F2UPL9_SALS5
Aspartyl/Asparaginyl beta-hydroxylase	IPR007803	1500	1	*Salpingoeca rosetta*	946362	TR:F2TYA9_SALS5
Galactose-binding domain-like	IPR008979	40946	1	*Naegleria gruberi*	5762	TR:D2VQJ6_NAEGR
Major facilitator superfamily	IPR011701	206718	1	*Naegleria gruberi*	5762	TR:D2VQJ6_NAEGR
Major facilitator superfamily domain, general substrate transporter	IPR016196	288356	1	*Naegleria gruberi*	5762	TR:D2VQJ6_NAEGR
Peptidase S8/S53 domain	IPR000209	18527	1	*Naegleria gruberi*	5762	TR:D2VQJ6_NAEGR
Peptidase S8, subtilisin, His-active site	IPR022398	8940	1	*Naegleria gruberi*	5762	TR:D2VQJ6_NAEGR
Peptidase S8, subtilisin-related	IPR015500	16364	1	*Naegleria gruberi*	5762	TR:D2VQJ6_NAEGR
Copine	IPR010734	1260	1	*Naegleria gruberi*	5762	TR:D2VCR4_NAEGR
Annexin	IPR001464	2369	2	*Trichomonas vaginalis*	5722	TR:A2EUT8_TRIVA
				Daphnia pulex	6669	TR:E9GWX4_DAPPU
Forkhead-associated (FHA) domain	IPR000253	14600	1	*Sulfolobus acidocaldarius*	330779	TR:Q4J9H4_SULAC
SMAD/FHA domain	IPR008984	16138	1	*Sulfolobus acidocaldarius*	330779	TR:Q4J9H4_SULAC

Heat shock factor (HSF)-type, DNA-binding	IPR000232	2344	1	*Thalassiosira pseudonana*	35128	TR:B8LD72_THAPS
STAT transcription factor, all-alpha	IPR013801	712	1	*Branchiostoma floridae*	7739	TR:C3ZBA3_BRAFL
STAT transcription factor, coiled coil	IPR015988	691	1	*Branchiostoma floridae*	7739	TR:C3ZBA3_BRAFL
STAT transcription factor, core	IPR001217	915	1	*Branchiostoma floridae*	7739	TR:C3ZBA3_BRAFL
STAT transcription factor, DNA-binding, subdomain	IPR012345	676	1	*Branchiostoma floridae*	7739	TR:C3ZBA3_BRAFL
STAT transcription factor, protein interaction	IPR013799	652	1	*Branchiostoma floridae*	7739	TR:C3ZBA3_BRAFL
Domain of unknown function DUF4587	IPR027904	111	1	*Danio rerio*	7955	TR:Q566X1_DANRE
Not attributed				*Danio rerio*	7955	TR:F1QT44_DANRE
						TR:Q5BLC6_DANRE
						TR:F1QT44_DANRE
				Takifugu rubripes	31033	TR:Q5BU16_TAKRU
				Latimeria chalumnae	7897	TR:H3AUV9_LATCH
				Oncorhynchus mykiss	8022	TR:D3X7I3_ONCMY
				Mus musculus	10090	TR:E9Q7R5_MOUSE
						TR:Q3TT67_MOUSE
						TR:Q80X27_MOUSE
						TR:Q9D0B9_MOUSE
						TR:G5E8K1_MOUSE
				Monodelphis domestica	13616	TR:F6YC76_MONDO
				Harpegnathos saltator	610380	TR:E2B4P5_HARSA
				Solenopsis invicta	13686	TR:E9IBS4_SOLIN
				Acyrthosiphon pisum	7029	TR:J9LYX7_ACYPI
						TR:J9K6H4_ACYPI
				Drosophila virilis	7244	TR:B4LG92_DROVI
						TR:B4LT47_DROVI
						TR:B4LP27_DROVI
				Drosophila mojavensis	7230	TR:B4L247_DROMO
						TR:B4KYR0_DROMO
						TR:B4L855_DROMO
						TR:B4L698_DROMO
				Culex quinquefasciatus	7176	TR:B0XIY3_CULQU
						TR:B0X335_CULQU
				Drosophila ananassae	7217	TR:B3MYT4_DROAN
				Drosophila sechellia	7238	TR:B4IAK4_DROSE
						TR:B4HHA9_DROSE
						TR:B4HS46_DROSE
				Drosophila simulans	7240	TR:B4QGV2_DROSI

						TR:B4QJN8_DROSI
						TR:Q8ITK5_DROSI
						TR:B4Q806_DROSI
						TR:B4Q807_DROSI
				Drosophila persimilis	7234	TR:B4G4N1_DROPE
						TR:B4GID5_DROPE
				Drosophila pseudoobscura pseudoobscura	46245	TR:Q298T3_DROPS
						TR:Q28Z24_DROPS
						TR:Q29GM3_DROPS
				Drosophila yakuba	7245	TR:B4PXM1_DROYA
						TR:B4P7D2_DROYA
				Drosophila erecta	7220	TR:B3NQC8_DROER
				Drosophila melanogaster	7227	TR:J7KEC1_DROME
						TR:J7KG79_DROME
						TR:J7KH78_DROME
						TR:J7KH84_DROME
						TR:J7KEB3_DROME
						TR:J7KG95_DROME
						TR:J7KH93_DROME
						TR:J7KHA7_DROME
						TR:Q8I053_DROME
						TR:Q8I054_DROME
						TR:Q8ITL0_DROME
						TR:Q8I0J7_DROME
						TR:Q8ITK6_DROME
						TR:Q8ITK8_DROME
						TR:Q8ITK9_DROME
						TR:Q8ITK7_DROME
						TR:Q29QW0_DROME
						TR:Q9VP73_DROME
				Drosophila grimshawi	7222	TR:B4JIE8_DROGR
						TR:B4J2G2_DROGR
						TR:B4K3U5_DROGR
						TR:B4JNG4_DROGR
						TR:B4JL13_DROGR
						TR:B4JZQ4_DROGR
				Daphnia pulex	6669	TR:E9HPD0_DAPPU
				Crassostrea gigas	29159	TR:K1Q341_CRAGI
				Triticum turgidum	4571	TR:A7X9X5_TRITU
						TR:A7X9X4_TRITU
				Triticum turgidum subsp. turgidum	357456	TR:A7X9X3_TRITU
				Selaginella moellendorffii	88036	TR:D8QZB0_SELML
						TR:D8RWJ4_SELML
				Physcomitrella patens	3218	TR:A9SPD2_PHYPA
				Medicago truncatula	3880	TR:G7JYQ3_MEDTR

				Vitis vinifera	29760	TR:F6H085_VITVI
				Coccomyxa subellipsoidea C-169	574566	TR:I0Z7H7_9CHLO
				Volvox carteri	3067	TR:D8UH55_VOLCA
						TR:D8TIG8_VOLCA
				Coprinopsis cinerea	240176	TR:A8NSW4_COPC7
				Rhizopus delemar	246409	TR:I1BU39_RHIO9
				Ustilago hordei	1128400	TR:I2FNA1_USTHO
						TR:I2FX35_USTHO
				Arthroderma otae	554155	TR:C5FMN6_ARTOC
				Lodderomyces elongisporus	379508	TR:A5DX59_LODEL
				Komagataella pastoris	981350	TR:F2QZ34_PICP7
						TR:C4R811_PICPG
				Candida dubliniensis	573826	TR:B9WK29_CANDC
				Spathaspora passalidarum	619300	TR:G3AT38_SPAPN
				Pyrenophora teres f. teres	861557	TR:E3SA50_PYRTT
				Macrophomina phaseolina	1126212	TR:K2QZ41_MACPH
				Wickerhamomyces ciferrii	1206466	TR:K0KRY0_9ASCO
				Metarhizium anisopliae	655844	TR:E9EYN1_METAR
				Metarhizium acridum	655827	TR:E9E5K2_METAQ
				Gaeumannomyces graminis var. tritici	644352	TR:J3PGZ9_GAGT3
				Emericella nidulans	227321	TR:C8VHT4_EMENI
				Marssonina brunnea	1072389	TR:K1WK26_MARBU
				Mycosphaerella graminicola	336722	TR:F9XKR1_MYCGM
				Arthroderma benhamiae	663331	TR:D4AUL2_ARTBC
						TR:D4AQZ3_ARTBC
				Trichophyton verrucosum	663202	TR:D4DAF9_TRIVH
						TR:D4DE92_TRIVH
				Kluyveromyces lactis	284590	TR:Q6CV84_KLULA
				Thielavia heterothallica	573729	TR:G2QL79_THIHA
						TR:G2Q6L9_THIHA
				Ajellomyces capsulatus	447093	TR:C0NY22_AJECG
				Zygosaccharomyces rouxii	559307	TR:C5DZG0_ZYGRC
						TR:C5DPG9_ZYGRC
				Candida orthopsilosis	1136231	TR:H8XAW0_CANO9
				Chaetomium globosum	306901	TR:Q2GSW4_CHAGB
				Batrachochytrium dendrobatidis	684364	TR:F4P0I6_BATDJ
				Tuber melanosporum	656061	TR:D5GLS3_TUBMM
				Beauveria bassiana	655819	TR:J4UW00_BEAB2
				Ashbya gossypii	284811	SP:DEF1_ASHGO
				Thielavia terrestris	578455	TR:G2RF30_THITE
				Puccinia graminis f. sp. tritici	418459	TR:E3KED1_PUCGT
				Sporisorium reilianum	999809	TR:E6ZQ89_SPORE
				Capsaspora owczarzaki	595528	TR:E9CJ30_CAPO3
						TR:E9CF31_CAPO3
				Nocardia brasiliensis	1133849	TR:K0F7I8_9NOCA

				Nocardiopsis alba	1205910	TR:J7KZ35_NOCAA
				Streptomyces sp. W007	1055352	TR:D6K5N2_9ACTO
				Fischerella sp. JSC-11	741277	TR:G6FWH2_9CYAN
				Pristionchus pacificus	54126	TR:H3FY38_PRIPA
						TR:H3EBC9_PRIPA
						TR:H3DTB9_PRIPA
				Wuchereria bancrofti	6293	TR:J9EL61_WUCBA
				Caenorhabditis remanei	31234	TR:E3LNM4_CAERE
				Caenorhabditis brenneri	135651	TR:G0NPV9_CAEBE
						TR:G0NPR5_CAEBE
				Caenorhabditis briggsae	6238	TR:A8XR05_CAEBR
						TR:A8XK30_CAEBR
				Litomosoides sigmodontis	42156	TR:Q25256_LITSI
				Schistosoma mansoni	6183	TR:G4VJV4_SCHMA
				Entamoeba histolytica	5759	TR:C4M3M4_ENTHI
						TR:C4M689_ENTHI
				Entamoeba nuttalli	1076696	TR:K2GGH5_ENTNP
						TR:K2H758_ENTNP
				Entamoeba dispar	370354	TR:B0EIL3_ENTDS
						TR:B0ESJ3_ENTDS
				Polysphondylium pallidum	13642	TR:D3BG46_POLPA
						TR:D3BNS0_POLPA
						TR:D3BNM9_POLPA
						TR:D3AYF3_POLPA
				Dictyostelium fasciculatum	1054147	TR:F4PUM8_DICFS
						TR:F4QDD3_DICFS
				Dictyostelium purpureum	5786	TR:F0ZCL9_DICPU
						TR:F0ZMS5_DICPU
				Dictyostelium discoideum	44689	TR:Q86K61_DICDI
						TR:Q54PL8_DICDI
						SP:Y6864_DICDI
						SP:MED29_DICDI
				Theileria annulata	5874	TR:Q4UA28_THEAN
				Perkinsus marinus	423536	TR:C5LYD3_PERM5
				Plasmodium vivax	5855	TR:Q0PCK3_PLAVI
						TR:Q0PCK4_PLAVI
						TR:Q0PCK5_PLAVI
						TR:Q0PCK7_PLAVI
						TR:Q0PCK8_PLAVI
				Eimeria tenella	5802	TR:C8TDR1_EIMTE
				Paramecium tetraurelia	5888	TR:A0DYU8_PARTE
						TR:A0DZK0_PARTE
						TR:A0BYF9_PARTE
						TR:A0CG64_PARTE
						TR:A0C835_PARTE

						TR:A0CDZ1_PARTE
						TR:A0DHH5_PARTE
						TR:A0BJH2_PARTE
				Oxytricha trifallax	1172189	TR:J9IRJ0_9SPIT
						TR:J9IEL1_9SPIT
						TR:J9IQT3_9SPIT
						TR:J9FAN6_9SPIT
						TR:J9HYX8_9SPIT
						TR:J9IXJ9_9SPIT
						TR:J9HXG0_9SPIT
						TR:J9J1X5_9SPIT
						TR:J9J4Z1_9SPIT
						TR:J9IPS8_9SPIT
						TR:J9FLL3_9SPIT
						TR:J9I670_9SPIT
				Tetrahymena thermophila	312017	TR:Q233I4_TETTS
						TR:I7M4F5_TETTS
						TR:I7M7L9_TETTS
						TR:I7MJC7_TETTS
						TR:Q22B69_TETTS
						TR:Q23AJ1_TETTS
						TR:Q248F6_TETTS
						TR:I7MFV7_TETTS
						TR:I7MMJ5_TETTS
				Salpingoeca rosetta	946362	TR:F2TZS0_SALS5
						TR:F2U3D3_SALS5
						TR:F2UN53_SALS5
						TR:F2UG92_SALS5
						TR:F2U7C0_SALS5
						TR:F2U792_SALS5
						TR:F2TVN4_SALS5
				Naegleria gruberi	5762	TR:D2W3F9_NAEGR
				Giardia intestinalis	598745	TR:C6M0F7_GIAIB
				Trichomonas vaginalis	5722	TR:A2FXN1_TRIVA
						TR:A2G269_TRIVA
						TR:A2FUX5_TRIVA
				Trypanosoma brucei gambiense	679716	TR:C9ZUC7_TRYB9
				Trypanosoma brucei brucei	185431	TR:Q57TW9_TRYB2
				Ectocarpus siliculosus	2880	TR:D8LSY3_ECTSI
				Thalassiosira pseudonana	35128	TR:B5YLJ3_THAPS
